# Discovery of Defense- and Neuropeptides in Social Ants by Genome-Mining

**DOI:** 10.1371/journal.pone.0032559

**Published:** 2012-03-20

**Authors:** Christian W. Gruber, Markus Muttenthaler

**Affiliations:** 1 Medical University of Vienna, Center for Physiology and Pharmacology, Vienna, Austria; 2 Departments of Chemistry and Cell Biology, The Scripps Research Institute, La Jolla, California, United States of America; James Cook University, Australia

## Abstract

Natural peptides of great number and diversity occur in all organisms, but analyzing their peptidome is often difficult. With natural product drug discovery in mind, we devised a genome-mining approach to identify defense- and neuropeptides in the genomes of social ants from *Atta cephalotes* (leaf-cutter ant), *Camponotus floridanus* (carpenter ant) and *Harpegnathos saltator* (basal genus). Numerous peptide-encoding genes of defense peptides, in particular defensins, and neuropeptides or regulatory peptide hormones, such as allatostatins and tachykinins, were identified and analyzed. Most interestingly we annotated genes that encode oxytocin/vasopressin-related peptides (inotocins) and their putative receptors. This is the first piece of evidence for the existence of this nonapeptide hormone system in ants (Formicidae) and supports recent findings in *Tribolium castaneum* (red flour beetle) and *Nasonia vitripennis* (parasitoid wasp), and therefore its confinement to some basal holometabolous insects. By contrast, the absence of the inotocin hormone system in *Apis mellifera* (honeybee), another closely-related member of the eusocial Hymenoptera clade, establishes the basis for future studies on the molecular evolution and physiological function of oxytocin/vasopressin-related peptides (vasotocin nonapeptide family) and their receptors in social insects. Particularly the identification of ant inotocin and defensin peptide sequences will provide a basis for future pharmacological characterization in the quest for potent and selective lead compounds of therapeutic value.

## Introduction

Natural peptides of great number and diversity occur in all organisms from microbes to plants to animals and exhibit biological activity often against unrelated targets. This provides researchers with excellent starting points for drug discovery [1], given that it is possible to isolate and characterize these natural peptides in adequate quantities or to retrieve their amino acid sequence genetically for synthetic production and biological testing. Peptidomics, using state-of-the-art liquid chromatography and mass spectrometry technologies, is generally the method-of-choice to identify and characterize peptides on protein level, whereas this technique yet fails to accurately identify the ‘peptidome’ from complex sample mixtures [2], [3], [4] or when the sample amount is limited or difficult to obtain, for instance peptides that are produced by mandibular- or venom glands of some insect species [5], [6], [7]. This applies in particular to ants, which are, due to their limited body and organ size, difficult to screen by analytical instrumentation unless many thousand individuals are sacrificed or laborious venom sac dissection is being used [5], [8]. Other problems associated with peptidomics is the retrieval of low abundant peptides in complex mixtures and the detection of pseudo-gene products, i.e. peptide coding genes that have been switched off during evolution, but which may encode bioactive drug leads [9], [10].

Genome-mining, a term that has been used to describe the exploitation of genomic information for the discovery of new processes, targets, and products [11], may be a useful alternative or complement to peptide discovery by peptidomics. This technique seems in particular valuable in the genomic era, since the number of available genomes is steadily increasing as whole genome sequencing is becoming affordable and achievable. Following the footsteps of the human genome initiative [12] and many other successful genome-sequencing efforts in animals, plants and microbes, recently the genomes of seven ant species have been reported. These include the invasive Argentine ant *Linepithema humile* [13], the red harvester ant *Pogonomyrmex barbatus* [14], the fire ant *Solenopsis invicta* [15], the carpenter ant *Camponotus floridanus* and a basal ant *Harpegnathos saltator* [16], as well as the leaf-cutter/farming ants *Atta cephalotes* [17] and *Acromyrmex echinatior* [18], respectively.

**Figure 1:**
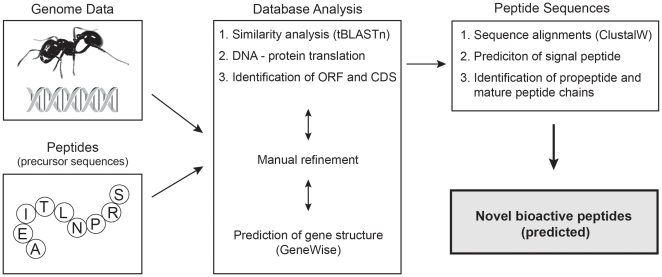
Flowchart of genome-mining for the discovery of ant peptides used in this study. Whole genome shotgun data, in this instance from the three ant species *Atta cephalotes*, *Camponotus floridanus* and *Harpegnathos saltator*, and amino acid sequences of precursor proteins from bioactive peptides of interest (*e.g.* defense and/or regulatory neuropeptides) were used for database analysis. This included similarity analysis of target DNA sequence and query protein sequence using tBLASTn, DNA to protein translation of discovered hit sequences and identification of open reading frames and coding sequence. The obtained automated results were refined and confirmed manually and used for gene structure prediction using the GeneWise algorithm. Database analysis yielded precursor protein and peptide sequences that were further annotated and analyzed by sequence alignments and similarity comparison to identify signal sequences, propeptides and mature peptide chains. Using this genome-mining methodology it was possible to predict the amino acid sequences of bioactive peptides in ants.

The aim of this study was to analyze three representative ant genomes from the subfamilies of Myrmicinae (*A.cephalotes*), Formicinae (*C.floridanus*) and Ponerinae (*H.saltator*) for the discovery of peptide encoding genes and their sequences using an array of publicly available tools, including tBLASTn similarity search, GeneWise gene structure prediction and ClustalW sequence alignments (Figure 1). Using this methodology it was possible to identify numerous putative peptides as partial, full-length precursor and mature amino acid sequences of ant defense- and neuropeptides. These genes were characterized by similarity to other insect and non-insect species and for the first time we report the sequences of inotocin peptides (oxytocin/vasopressin-related neuropeptides) in social ants. The presented results offer the possibility to interpret the phylogenetic relationship and evolution of insect defense molecules and peptide hormone systems, but most importantly the predicted mature peptide sequences could provide novel drug leads or tools to study the similar and preserved receptor systems in humans.

## Results and Discussion

Genome-mining is a powerful technique to discover novel putative bioactive peptides, in particular when the peptidome of interest is not readily accessible by modern analytical instrumentation. This study was designed to explore the genomes of three recently reported social ant species for the discovery of defense- and neuropeptide sequences, which are listed in Table 1.

**Table 1:** Selected defense- and neuropeptides from ant species characterized by genome-mining.

peptide class	species/peptide name	length amino acids (precursor/mature)	no. of Cys	evidence		
				precursor	mature peptide	partial
**Abaecin** +	*A.cephalotes* ABA-like	52/33	*n.a.* *	√	√	
	*C.floridanus* ABA-like	-	*n.a.*			√
	*H.saltator* ABA-like	50/31	*n.a.*	√	√	
**Allatostatin**	*A.cephalotes* AST	193/8, 8, 8, 7, 30, 9, 28#	*n.a.*	√	√	
	*C.floridanus* AST	193/8, 8, 8, 7, 30, 9, 27#	*n.a.*	√	√	
	*H.saltator* AST	193/8, 8, 8, 7, 30, 9, 25#	*n.a.*	√	√	
**Defensin** +	*A.cephalotes* DEF	97/43	6	√	√	
	*C.floridanus* DEF1	97/43	6	√	√	
	*C.floridanus* DEF2	98/40	6	√	√	
	*H.saltator* DEF1	100/43	6	√	√	
	*H.saltator* DEF1a	77/43	6	√	√	
	*H.saltator* DEF2	-/41	5$		√	√
**Eclosion hormone** +	*A.cephalotes* EH-like+	-	*n.a.*			√
**Diuretic-hormone** +	*A.cephalotes* DH-like	-	*n.a.*			√
	*C.floridanus* DH-like	-/31	*n.a.*		√	√
	*H.saltator* DH-like	-	*n.a.*			√
**Inotocin/AVP-like** +	*A.cephalotes* INT/AVP-like&	148/9	2+12	√	√	√
	*C.floridanus* INT/AVP-like&	-/9	2		√	√
	*H.saltator* INT/AVP	150/9	2+12	√	√	
**Ion-transport and CHH-like peptide** +	*A.cephalotes* ITP-like	-/72	6		√	√
	*C.floridanus* ITP-like	-	-			√
	*C.floridanus* CHH-like	-	-			√
	*H.saltator* ITP-like	157/72	6	√	√	
**Neuroparsin-A** +	*A.cephalotes* NP-like	-	*n.a.*			√
**Pilosulin**	*C.floridanus* PIL-like	87/multiple mature peptides**		√	√	√
**Tachykinin-like**	*A.cephalotes* TRP-like	484/9##	*n.a.*	√	√	√

* not applicable, peptide class generally contains no cysteines in the mature peptides;

# multiple mature allatostatin peptides are encoded by the same precursor protein, order of presented length of peptides are in order as presented in Figure 4;

$ 5 cysteines were identified in the mature form of *H.saltator* DEF2, which likely indicates a partial sequence;

& GeneWise prediction was no successful, but precursor sequences could be established manually from tBLASTn results;

+ during the preparation/revision of this manuscript the following peptide-/receptor sequences (partial or complete) were released on UniProtKB: diuretic hormones (*C.floridanus*: tr|E2AZE8; *H.saltator*: tr|E2C6V6, tr|E2B7W2), vasotocin-neurophysin (*H.saltator*: EFN79183), eclosion hormones (*C.floridanus*: tr|E2AXD4; *H.saltator*: tr|E2BSX6), ion-transport peptides (*C.floridanus*: tr|E2AP65; *H.saltator*: tr|E2BEL2), neuroparsins-A (*C.floridanus*: tr|E1ZXL4; *H.saltator*: tr|E2BLJ9), abaecin (*H.saltator*: tr|E2B7M5) and defensins (*C.floridanus*: tr|E2AKI0, tr|E2AVT3; *H.saltator*: tr|E2BDP6), for reference see [16];

** multiple mature peptides can be cleaved from the same precursor protein, see [33], [35], [36];

## eight tachykinin peptides of equal length are encoded by the precursor peptide, for order see Figure S5.

### Identification of ant defense peptides

Ants belong to the class of eusocial insects and live in crowded nests. Millions of individuals are in close interaction and hence it is not surprising that they have evolved a highly developed system of immune response to fight pathogen infections [16]. One of the best studied class of innate immune molecules are the so-called defensin peptides, which occur in many if not all organisms [19]. Several ant defensins have been reported so far [19], [20], [21], [22], [23], mainly by genomic sequencing [23], since it seems extremely difficult to isolate and identify these peptides by tandem mass spectrometry or Edman degradation. Using the proposed genome-mining methodology, it was possible to identify sequences of defensins and several related defense peptides and peptide toxins in all three ant species (Table 1, Table S1).

#### Structural characteristics of putative ant defensin precursors and mature peptides

**Figure 2:**
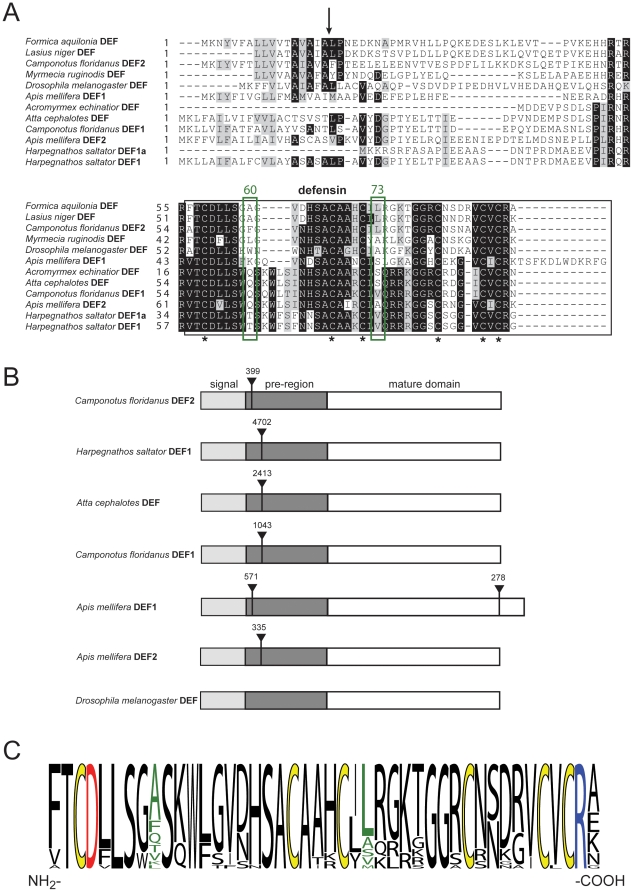
Sequence and gene structure of novel ant defensins. (**A**) Identified ant defensin precursor sequences from *Atta cephalotes*, *Camponotus floridanus* and *Harpegnathos saltator* were used for similarity alignment (ClustalW2) and compared to known defensins from *Formica aquilonia* (UniProtKB Q5BU36), *Lasius niger* (B9TXS0), *Myrmecia ruginodis* (B9TXS6), *Drosophila melanogaster* (P36192), *Apis mellifera* (P17722, Q5MQL3) and *Acromyrmex echinatior* (F4WLL3). The signal peptide cleavage site (identified by similarity) is shown as an arrow. Mature defensin peptides are indicated in the black box and the conserved cysteine residues are indicated with asterisks. Residue positions of positive evolutionary selection [23] are indicated with a green box. The sequence alignment was prepared using Boxshade. (**B**) Gene structure of novel ant and known insect defensin genes (GenBank *D.melanogaster* NT033778.3 and *A.mellifera* NC007085.3, NC007075.3) was predicted using the GeneWise algorithm. Signal sequences are indicated in light grey, pre-regions in dark grey and the mature peptide domain in white. Intron sequences (including their base pair length) are indicated with upside-down arrow heads. (**C**) The sequences of 22 known [23] and the novel ant defensins have been compared using a sequence logo to highlight their amino acid variation. Conserved cysteines are colored in yellow, the positions of positive evolutionary selection are colored in green and the conserved negatively-charged Asp and positively-charged Arg are colored in red and blue, respectively.

Using the amino acid sequences of the putative ant defensin genes we were able to compare their molecular characteristics to known insect defensins. Figure 2 shows the defensin sequences in alignment with selected ant and insect defensins from *Apis mellifera* and *Drosophila melanogaster* (Figure 2).

The peptides share common molecular characteristics with other insect defensins, i.e. (i) a similar length of precursor protein and mature peptide (ranging from 40–43 amino acids; Table 1), (ii) a conserved network of six cysteine residues and (iii) a strong positive net-charge of the mature peptides (*A.cephalotes* DEF = +6, *C.floridanus* DEF1 = +5, *C.floridanus* DEF2 = +3, *H.saltator* DEF1 = +5) to interact with and disrupt negatively-charged microbial membranes. This strong positive charge is ‘neutralized’ by an anionic pro-domain (*A.cephalotes* DEF = −4, *C.floridanus* DEF1 = −3, *C.floridanus* DEF2 = −3, *H.saltator* DEF1 = −4) to prevent toxic effects to the cells during defensin biosynthesis. This charge correlation between the prodomains and the mature defensin is well known to exist for many defensins, including mammalian defensins and it appears to be conserved throughout this class of peptides [24].

**Figure 3:**
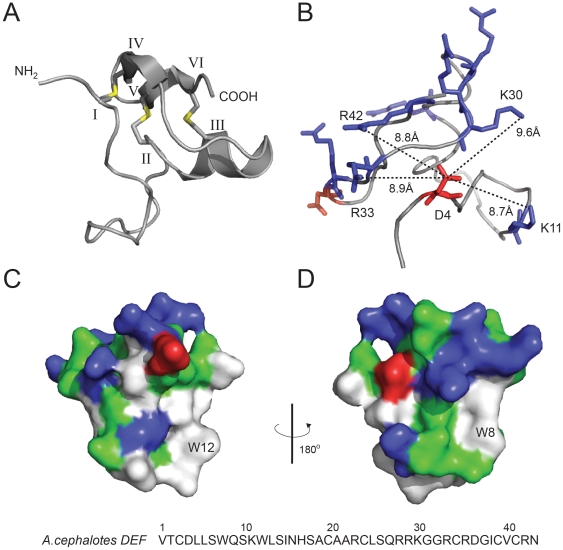
Structural model of *Atta cephalotes* defensin. The lowest energy model of the ant defensin was prepared by homology to the insect defensin phormicin (pdb code 1ICA) and the synthetic defensin Def-BBB (2E3E). (**A**) The structure cartoon shows the three conserved disulfide bonds (in yellow) and secondary structure elements (loops, α-helix, and two anti-parallel β-sheets) that form together the so-called ‘cysteine-stabilized αβ (CSαβ) motif’. The cysteines are labeled with roman numerals C_I_–_VI_. (**B**) The putative mature peptide sequence contains two negatively-charged (shown in red) and eight positively-charged (shown in blue) residues. The distances between the side chains of the conserved Asp (D4) and surrounding Lys- (K11 and K30) and Arg-residues (R33 and R42) were measured using PyMol and are indicated by dotted lines (in Å units). The measured distances and orientations of the side-chains suggest possible electrostatic interactions (salt-bridge formation) between these residues. (**C, D**) Surface representation of the ant defensin pointing out its amphipathic character, *i.e.* charged surface (cations are shown in blue, anions in red) in combination with a hydrophobic patch (shown in white) that contains two tryptophan-residues (W8 and W12). The amino acid sequence including residue numbers of the mature *A.cephalotes* defensin is shown below the structural models.

Besides the cysteine residues there are at least two more residues that appear highly conserved amongst ant defensins, namely the negatively-charged aspartic acid residue at the beginning (pos. 4) and the positively-charged arginine residue (pos. 42) at the end of the mature domain (Figure 2C). Similarly, mammalian α-defensins contain oppositely-charged residues that form a highly conserved salt-bridge interaction, which is critical for the formation of the disulfide bonds, structural rigidity, and biological function [25], [26]. To gain further insight into the role these conserved residues play in folding and stability of ant defensins, we prepared a homology structure model of the representative *A.cephalotes* defensin. The peptide sequence was modeled by energy minimization on distance restraints to the closely related structures of the insect defensin phormicin [27] and a synthetic defensin analogue (DEF-BBB, [28]). The model strongly suggests the presence of a so-called ‘cysteine-stabilized αβ (CSαβ) motif’ whose C_I_–C_IV_, C_II_–C_V_ and C_III_–C_VI_ pairing forms three intramolecular disulfide bonds, which is a characteristic for other insect defensins (Figure 3A) [27]. Furthermore, according to the NMR structures (pdb codes 1ICA and 2E3E, respectively) that were used as templates, the loop between the first two cysteine residues (C_I_ and C_II_, see Figure 3A) is mainly disordered. To analyze the possible formation of a functionally important salt-bridge interaction, we calculated the distances of the side-chain of the conserved aspartic acid residue to neighboring side-chains of positively-charged residues. The charged side-chains of Asp 4 and Arg 42 are ∼8.8 Å apart from each other (Figure 3B) and although the conserved Asp 4 does not form a salt bridge in the homology model or in the template structure on which it was based, several positively-charged residues (Lys 11, Lys 30, Arg 33 and Arg 42) are within close enough proximity (<10 Å) to be potentially important for the formation of electrostatic interactions. However, structural studies would be required to confirm whether this is actually the case. In addition to the charge distribution and potential electrostatic interactions, we analyzed the surface characteristics and it is obvious (Figure 3C and D) that the ant defensin contains many hydrophobic residues (∼30%), which seem to form a hydrophobic surface patch; in particular, the model points out two tryptophan residues (Trp 8 and Trp 12) that appear on the surface of the molecule. The overall amphipathic character of the mature peptide, *i.e.* combination of charged and hydrophobic surface, is common to many defensins and presumably contributes to their ability to insert into and disrupt microbial cell walls. In summary, the sequence and surface characteristics (charge distribution, hydrophobic patch, and amphipathic surface) and structural characteristics (CSαβ-motif, potential salt-bridge interaction) of the putative ant defensin appear to be in agreement with known defensins and it would be interesting to assess their biological activity in future studies.

#### Evolutionary analysis of putative ant defensins

Viljakainen and Pamilo have recently analysed ant defensin precursor sequences and identified two positions in the mature peptide domain that show positive evolutionary selection (green box in Figure 2A) [23]. These positions are characterized by non-synonymous substitutions to yield a higher rate of amino acid variation, as compared to the remaining protein sequence. The defensin sequences from *A.cephalotes* (DEF) and *C.floridanus* (DEF1) have a glutamine in pos. 60 (named according to [23]) and *A.cephalotes* (DEF), *C.floridanus* (DEF1) and *H.saltator* (DEF1) contain a serine or valine, respectively, in pos. 73. This sequence variation has been highlighted in a sequence logo (Figure 2C). These residues represent novel amino acid variations and hence support the hypothesis by Viljakainen and Pamilo that the immune system of social ants and dipteran insects may have responded differently to selection pressure caused by microbes and pathogens [23]. Another feature of the putatively identified ant defensins is their gene structure, which differs in the length and position of introns and exons compared to other insect defensins from *A.mellifera* and *D.melanogaster* (Figure 2B). For example, the *A.mellifera* DEF1 and *C.floridanus* DEF2 share the same position and a similar length of the first intron, but the honeybee gene contains an additional second proximal intron, whereas no introns could be identified for the *D.melanogaster* DEF gene. Similarly, *A.mellifera* DEF2, *A.cephalotes* DEF, *C.floridanus* DEF1 and *H.saltator* DEF1 share a similar intron position, whereas the ant defensin introns tend to be much longer (*e.g.* 4702 bases in leaf-cutter ant *vs.* 335 bases in honeybee). These differences support the suggestion that defensin evolution may be taxon specific [23], [29].

#### Discovery of related ant defense peptides

Besides the discovery of defensin genes we have analyzed the three ant genomes for the presence of many other defense peptides and peptide toxins using tBLASTn and found genetic evidence of at least five different classes of ant/insect defense and defense-related peptides (see Table S1). Amongst those are defensins (as discussed above), abaecins, apidaecin-related peptides, hymenoptaecins and pilosulins. Exemplarily, molecular structures of pilosulin and abaecin defense peptides were analyzed in more detail. Pilosulins are allergenic peptides with immunoglobulin-binding activity that are commonly found in venoms of *Myrmecia spp.* [30], [31], [32], [33]. The present genomic screen identified a pilosulin-like peptide in *C.floridanus* (Figure S1A). Pilosulins share a high sequence similarity in their first 47 amino acid residues (including the signal peptide), but the mature peptide domain varies substantially amongst pilosulins [34], [35], [36], [37]. This similarity supports the results of our analysis; however, at this stage it has to be considered with caution and needs to be confirmed on transcriptional level, since *C.floridanus*, like all Formicinae species, has a significantly reduced sting and venom reservoir exclusively for the production of formic acid.

Another class of defense peptides that have been identified in this screen are abaecins. Abaecin peptides are considered to be major antibacterial response peptides that have been originally discovered in honeybee [38] and occur in several ant species that have been analyzed, including the putative abaecin peptide sequences found in *A.cephalotes*, *C.floridanus* and *H.saltator* (Figure S1B). All sequences share the common proline-rich characteristic, *i.e.* they contain between 9 and 10 Pro-residues (∼30% proline content). Besides the reported genetic evidence of putative ant defense peptides, the main focus of this study was the analysis of the three ant genomes for the presence of neuropeptides and regulatory peptide hormones.

### Identification of ant neuropeptide- and regulatory peptide hormone-encoding genes

Neuropeptides and regulatory peptide hormones control many, if not all important developmental, physiological and behavioral processes in animals, including insects [39]. In the following we describe the characterization of several ant neuropeptides and regulatory peptide hormones, in particular oxytocin (OT) and arginine-vasopressin (AVP)-related peptides and interpret the importance of these findings on a molecular and phylogenetic level.

#### Genetic evidence for the existence of an oxytocin/vasopressin-related hormone system in social ants

**Figure 4:**
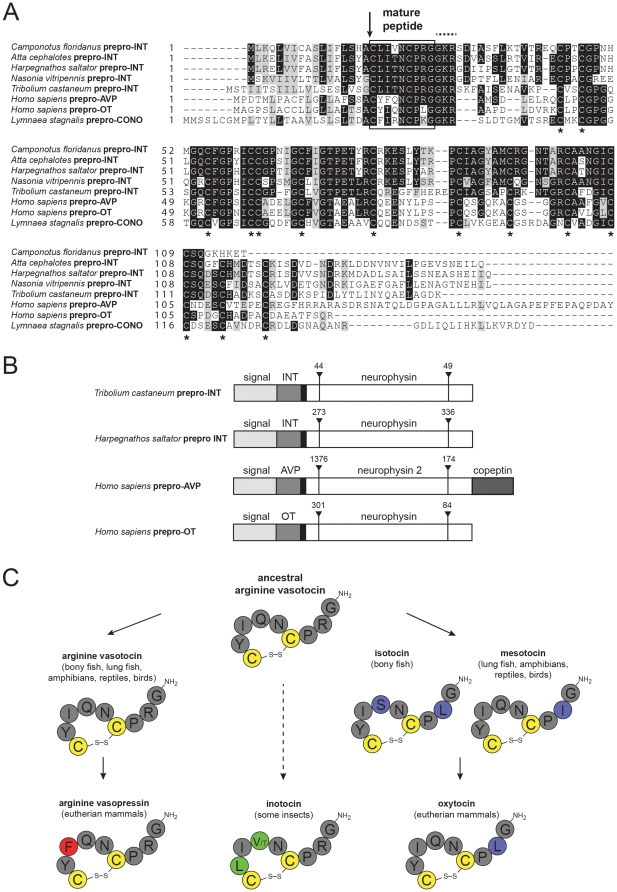
Sequences, gene- and peptide structures of ant inotocins. (**A**) Sequences of prepro-inotocin/neurophysin proteins from *Atta cephalotes*, *Camponotus floridanus* and *Harpegnathos saltator* were compared by similarity alignment to known inotocin/oxytocin/vasopressin prepro-proteins from *Tribolium castaneum* (UniProtKB A3RE83), *Nasonia vitripennis* (GenBank XP001606547.1), *Homo sapiens* (P01185 and P01178) and *Lymnaea stagnalis* (Q00945). The signal peptide cleavage site (identified by similarity) is shown with an arrow. Mature vasopressin/oxytocin/inotocin peptides are indicated in the box, followed by the canonical GRK amidation signal (dotted line above the sequences). The conserved cysteine residues in the neurophysin domain are indicated with asterisks. The sequence alignment was prepared using Boxshade. (**B**) Gene structure of novel *H.saltator* inotocin and known vasopressin-family prepro-protein genes (GenBank *H.sapiens* NC000020.10 and *T.castaneum* NC007423.2) was predicted using the GeneWise algorithm. Signal sequences are indicated in light grey, the mature peptide hormone chains (INT, inotocin; AVP, vasopressin; OT, oxytocin) in dark grey, pre-regions in black and the neurophysin domains in white. For the AVP prepro-protein the copeptin region is also marked. Intron sequences (including their base pair length) are indicated with upside-down arrow heads. (**C**) Evolution of the vasotocin nonapeptide family (simplified illustration for clarity, see also [42], [46]) is indicated by solid arrows. Arginine-vasotocin is the presumed ancestral peptide of oxytocin and vasopressin. Mammalian oxytocin evolved via intermediate forms of isotocin (bony fish) and mesotocin (lung fish, amphibians, reptiles and birds). It is yet to be determined whether invertebrate oxytocin/vasopressin-related peptides in insects or snails (*e.g.* conopressins, not shown) have also evolved from ancestral vasotocin (indicated as dashed line) [42]. The peptide sequences are shown in one-letter amino acid code. The highly conserved cysteine-residues and disulfide bonds are colored in yellow. Residues in the ancestral arginine-vasotocin and those that are identical to vasotocin are colored in dark grey. Residues that have changed during vasopressin evolution are colored in red, residues that have changed during oxytocin evolution are colored in purple and residues that are unique to insect/ant inotocins are colored in green.

The origination of the OT/AVP peptide hormone system is considered to date back 640–760 million years ago [40], [41]. All vertebrate OT/AVP-like peptides are considered to have evolved from the ancestral nonapeptide arginine-vasotocin (AVT) [42] and are today present in many different species, including non-mammalian vertebrates, fish, mammals and humans [43], [44] (Figure 4C). AVT is structurally similar to a variety of invertebrate nonapeptides [45], suggesting that the AVT-like invertebrate nonapeptides are much more ancient than AVT itself [46], [47]. AVT-like nonapeptides are present in several invertebrate species, including molluscs and annelids and they have been characterized in the arthropods *Locusta migratoria*, in the red flour beetle *Tribolium castaneum* and the parasitic wasp *Nasonia vitripennis* [41], [48].

The ant genome analysis revealed the presence of prepro-inotocin proteins in all three species (Figure 4). The precursor proteins of the ant inotocins described here, other insect inotocin proteins, snail conopressin and human OT/AVP precursor proteins all share molecular features. Following the mature nonapeptides, they all contain the canonical GRK amidation signal and they all contain 12 conserved Cys-residues in the neurophysin domain (Figure 4A). This similarity is supported by their gene structure, since *T.castaneum*, *H.saltator* and the human prepro-OT gene share identical intron sites and similar lengths (Figure 4B). The mature peptides have the same length and position of Cys-residues, but the molecular sequence is slightly different between species (Figure 4C). Both newly identified ant inotocin sequences display high similarity to AVT [42]. The novel sequences show amino acid variations in pos. 2 (polar Tyr replaced by hydrophobic Leu) and pos. 4 (polar Gln replaced by polar Thr or hydrophobic Val, respectively). Structure-activity studies should reveal if these modifications display novel selectivity on the human OT/AVP receptors. Generally it is anticipated that AVT-like sequences observed in nature should have a selective advantage over random synthetic libraries due to the importance and preservation of the β-turn motif crucial for receptor binding (summarized in [1]).

The existence of the inotocin hormone system in ants is supported by the presence of DNA sequences similar to the inotocin receptor sequence from *T.castaneum* (NP001078830.1), i.e. tBLASTn similarity analysis using the WGS sequences of *A.cephalotes*, *C.floridanus* and *H.saltator* yielded putative inotocin receptor sequences of 33–51%, 66% and 41–64% amino acid identity, respectively (File S1). Despite this genetic evidence, the receptor sequences need to be fully annotated and their biosynthesis confirmed on transcriptional and protein level in future studies. In agreement with our observations, vasopressin-family receptors have been independently annotated during the genome sequencing project of *C.floridanus* and *H.saltator* [16] and one of the receptor sequences was released on UniProtKB (tr|E2C6R3) during the preparation/revision of this manuscript.

**Figure 5:**
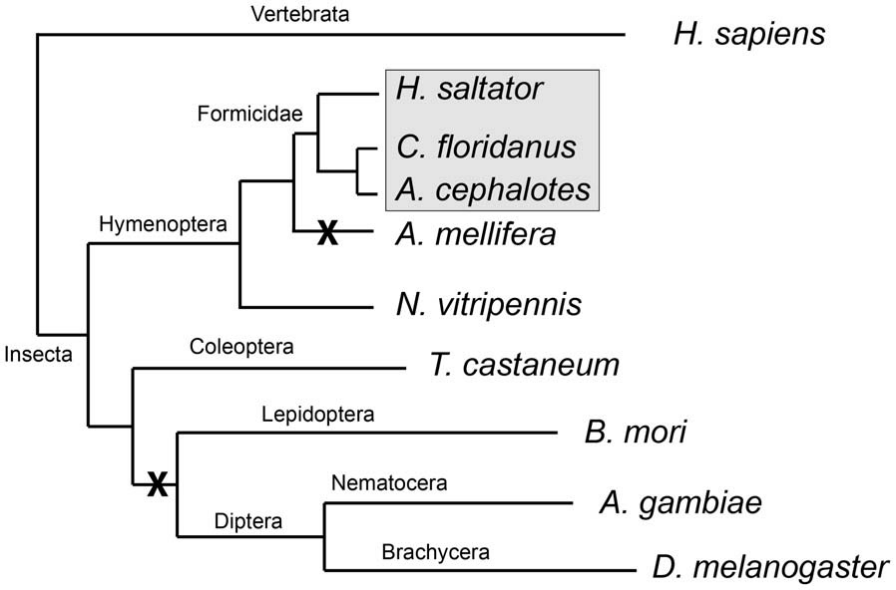
Phylogenetic relationship of selected ant and insect species. The phylogenetic relationship of the ant species (Formicidae) used for this study (*Atta cephalotes*, *Camponotus floridanus* and *Harpegnathos saltator*; shown as grey box) is indicated as a phylogram tree (adapted and modified from [16], [66]) in comparison to two Hymenoptera species *Apis mellifera* (honeybee) and *Nasonia vitripennis* (parasitoid wasp), the Coleoptera species *Tribolium castaneum* (red flour beetle), the Nematocera species *Anopheles gambiae* (mosquitoes), the Brachycera species *Drosophila melanogaster* (fruit fly) and human as representative vertebra species (*Homo sapiens*). The absence of the oxytocin/vasopressin peptide hormone system in a specific lineage is indicated with an X.

The presence of OT/AVP-like peptides and their putative receptors in ants is also interesting from an evolutionary perspective, since the homologues sequences are absent in the genomes of the fruit fly (Brachycera), mosquito (Nematocera), silkworm (Lepidoptera) and honeybee (Hymenoptera) [41], [48], whereas they are present in the red flour beetle (Coleoptera), the parasitic wasp and the three ant species (both Hymenoptera) as described above (Figure 5). The novel discoveries confirm the recent findings of the existence of the OT/AVP receptor system in arthropods and their confinement to some basal holometabolous insects (*i.e.* Hymenoptera and Coleoptera species). As indicated in Figure 5 the OT/AVP peptide hormone system has been lost at least two times during holometabolous insect evolution. However, the question of how the (predicted) existence of this neuropeptide system in ants (Formicidae) and its absence in honeybees (Apidae) can be explained remains, since both insect families belong to the eusocial Hymenoptera clade (monophyletic lineage Aculeata). The answer to this is beyond the scope of this paper, but needs to be studied in detail after the peptides and their receptors have been confirmed and their function tested.

#### Identification and analysis of other ant neuropeptide- and peptide hormone genes

As mentioned above, ants as well as honeybees belong to the eusocial Hymenoptera clade and are considered as being advanced over other insects in terms of learning, navigation and behavior [49], [50]. To reveal genes that are involved in higher brain functions and sociality, Hauser *et al.* compared neuropeptide and protein hormone genes and their receptors from honeybee and fruit fly [50]. Although there are differences in (i) the overall number of identified ligands and receptors for each species and (ii) the presence/absence of certain ligand/receptor-systems in one *versus* another species, the general conclusion of the comparison is coevolution of neuropeptide and protein hormone ligands and their receptors in both species.

Besides OT/AVP-related peptides and their putative receptors, we analyzed the three ant genomes for the presence of several other neuropeptides and peptide hormones (Table S2). From the genome sequencing project of *C.floridanus* and *H.saltator* [16] genetic evidence of the following neuropeptide ligands amongst others has been reported: Pro-corazonin (UniProtKB tr|E2ARW3 and tr|E2B7L4), FMRFamide-related peptide (tr|E2A009), orcokinin (tr|E1ZVK3 and tr|E2BU65), pheromone biosynthesis-activating peptide (tr|E2B2R9), eclosion-hormone (tr|E2AXD4 and tr|E2BSX6), neuroparsin-A (tr|E1ZXL4 and tr|E2BLJ9) and latrophilin-3 (tr|E2A464). A tBLASTn analysis (using different insect query sequences) in our study indicated the presence of those peptide-encoding genes also in *A.cephalotes*; furthermore, we independently found genetic evidence of several neuropeptides and peptide hormones, such as allatostatin, bombyxin, diuretic hormone, ecdysis-triggering hormone, hypertrehalosaemic hormone, ion-transport peptide, myosupressin, short neuropeptide F, neuropeptide Y-like, Nogo-B like peptide, queen-brain selective protein-1, parathyroid hormone-related peptide, pigment dispersing factor, prohormones 1–4, sulfakinin and tachykinin. (Table S2). These findings are generally in agreement with those from Bonasio *et al.* [16].

There are a few neuropeptides that have been lost in ants compared to other insects. Analysis of the three ant WGS sequences (*A.cephalotes*, *C.floridanus* and *H.saltator*) did not yield genetic evidence for precursor proteins of allatotropin, neuropeptide F, sialokinin, protein hugin, cardioactive/cardio-acceleratory peptide and prothoracicotropic hormone (see Table S2, [16]). The lack of genetic evidence for the above mentioned neuropeptide ligands may have several reasons, *i.e.* the current peptide hormone system does not exist in ants or the genome-mining approach is not sensitive enough to detect the genes for those ligands due to lack of similarity or contig arrangement. For example, sialokinin is a tachykinin-like peptide, which has only been reported in *Aedes aegypti* and it is therefore not surprising that this ligand does neither exist in ants nor in honeybee or fruit fly. On the other hand, neuropeptide F and proctolin have also not been found in honeybee, another Hymenoptera species, but they do exist in fruit fly (Brachycera). To obtain more detailed information about the evolutionary conservation of certain neuropeptides in insects, it will be necessary to perform a thorough comparative genomics analysis of several insect species once the annotations of ligands and receptors are available.

Exemplarily, the amino acid sequences and/or gene structures of allatostatins (Figure S2), diuretic hormone and ion-transport-like peptides (Figure S3), neuroparsin- and eclosion hormone-like peptides (Figure S4) and tachykinin peptides (Figure S5) were analyzed in more detail. The molecular features of these peptides have been summarized in Table 1; generally, they share high sequence similarity to their insect orthologs. Allatostatins and tachykinins are short amidated peptides and act similarly to OT/AVP on G-protein coupled receptors (GPCR). For both peptide ligands we provide genetic evidence (tBLASTn) for the respective receptors in ants (File S1) and confirmed earlier findings [16]. Allatostatins are neuropeptides found in insects and other invertebrates, which function *inter alia* as inhibitors of juvenile hormone synthesis and hence are important regulators of development and reproduction [51]. On the other hand, tachykinins represent one of the largest neuropeptide families and are widely distributed in animals from the lowest invertebrates to humans. It has been recognized that tachykinins have a variety of effects in physiological and pathological conditions [52]. Not only due to their suggested intrinsic neuroprotective and neurodegenerative properties, many tachykinin peptides are under investigation as templates in drug discovery and development for neurological disorders [53].

Other examples of physiologically important insect peptides are diuretic hormones and ion-transport peptides. Diuretic hormone peptides regulate water balance in insects and belong to either of three families, namely corticotropin-releasing factor-related peptides, calcitonin-like peptides or kinin peptides [39]. It occurs that all three classes of diuretic hormone peptides are genetically present in the three ant species (Table 1, Table S2, [16]) and based on the comparison of the putative mature peptide sequences to *Drosophila melanogaster* (Figure S3), they appear conserved within insects. More evidence for the presence of the diuretic hormone system in ants has been added with the report of the sequences for the diuretic hormone receptors from *H.saltator* (tr|E2C6V6, tr|E2BIN7) and *C.floridanus* (tr|E2B0Y7). On the contrary, ion-transport peptides stimulate ion and water reabsorption from the ileum and act as anti-diuretic hormones [39]. The annotated putative mature ion-transport peptides (Figure S3) share high similarity to other insect peptides from Orthoptera, Lepidoptera and Hymenoptera and hence appear evolutionary conserved within insects.

We are aware that this information is preliminary until the discovered genes have been confirmed on a transcriptional and/or peptide level. Furthermore, the receptors for all reported ant peptides need to be annotated and analyzed in more detail since the identification of the receptors constitutes an essential step in the definition of a ligand/hormone-receptor system. Nevertheless, due to the highly topical interest in ant genome research [54] and peptide drug discovery from nature [1], it is intriguing to interpret the findings from an evolutionary and drug discovery point of view; these putative peptide ligands may provide blueprints to devise novel tools for neuroscientists, maybe even initial drug leads, and it is anticipated that genome-mining of evolutionary selected species will turn out more efficient than random synthetic chemical library approaches.

### Opportunities for peptide drug discovery and medicinal chemistry

Nature's diversity has long been and still is one of the biggest resources of pharmaceutical lead compounds and many natural products often exhibit biological activity against unrelated biological targets, thus providing starting points for drug development [1]. In particular, natural peptides of great number and diversity occur in all organisms from microbes to insects to vertebrates [1] and some of them have already successfully made it into the clinic, including Prialt®, a cone snail venom peptide for the treatment of chronic pain, and Byetta®, an anti-diabetic glucagon-like peptide isolated from glia monster.

The discovery of OT/AVP-like neuropeptides in these ant species is of special interest due to an ongoing program on OT/AVP drug discovery in our lab. OT and AVP are closely related, highly conserved, multifunctional neurohypophyseal peptides. In humans and other mammalian species, these nonapeptides mediate a range of peripheral and central functions (summarized in [1]) by signaling through four GPCRs (OTR, V_1a_R, V_1b_R and the V_2_R). The high extracellular receptor homology and ubiquitous receptor distribution constitute a major hurdle for the development of selective ligands and therapeutics [55], [56]. Low receptor correlation between mammalian species complicates the situation further and several compounds selective in rat or mice turn out to be unspecific at the human receptors restricting translation into the clinic significantly [57]. Nevertheless, OT is still the ligand of choice in the clinic and for most OTR studies, although it is well established that OT also signals *via* the AVP receptors [58]. A selective OT/AVP ligand has hence enormous potential for therapeutic development and it is intriguing to synthesize and analyze the novel ant inotocin peptides (and their modifications) for selectivity and potency on the human receptors in the future.

A similar peptide discovery approach from nature has already been successfully reported for the AVP-like conopressins [59]. Their discovery and characterization of conopressin-T in comparison with the human OT and AVP led to the identification of an agonist/antagonist switch, which is currently investigated towards antagonist design for the human receptors [59]. Besides future opportunities for investigating the structure-activity relationship of ant inotocin peptides and other neuropeptides (Table 1, Tables S1 and S2) similar strategies could be applied to defensins and other defense peptides as novel antimicrobial and cytolytic agents.

### Conclusion

Genome-mining can be considered as an efficient alternative to peptidomics analysis for the discovery of defense- and neuropeptide genes, in particular when the peptide sample amount is limited or difficult to obtain. Although the approach lacks to provide conclusive evidence for the biosynthesis of the peptides or information on post-translational modifications, the genetic information can be utilized to analyze the putative peptide sequences in molecular detail. That this can yield therapeutic drug leads was shown with conotoxin Vc1.1, which was identified *via* DNA sequencing, where surprisingly only the non-modified sequence and not the native, expressed and post-translational modified sequences was active in chronic pain models [60], [61], [62].

In this work we were able to annotate, analyze and discover encoding genes for many antimicrobial defense peptides, in particular ant defensins, and regulatory neuropeptides, such as allatostatin and tachykinin peptides and many others in three ant species *A.cephalotes* (leaf-cutter ant), *C.floridanus* (carpenter ant) and *H.saltator* (basal genus). It was also possible to identify and analyze OT/AVP-related peptides, so-called inotocins, and their putative receptors in social ants. Peptide sequences identified from nature should provide evolutionary advantage over random chemical libraries and future structure-activity relationship studies will show if some of these sequences can provide novel lead compounds for therapeutic drug design.

## Analysis

### Whole genome sequence data sets and GenBank accession numbers

The ant whole genome shotgun (WGS) sequences from *A.cephalotes* Ac (ADTU00000000) [17], *C.floridanus* Cf (AEAB00000000) [16] and *H.saltator* Hs (AEAC00000000) [16] were accessed via GenBank [63]. The following GenBank entries have been used for DNA annotation of novel coding sequences: Cf_pilosulin-like (AEAB00001185.1), Cf_defensin-1 (AEAB01018225.1), Cf_defensin-2 (AEAB01026853.1), Ac_defensin (ADTU01021145.1), Hs_defensin-1 (AEAC01007503.1), Hs_defensin-1a (AEAC01007503.1), Hs_defensin-2 (AEAC01015843.1), Cf_diuretic-hormone-like (AEAB01029697.1), Ac_diuretic-hormone-like (ADTU01016305.1), Hs_diuretic-hormone-like (AEAC01024560.1), Cf_abaecin-like (AEAB01017814.1), Ac_abaecin-like (ADTU01009315.1), Hs_abaecin-like (AEAC01003437.1), Ac_eclosion-hormone-like (ADTU01024031.1), Ac_neuroparsin-like (ADTU01005355.1), Cf_allatostatin (AEAB01013854.1), Hs_allatostatin (AEAC01017332.1), Ac_allatostatin (ADTU01011027.1), Hs_inotocin-vasopressin (AEAC01019310.1), Cf_inotocin-vasopressin-like (AEAB01028364.1), Ac_inotocin-vasopressin-like (ADTU01000445.1), Cf_ion-transport-peptide-like (AEAB01021789.1), Cf_CHH-like (AEAB01021790.1), Ac_ion-transport-peptide-like (ADTU01018997.1), Hs_ion-transport-peptidee (AEAC01008001.1) and Ac_tachykinin-related-peptide (ADTU01000331.1). The annotated sequences are presented in File S2. Nucleotide sequence data reported are available in the Third Party Annotation Section of the DDBJ/EMBL/GenBank databases under the accession numbers TPA: BK008403-BK008420.

### Genome-mining using tBLASTn similarity search

Query peptide sequences from related insect species were used for initial tBLASTn search [64] of WGS entries via GenBank (blosum62 matrix). Similar DNA (genomic) hits from the three ant species were collected and translated into their respective amino acid sequence (six-frame translation). The open reading frames were identified via manual sequence assignments. The identified amino acid sequences were confirmed by another tBLASTn alignment against the WGS database of each species and manual refinement. Final peptide sequences were collected and used for further identification and annotation.

### Identification and annotation of novel peptide genes

Annotation of tBLASTn genome hits was performed using the GeneWise2 algorithm [65] and manual refinement when necessary. The automated database query yielded full- or partial peptide sequences, their open reading frames and the corresponding DNA sequences (see File S3). The following settings were used: version 2.1.20, algorithm 6∶23, matrix blosum62. The predicted protein coding sequence as well as the intron/exon-structure from genomic data (without considering 5′ and 3′ untranslated regions) was used for further annotation and similarity alignments.

### Similarity alignments and feature annotation of peptide sequences

The web tool ClustalW2 was used to generate sequence alignments for comparison of novel ant peptides and precursor protein sequences to known and related UniProtKB entries. The position of signal peptide cleavage sites, pro- and prepro-protein regions as well as the mature peptide chains were defined by similarity with related proteins using the alignments. The sequence alignment graphs were prepared using the Boxshade 3.21 server.

### Peptide structure modeling

The model of *A.cephalotes* defensin is based on homology to the insect defensin phormicin (pdb code 1ICA, [27]) and the synthetic defensin analogue Def-BBB (pdb code 2E3E, [28]). The software modeller 9v9 was used to create sequence/structure alignments and energy minimization on distance restraints; 100 models were prepared and the most energetically favorable (according to its DOPE score) were selected. PyMol was used for graphical illustration of the structure model and distance measurement between negatively- and positively-charged side chains of the lowest energy model.

## Supporting Information

Figure S1 **Ant abaecin- and pilosulin-like defense peptides.** Similarity alignment of novel ant (**A**) abaecin-like (ABA) and (**B**) pilosulin-like (PIL) defense peptides with known insect peptides (UniProtKB C7AHU0, P15450, F4WAL0, Q26464, Q68Y23, Q68Y22 and A9CM07). The alignments were prepared with ClustalW2 and Boxshade. The mature peptides are presented in the box and the signal peptide cleavage site (identified by similarity) is indicated by an arrow.
(PDF)

Figure S2 **Alignment and evolutionary relationship of novel ant allatostatin peptides.** (**A**) Identified ant allatostatin (AST) precursor sequences from *Atta cephalotes*, *Camponotus floridanus* and *Harpegnathos saltator* were used for similarity alignment (ClustalW2) and compared to known allatostatins from *Apis mellifera* (UniProtKB P85797), and *Acromyrmex echinatior* (F4X8T3). The signal peptide cleavage site (identified by similarity) is shown as arrow. Mature allatostatin peptides are indicated in the boxes and are numbered by similarity to the *A.mellifera* precursor. The sequence alignment was prepared using Boxshade. (**B**) Gene structure of novel ant was predicted with the GeneWise algorithm and is presented in comparison to the *Drosophila melanogaster* drostatin (allatostatin homolog, GenBank NT033777.2) and *A.mellifera* (NC007084.3) precursor genes. Signal sequences are indicated in light grey, propeptide-regions in white and the mature peptide domains in dark grey. Intron sequences (including their base pair length) are indicated with upside-down arrow heads.
(PDF)

Figure S3 **Ant diuretic hormone-like and ion-transport-like/CHH-like peptides.** Similarity alignment of novel ant (**A**) diuretic hormone-like (DH) and (**B**) ion-transport-like peptides (ITP) with known insect peptides (UniProtKB Q9VLK4, F4X307, F4WGA3, Q1XAU6, Q1XAU8, Q9NL55, F4WAC6, Q26491 and Q1XAU8). The alignments were prepared with ClustalW2 and Boxshade. The mature peptides are presented by the box.
(PDF)

Figure S4 **Ant neuroparsin- and eclosion hormone-like peptides.** Similarity alignments of novel ant (**A**) neuroparsin-like (NP) and (**B**) eclosion hormone-like (EH) peptides with known insect peptides (UniProtKB Q07892, F4WBP0 and P10776). The alignments were prepared with ClustalW2 and Boxshade. The signal peptide cleavage site (identified by similarity) is indicated by an arrow.
(PDF)

Figure S5 **Ant tachykinin-related peptides.** Similarity alignments of novel ant (**A**) tachykinin-like peptides with known insect peptides (UniProtKB Q868G6, Q9VGE8 and F4WJJ0). The alignment was prepared with ClustalW2 and Boxshade. (**B**) The mature tachykinin-related peptide (TRP) sequences from *Atta cephalotes* are listed in comparison with *Drosophila melanogaster* TRPs. (**C**) Known human (*Homo sapiens*) tachykinin peptides contain the common F(x)GLM-NH_2_ motif with C-terminal amidation. Exemplarily neurokinin-A and B, endokinin A/B and C, substance P and neuropeptide K and γ are shown with their respective amino acid sequence.
(PDF)

Table S1 **tBLASTn summary of defense peptides and peptide toxins.**
(PDF)

Table S2 **tBLASTn summary of neuropeptides and regulatory peptide hormones.**
(PDF)

File S1 **tBLASTn results of (putative) ant oxytocin/vasopressin, allatostatin and tachykinin receptors.**
(PDF)

File S2 **Annotated DNA and translated (putative) precursor proteins from ants.**
(PDF)

File S3 **GeneWise raw data for prediction of gene structure.**
(PDF)
