# Supplementary figures and images for: Discovery of Defense- and Neuropeptides in Social Ants by Genome-Mining
Source: PLoS One. 2012 Mar 20;7(3):e32559. doi: 10.1371/journal.pone.0032559 (PMC3308954; doi:10.1371/journal.pone.0032559)

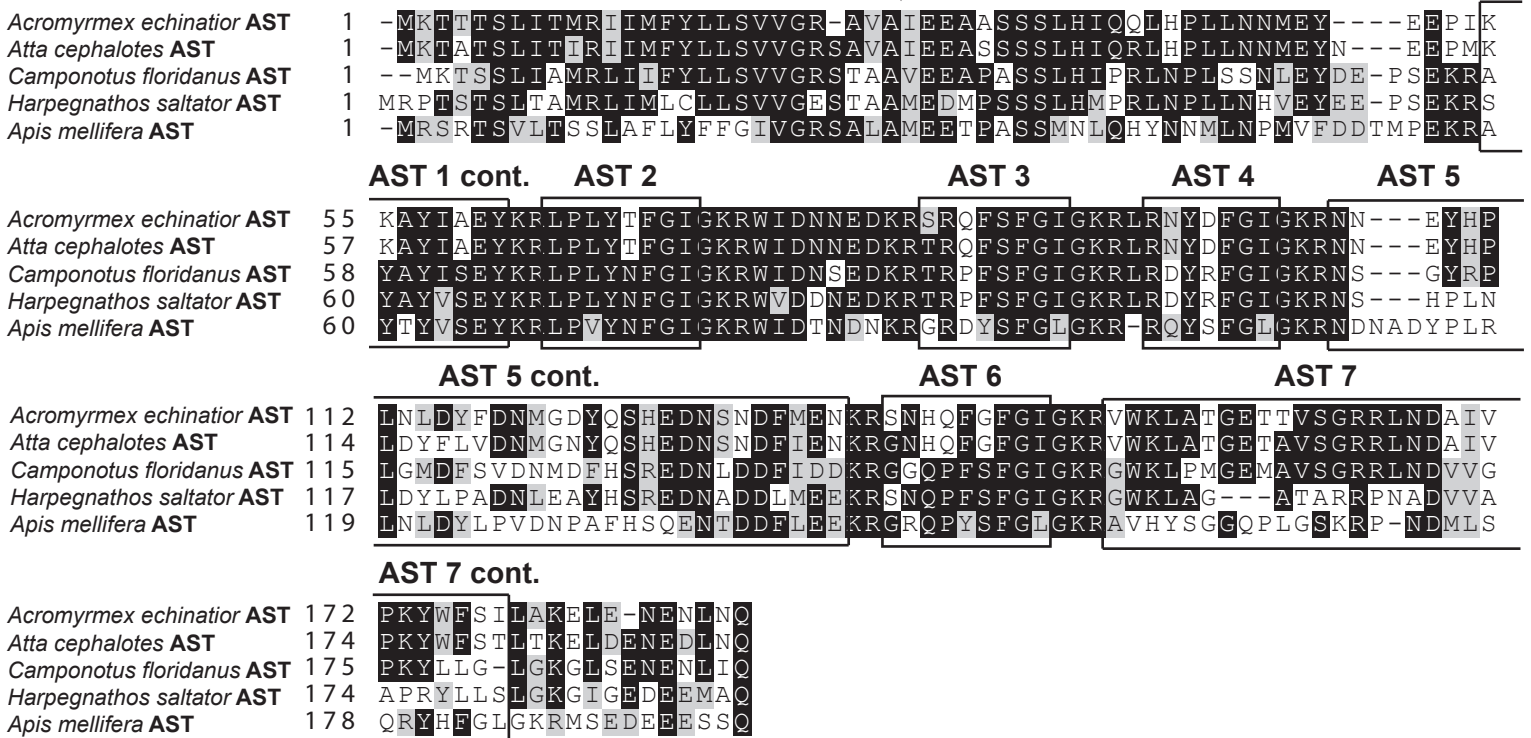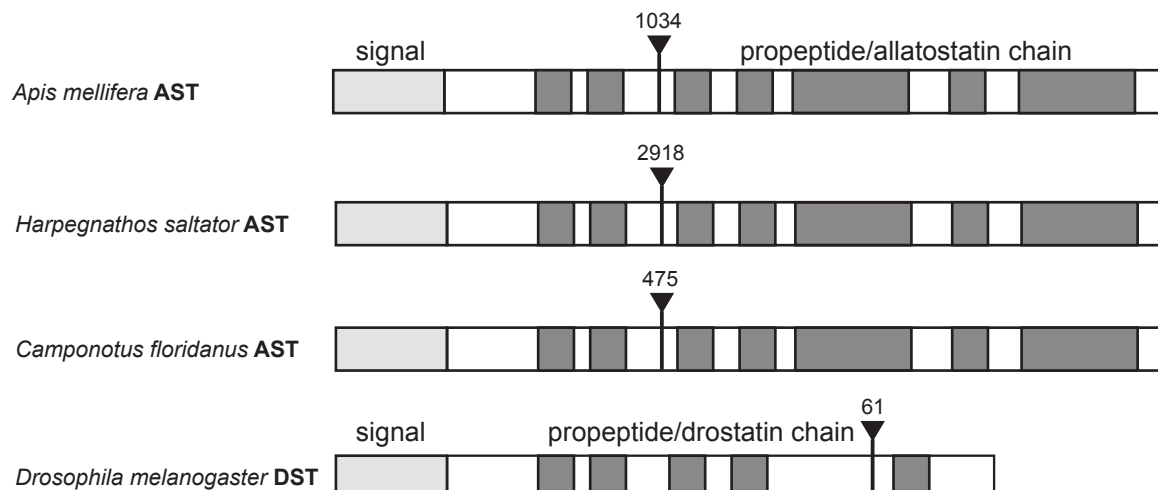

Supplement: Figure S2 — Alignment and evolutionary relationship of novel ant allatostatin peptides. (A) Identified ant allatostatin (AST) precursor sequences from Atta cephalotes, Camponotus floridanus and Harpegnathos saltator were used for similarity alignment (ClustalW2) and compared to known allatostatins from Apis mellifera (UniProtKB P85797), and Acromyrmex echinatior (F4X8T3). The signal peptide cleavage site (identified by similarity) is shown as arrow. Mature allatostatin peptides are indicated in the boxes and are numbered by similarity to the A.mellifera precursor. The sequence alignment was prepared using Boxshade. (B) Gene structure of novel ant was predicted with the GeneWise algorithm and is presented in comparison to the Drosophila melanogaster drostatin (allatostatin homolog, GenBank NT033777.2) and A.mellifera (NC007084.3) precursor genes. Signal sequences are indicated in light grey, propeptide-regions in white and the mature peptide domains in dark grey. Intron sequences (including their base pair length) are indicated with upside-down arrow heads. (PDF) [file pone.0032559.s002.pdf]
