# Supplementary material for: Discovery of Defense- and Neuropeptides in Social Ants by Genome-Mining
Source: PLoS One. 2012 Mar 20;7(3):e32559. doi: 10.1371/journal.pone.0032559 (PMC3308954; doi:10.1371/journal.pone.0032559)
Supplement: Figure S3 — Ant diuretic hormone-like and ion-transport-like/CHH-like peptides. Similarity alignment of novel ant (A) diuretic hormone-like (DH) and (B) ion-transport-like peptides (ITP) with known insect peptides (UniProtKB Q9VLK4, F4X307, F4WGA3, Q1XAU6, Q1XAU8, Q9NL55, F4WAC6, Q26491 and Q1XAU8). The alignments were prepared with ClustalW2 and Boxshade. The mature peptides are presented by the box. (PDF) [file pone.0032559.s003.pdf]

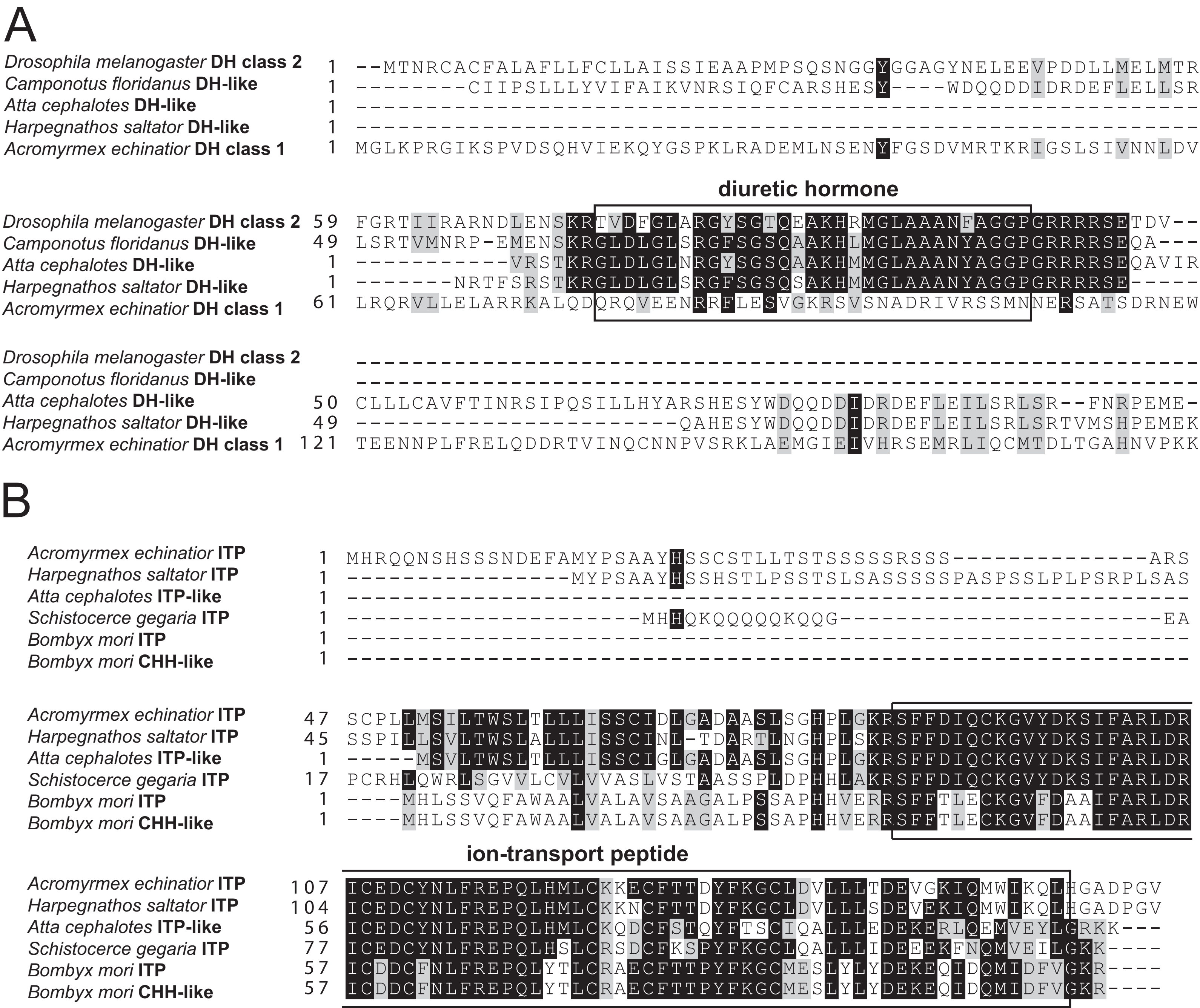
A
Drosophila melanogaster DH class 2 1 --MTNRCACFALAFLLFCLLAISSIEAAPMPSQSNGGYGGAGYNELEEVPDDLLMELMTR
Camponotus floridanus DH-like 1 --------CIIPSLLLYVIFAIKVNRSIQFCARSHESY----WDQQDDIDRDEFLELLSR
Atta cephalotes DH-like 1 --
Harpegnathos saltator DH-like 1 --
Acromyrmex echinatior DH class 1 1 MGLKPRGIKSPVDSQHVIEKQYGSPKLRADEMLNSENYFGSDVMRTKRIGSLSIVNNLDV
diuretic hormone
Drosophila melanogaster DH class 2 59 FGRTIIRARNDLENSKRTVDFGLARGYSGTQEAKHRMGLAAANFAGGPGRRRRSETDV--
Camponotus floridanus DH-like 49 LSRTVMNRP-EMENSKRGLDLGLSRGFSGSQAAKHLMGLAAANYAGGPGRRRRSEQA---
Atta cephalotes DH-like 1 -----------VRSTKRGLDLGLNRGYSGSQAAKHMMGLAAANYAGGPGRRRRSEQAVIR
Harpegnathos saltator DH-like 1 -------NRTFSRSTKRGLDLGLSRGFSGSQSAKHMMGLAAANYAGGPGRRRRSE-----
Acromyrmex echinatior DH class 1 61 LRQRVLLELARRKALQDQRQVEENRRFLESVGKRSVSNADRIVRSSMNNERSATSDRNEW
Drosophila melanogaster DH class 2 --
Camponotus floridanus DH-like --
Atta cephalotes DH-like 50 CLLLCAVFTINRSIPQSILLHYARSHESYWDQQDDIDRDEFLEILSRLSR--FNRPEME-
Harpegnathos saltator DH-like 49 ---------------------QAHESYWDQQDDIDRDEFLEILSRLSRTVMSHPEMEK
Acromyrmex echinatior DH class 1 121 TEENNPLFRELQDDRTVINQCNNPVSRKLAEMGIEIVHRSEMRLIQCMTDLTGAHNVPKK
B
Acromyrmex echinatior ITP 1 MHRQQNSHSSSNDEFAMYPSAAYHSSCSTLLTSTSSSSSRSSS---------------ARS
Harpegnathos saltator ITP 1 -----------------MYPSAAYHSSHSTLPSSTSLSASSSSSPASPSSLPLPSRPLSAS
Atta cephalotes ITP-like 1 --
Schistocerce gegaria ITP 1 ---------------------MHHQKQQQQKQQG----------------------EA
Bombyx mori ITP 1 --
Bombyx mori CHH-like 1 --
Acromyrmex echinatior ITP 47 SCPLLMSILTWSLTLLLISSCIDLGADAASLSGHPLGKRSFFDIQCKGVYDKSIFARLDR
Harpegnathos saltator ITP 45 SSPILLSVLTWSLALLLISSCINL-TDARTLNGHPLSKRSFFDIQCKGVYDKSIFARLDR
Atta cephalotes ITP-like 1 -----MSVLTWSLTLLLISSCIGLGADAASLSGHPLGKRSFFDIQCKGVYDKSIFARLDR
Schistocerce gegaria ITP 17 PCRHLQWRLSGVVLCVLVVASLVSTAASSPLDPHHLAKRSFFDIQCKGVYDKSIFARLDR
Bombyx mori ITP 1 ----MHLSSVQFAWAALVALAVSAAGALPSSAPHHVERRSFFTLECKGVFDAAIFARLDR
Bombyx mori CHH-like 1 ----MHLSSVQFAWAALVALAVSAAGALPSSAPHHVERRSFFTLECKGVFDAAIFARLDR
ion-transport peptide
Acromyrmex echinatior ITP 107 ICEDCYNLFREPQLHMLCKKECFTTDYFKGCLDVLLLTDEVGKIQMWIKQLHGADPGV
Harpegnathos saltator ITP 104 ICEDCYNLFREPQLHMLCKKNCFTTDYFKGCLDVLLLSDEVEKIQMWIKQLHGADPGV
Atta cephalotes ITP-like 56 ICEDCYNLFREPQLHMLCKQDCFSTQYFTSCIQALLLEDEKERLQEMVEYLGRKK---
Schistocerce gegaria ITP 77 ICEDCYNLFREPQLHSLCRSDCFKSPYFKGCLQALLLIDEEEKFNQMVEILGKK----
Bombyx mori ITP 57 ICDDCFNLFREPQLYTLCRAECFTTPYFKGCMESLYLYDEKEQIDQMIDFVGKR----
Bombyx mori CHH-like 57 ICDDCFNLFREPQLYTLCRAECFTTPYFKGCMESLYLYDEKEQIDQMIDFVGKR----
