# Supplementary material for: Discovery of Defense- and Neuropeptides in Social Ants by Genome-Mining
Source: PLoS One. 2012 Mar 20;7(3):e32559. doi: 10.1371/journal.pone.0032559 (PMC3308954; doi:10.1371/journal.pone.0032559)
Supplement: Figure S4 — Ant neuroparsin- and eclosion hormone-like peptides. Similarity alignments of novel ant (A) neuroparsin-like (NP) and (B) eclosion hormone-like (EH) peptides with known insect peptides (UniProtKB Q07892, F4WBP0 and P10776). The alignments were prepared with ClustalW2 and Boxshade. The signal peptide cleavage site (identified by similarity) is indicated by an arrow. (PDF) [file pone.0032559.s004.pdf]

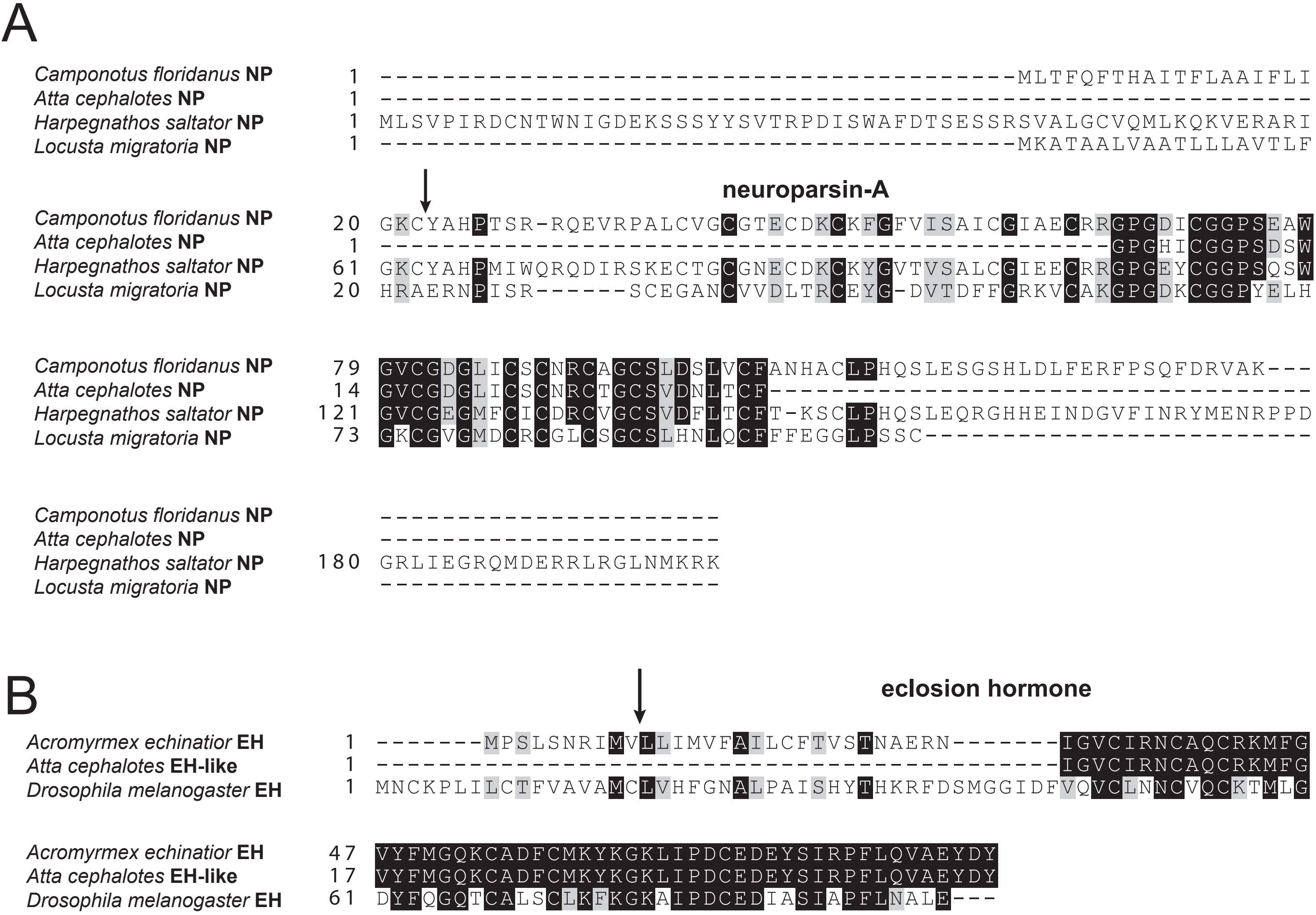
A
neuroparsin-A
Camponotus floridanus NP 1 ---------------------------------------MLTFQFTHAITFLAAIFLI
Atta cephalotes NP 1 --
Harpegnathos saltator NP 1 MLSVPIRDCNTWNIGDEKSSSYYSVTRPDISWAFDTSESSRSVALGCVQMLKQKVERARI
Locusta migratoria NP 1 ---------------------------------------MKATAALVAATLLLAVTLF
Camponotus floridanus NP 20 GKCYAHPTSR-RQEVRPALCVGCGTECDKCKFGFVISAICGIAECRRGPGDICGGPSEAW
Atta cephalotes NP 1 ---GPGHICGGPSDSW
Harpegnathos saltator NP 61 GKCYAHPMIWQRQDIRSKECTGCGNECDKCKYGVTVSALCGIEECRRGPGEYCGGPSQSW
Locusta migratoria NP 20 HRAERNPISR------SCEGANCVVDLTRCEYG-DVTDFFGRKVCAKGPGDKCGGPYELH
Camponotus floridanus NP 79 GVCGDGLICSCNRCAGCSLDSLVCFANHACLPHQSLESGSHLDLFERFPSQFDRVAK---
Atta cephalotes NP 14 GVCGDGLICSCNRCTGCSVDNLTCF-----------------------------------
Harpegnathos saltator NP 121 GVCGEGMFCICDRCVGCSVDFLTCFT-KSCLPHQSLEQRGHHEINDGVFINRYMENRPPD
Locusta migratoria NP 73 GKCGVGMDCRCGLCSGCSLHNLQCFFFEGGLPSSC-------------------------
Camponotus floridanus NP ----------------------
Atta cephalotes NP ----------------------
Harpegnathos saltator NP 180 GRLIEGRQMDERRLRGLNMKRK
Locusta migratoria NP ----------------------
B
eclosion hormone
Acromyrmex echinatior EH 1 -------MPSLSNRIMVLLIMVFAILCFTVSTNAERN-------IGVCIRNCAQCRKMFG
Atta cephalotes EH-like 1 --IGVCIRNCAQCRKMFG
Drosophila melanogaster EH 1 MNCKPLILCTFVAVAMCLVHFGNALPAISHYTHKRFDSMGGIDFVQVCLNNCVQCKTMLG
Acromyrmex echinatior EH 47 VYFMGQKCADFCMKYKGKLIPDCEDEYSIRPFLQVAEYDY
Atta cephalotes EH-like 17 VYFMGQKCADFCMKYKGKLIPDCEDEYSIRPFLQVAEYDY
Drosophila melanogaster EH 61 DYFQGQTCALSCLKFKGKAIPDCEDIASIAPFLNALE---
