# Supplementary material for: Discovery of Defense- and Neuropeptides in Social Ants by Genome-Mining
Source: PLoS One. 2012 Mar 20;7(3):e32559. doi: 10.1371/journal.pone.0032559 (PMC3308954; doi:10.1371/journal.pone.0032559)
Supplement: Figure S5 — Ant tachykinin-related peptides. Similarity alignments of novel ant (A) tachykinin-like peptides with known insect peptides (UniProtKB Q868G6, Q9VGE8 and F4WJJ0). The alignment was prepared with ClustalW2 and Boxshade. (B) The mature tachykinin-related peptide (TRP) sequences from Atta cephalotes are listed in comparison with Drosophila melanogaster TRPs. (C) Known human (Homo sapiens) tachykinin peptides contain the common F(x)GLM-NH2 motif with C-terminal amidation. Exemplarily neurokinin-A and B, endokinin A/B and C, substance P and neuropeptide K and γ are shown with their respective amino acid sequence. (PDF) [file pone.0032559.s005.pdf]

A

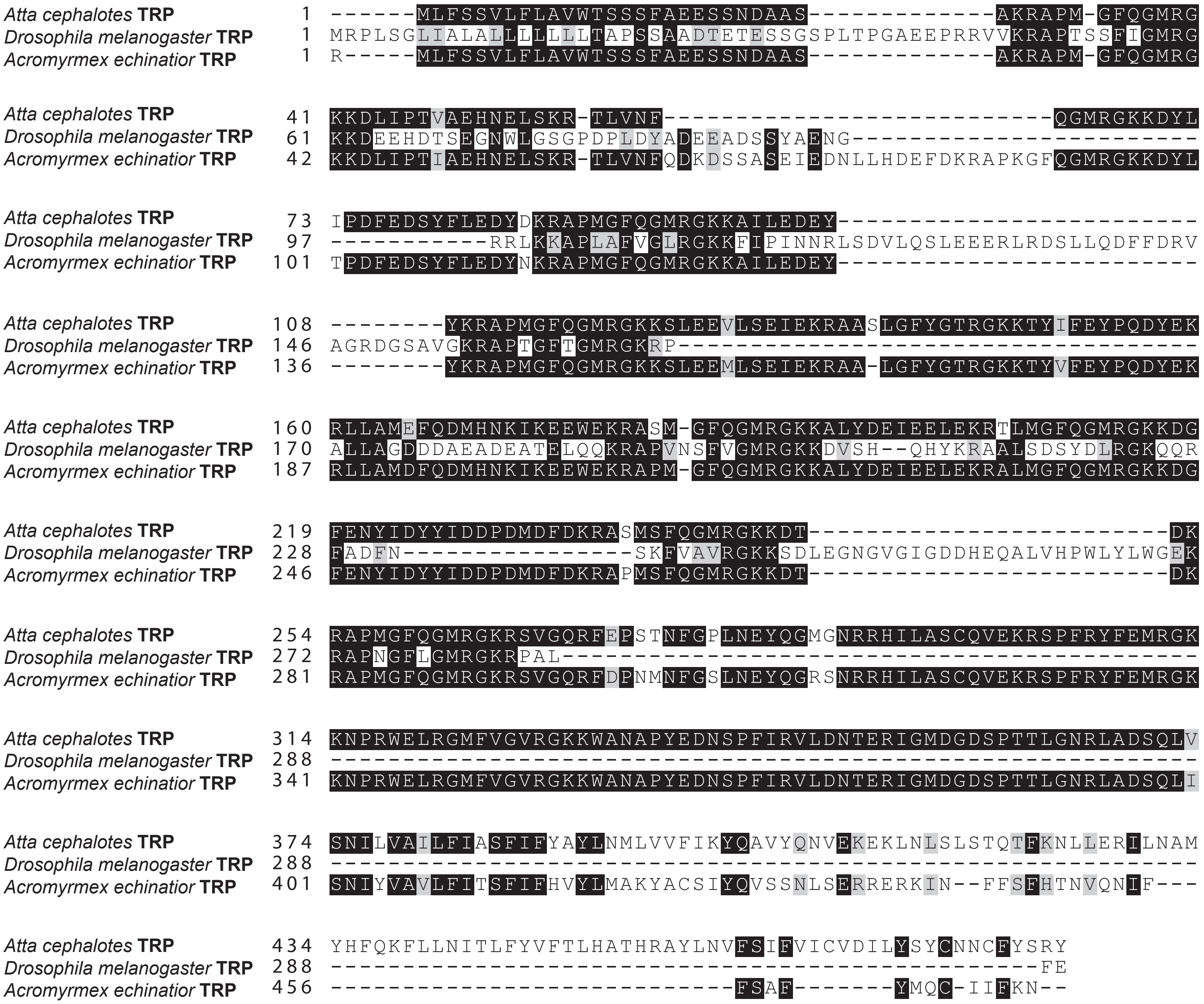

B

	Tachykinin-related peptide 1, TRP-1	TRP-2	TRP-3	TRP-4	TRP-5
D. melanogaster	APTSSFIGMR-NH_2	APLAFVGLR-NH_2	APTGFTGMR-NH_2	APVNSFVGMR-NH_2	APNGFLGMR-NH_2
A. cephalotes	APM-GFQGMR-NH_2	APMGFQGMR-NH_2	APMGFQGMR-NH_2	APM-GFQGMR-NH_2	APMGFQGMR-NH_2
A. cephalotes	TRP-1a<br>TLVNFQGMR-NH_2			TRP-4a<br>TLMGFQGMR-NH_2<br>TRP-4b<br>ASMSFQGMR-NH_2	

C

F(x)GLM-NH_2 motif

Homo sapiens	Neurokinin A	DMHDFFVGLM-NH_2
	Neurokinin B	HKTDSFVGLM-NH_2
	Endokinin A/B	GKASQFFGLM-NH_2
	Endokinin C	KKAYQLEHTFQGLL-NH_2
	Substance P	RPKPQQFFGLM-NH_2
	Neuropeptide K	DADSSIEKQVALLKALYGHGQISHKRHKTDSFVGLM-NH_2
	Neuropeptide γ	DAGHGQISHKRHKTDSFVGLM-NH_2
