# Supplementary material for: Discovery of Defense- and Neuropeptides in Social Ants by Genome-Mining
Source: PLoS One. 2012 Mar 20;7(3):e32559. doi: 10.1371/journal.pone.0032559 (PMC3308954; doi:10.1371/journal.pone.0032559)
Supplement: Table S1 — tBLASTn summary of defense peptides and peptide toxins. (PDF) [file pone.0032559.s006.pdf]

Table S1. tBLASTn summary of defense peptides and peptide toxins

**Peptide class**	**Query sequences**		**tBLASTn results (GenBank WGS accessions)***		
	Name	UniProtKB/ GenBank ID	*A.cephalotes*	*C.floridanus*	*H.saltator*
**Ant defensins**					
	Defensin (*Myrmica ruginodis*)	tr|C7AHT2		gb|AEAB01026853.1	
	Defensin (*M.ruginodis*)	tr|B9TXS6		gb|AEAB01026853.1	
	Defensin (*Formica aquilonia*)	sp|Q5BU36	gb|ADTU01021145.1	gb|AEAB01026853.1	gb|AEAC01007503.1
	Defensin (*Lasius niger*)	tr|B9TXS0	gb|ADTU01021145.1	gb|AEAB01026853.1	gb|AEAC01007503.1
**Other ant defense and antimicrobial peptides**					
	Abaecin (*M.ruginodis*)	tr|C7AHU0	gb|ADTU01009315.1	*see Table 1*	*see Table 1*
	Dinoponeratoxin (*Dinoponera australis*)	sp|P0CF01			
	Dinoponeratoxin (*D.australis*)	sp|P0CF02			
	Ectatomin subunit A (*Ectatomma tuberculatum*)	sp|P49343			
	Ectatomin subunit B (*E.tuberculatum*)	sp|P49344			
	Formaecin-1 (*M.gulosa*)	sp|P81438			
	Formaecin-2 (*M.gulosa*)	sp|P81437			
	Pilosulin-1 (*Myrmecia pilosula*)	sp|Q07932			
	Pilosulin-2 (*M.pilosula*)	sp|Q26464		gb|AEAB01001185.1	
	Pilosulin-3 (*M.banksi*)	sp|Q68Y23		gb|AEAB01001185.1	
	Pilosulin-3a (*M.pilosula*)	sp|Q26464			
	Pilosulin-3b (*M.pilosula*)	sp|P0C023			
	Pilosulin-4 (*M.banksi*)	sp|Q68Y22			
	Pilosulin 5 (*M.banksi*)	tr|A9CM07			
	Poneratoxin (*Paraponera clavata*)	sp|P41736			
	Ponericin-G1 (*Pachycondyla goeldii*)	sp|P82414			
	Ponericin-W3 (*P.goeldii*)	sp|P82425			
	Queen venom protein Sol g II (*Solenopsis geminata*)	tr|B1WA75			
**Insect defensins**					
	Defensin-1 (*Apis mellifera*)	sp|P17722	gb|ADTU01021145.1	gb|AEAB01026853.1<br>gb|AEAB01018225.1	gb|AEAC01007503.1<br>gb|AEAC01015843.1
	Defensin-2 (*A.mellifera*)	sp|Q5MQL3	gb|ADTU01021145.1	gb|AEAB01018225.1<br>gb|AEAB01026853.1	gb|AEAC01007503.1
	Defensin (*Drosophila melanogaster*)	sp|P36192	gb|ADTU01021145.1	gb|AEAB01026853.1	
	Drosomycin (*D.melanogaster*)	sp|P41964			

**Insect defense peptides and peptide toxins**			
	Apamin protein (*A.mellifera*)	tr|B7UUK0	
	Apidaecin (*A.cerana cerana*)	gb|ACJ22838.1	gb|ADTU01018408.1 gb|AEAB01001738.1 gb|AEAC01018260.1 gb|ADTU01036819.1
	Hymenoptaecin (*A.mellifera*)	gb|AAA67444.1	gb|ADTU01018408.1 gb|AEAB01001738.1 gb|AEAC01018260.1 gb|ADTU01036819.1
	Hymenoptaecin (*A.cerana cerana*)	gb|ACH96430.1	gb|ADTU01018408.1 gb|AEAB01001738.1 gb|AEAC01018260.1 gb|ADTU01036819.1
	Melittin (*A. mellifera*)	sp|P01501	
	Prepromastoparan-B (*Vespa basalis*)	tr|Q0R4F6	
**Plant defense peptides**			
	Defensin-like-protein (*Arabidopsis thaliana*)	sp|Q9ZUL7	
	Kalata-B1 (*Oldenlandia affinis*)	sp|P56254	
	Tricyclon A protein (*Viola tricolor*)	tr|B6E617	
**Spider peptide toxins**			
	Gomesin (*Acanthoscurria gomesiana*)	sp|P82358	
	Omega-ctenitoxin-Cs1a (*Cupiennius salei*)	sp|P81694	
**Frog antimicrobial peptides**			
	Gaegurin-4 (*Glandirana rugosa*)	sp|P80398	
	Magainins (*Xenopus laevis*)	sp|P11006	
	Sauvagin (*Phyllomedusa sauvagei*)	sp|P01144	
**Snake peptide toxins**			
	Long-neurotoxin-1 (*Notechis scutatus scutatus*)	sp|P01384	
	Short-neurotoxin-1 (*Pseudechis australis*)	sp|P25497	
**Cone-snail toxins**			
	Alpha-conotoxin-MII (*C.magus*)	sp|P56636	
	Omega-conotoxin-MVIIA (*Conus magus*)	sp|P05484	
**Human defensins**			
	Defensin-alpha-1 (*Homo sapiens*)	tr|Q6EZF6	
	Defensin- beta-1 (*H.sapiens*)	sp|P60022	

*ant genome WGS database hits of selected peptides were used for further tBLASTn and genome-mining approach as described in the manuscript; color coding: green = putative peptides found in all three ant species, yellow = putative peptides found in at least one species, red = no peptides found in *A.cephalotes*, *C .floridanus*, *H.saltator* using tBLASTn;
