# Supplementary material for: Discovery of Defense- and Neuropeptides in Social Ants by Genome-Mining
Source: PLoS One. 2012 Mar 20;7(3):e32559. doi: 10.1371/journal.pone.0032559 (PMC3308954; doi:10.1371/journal.pone.0032559)
Supplement: Table S2 — tBLASTn summary of neuropeptides and regulatory peptide hormones. (PDF) [file pone.0032559.s007.pdf]

Table S2. tBLASTn summary of neuropeptides and regulatory peptide hormones

Peptide class	Query sequences		tBLASTn results (GenBank WGS accessions)*		
	Name	UniProtKB/ GenBank ID	*A.cephalotes*	*C.floridanus*	*H.saltator*
**Neuropeptides/GPCR ligands**					
	Adipokinetic hormone (= gonadotropin-releasing hormone) (*Drosophila melanogaster*)	sp|P61855			
	Adipokinetic prohormone type 2 (*Schistocerca nitens*)	sp|P35807			
	Adipokinetic hormone 1 (*Tribolium castaneum*)	tr|A3RE76			
	Adipokinetic hormone 2 (*T.castaneum*)	tr|D6WNV7			
	Allatostatin (*Acromyrmex echinatior*)[+]	tr|F4X8T3	gb|ADTU01011027.1	gb|AEAB01013854.1	gb|AEAC01017332.1
	Allatostatins (*Apis mellifera*)[+]	sp|P85797	gb|ADTU01011027.1	gb|AEAB01013854.1	gb|AEAC01017332.1
	Allatotropin (*T.castaneum*)	tr|D6WWW4			
	Arginine-vasopressin-like-propeptide (*Nasonia vitripennis*)[+]	XP001606547.1	gb|ADTU01000445.1	gb|AEAB01028362.1	gb|AEAC01019310.1
	Arginine-vasopressin-like-propeptide (*T.castaneum*)[+]	tr|A3RE83	gb|ADTU01000445.1	gb|AEAB01028362.1	gb|AEAC01019310.1
	Cardioactive peptide (*D.melanogaster*)	sp|Q9VCW0			
	Cardioactive peptide (*M.sexta*)	sp|Q8WRC7			
	Cardio acceleratory peptide 2b (*D.melanogaster*)	sp|Q9NIP6			
	(Pro-)Corazonin (*Camponotus floridanus*)[§]	tr|E2ARW3	gb|ADTU01018055.1	*not applicable*[#]	gb|AEAC01003435.1
	(Pro-)Corazonin (*Harpegnathos saltator*)[§]	tr|E2B7L4	gb|ADTU01018055.1	gb|AEAB01023843.1	*n.a.*
	(Pro-)Corazonin (*A.mellifera*)	sp|Q5DW47	gb|ADTU01018055.1	gb|AEAB01023843.1	gb|AEAC01003435.1
	(Pro-)Corazonin (*D.melanogaster*)	sp|Q26377	gb|ADTU01018055.1		gb|AEAC01003435.1
	Ecdysis triggering hormone preprotein (*A.mellifera*)	tr|B6F2B8	gb|ADTU01012083.1	gb|AEAB01024202.1	gb|AEAC01018952.1
	Ecdysis triggering hormone (*D.melanogaster*)	tr|Q9W103	gb|ADTU01012083.1		gb|AEAC01018952.1
	Ecdysis triggering hormone (*T.castaneum*)	tr|D6WRE3			gb|AEAC01018952.1
	FMRFamide-related neuropeptides (*C.floridanus*)[§]	tr|E2A009		gb|AEAB01004040.1	
	FMRFamide (*A.mellifera*)	tr|F8J1L0	gb|ADTU01002885.1	gb|AEAB01003438.1	gb|AEAC01013831.1
	FMRFamide-related peptides (*Bombyx mori*)	sp|Q1MX22			
	FMRFamide-related peptides (*D.melanogaster*)	sp|P10552			
	FMRFamide-related (*T.castaneum*)	tr|D2A1T0			
	Myosuppressin (*A.mellifera*)	sp|P85527	gb|ADTU01018774.1	gb|AEAB01006496.1	gb|AEAC01020994.1
	(Dro-)Myosuppressin (*D.melanogaster*)	sp|P61849	gb|ADTU01018774.1	gb|AEAB01006496.1	gb|AEAC01020994.1
	Myosuppressin (*T.castaneum*)	tr|D7EIL9			
	Neuropeptide F (*D.melanogaster*)	sp|Q9VET0			
	Neuropeptide F (*Locusta migratoria*)	sp|P86442			

	Neuropeptide Y-like (*A.mellifera*) [+]	NP001161192.1	gb|ADTU01008737.1	gb|AEAB01013583.1	gb|AEAC01022580.1
	Orcokinin peptides (*C.floridanus*) [§]	tr|E1ZVK3	gb|ADTU01022510.1	*n.a.*	gb|AEAC01016540.1
	Orcokinin peptides (*H.saltator*) [§]	tr|E2BU65	gb|ADTU01022510.1	gb|AEAB01000665.1	*n.a.*
	Orcokinin peptides (*A.mellifera*)	sp|P85832	gb|ADTU01022510.1	gb|AEAB01000665.1	gb|AEAC01016540.1
	PBAN (=pheromone biosynthesis-activating)-type neuropeptide (*H.saltator*) [§]	tr|E2B2R9	gb|ADTU01002850.1 gb|ADTU01002851.1	gb|AEAB01014705.1	*n.a.*
	PBAN-type neuropeptide (*A.mellifera*)	sp|A8CL69	gb|ADTU01002850.1 gb|ADTU01002851.1	gb|AEAB01014705.1	gb|AEAC01000416.1
	Protein pigment dispersing factor (PDF) (*D.melanogaster*)	sp|O96690	gb|ADTU01028995.1	gb|AEAB01027821.1	gb|AEAC01018247.1
	Pyrokinin (*T.castaneum*)	tr|D6W7C9		gb|AEAB01014705.1	gb|AEAC01000416.1
	Short neuropeptide F (*D.melanogaster*)	sp|Q9VIQ0		gb|AEAB01017307.1	gb|AEAC01008675.1
	Short neuropeptide F (*T.castaneum*)	tr|D6X455	gb|ADTU01008389.1	gb|AEAB01017307.1	gb|AEAC01008675.1
	Pro-Sialokinin (*Aedes aegypti*)	sp|P42634			
	(Dro-)Sulfakinins (*D.melanogaster*)	sp|P09040	gb|ADTU01031336.1	gb|AEAB01014680.1	
	Sulfakinin (*T.castaneum*)	tr|D6WP08	gb|ADTU01031336.1	gb|AEAB01014680.1	gb|AEAC01011304.1
	Tachykinin (*A.echinatior*)	tr|F4WJJ0	gb|ADTU01000331.1	gb|AEAB01016992.1	no hits, see footnote[&]
	Tachykinins (*A.mellifera*)	sp|Q868G6	gb|ADTU01000331.1	gb|AEAB01016992.1	no hits, see footnote[&]
**Other neuropeptides**					
	Hypothetical protein LOC409634 (*A.mellifera*)	XP393134.4	gb|ADTU01027978.1	gb|AEAB01020462.1	gb|AEAC01010737.1
	Hypertrehalosaemic prohormone (*C.floridanus*)	tr|E2AMJ9	gb|ADTU01004554.1	*n.a.*	gb|AEAC01021474.1
	Nogo-B receptor-like peptide (*A.mellifera*)	XP001120453.2	gb|ADTU01027831.1	gb|AEAB01010219.1	gb|AEAC01010598.1
	Proctolin (*D.melanogaster*)	tr|Q9VLV9			
	Protein hugin (*D.melanogaster*)	sp|Q9VG55			
**Osmoregulatory peptides/GPCR ligands**					
	Diuretic hormone (*Acheta domesticus*)	sp|P23834			
	Putative diuretic hormone-I (*A.mellifera*)	tr|Q5F302	gb|ADTU01020665.1	gb|AEAB01008798.1	gb|AEAC01003516.1
	Diuretic hormone class 2 (*A.mellifera*)	sp|P85830	gb|ADTU01016305.1	gb|AEAB01029697.1	gb|AEAC01024560.1
	Putative diuretic hormone-I (*A.mellifera*)	tr|Q5F302	gb|ADTU01020665.1	gb|AEAB01008798.1	gb|AEAC01003516.1
	Diuretic-hormone-class2 (*D.melanogaster*)	sp|Q9VLK4	gb|ADTU01016305.1	gb|AEAB01029697.1	gb|AEAC01024560.1
	Diuretic hormone, isoform A (*D.melanogaster*)	tr|Q9VH98	gb|ADTU01020665.1	gb|AEAB01008798.1	gb|AEAC01003516.1
	Diuretic hormone, isoform B (*D.melanogaster*)	tr|A8JQW2	gb|ADTU01020665.1	gb|AEAB01008798.1	gb|AEAC01003516.1
	Diuretic hormone (*L.migratoria*)	sp|P23465			
	Diuretic hormone 37 like protein (*T.castaneum*)	tr|D7EJD2			
	Diuretic hormone 31 like protein (*T.castaneum*)	tr|D6WC08	gb|ADTU01016305.1	gb|AEAB01029697.1	gb|AEAC01024560.1
	Diuretic hormone 47 (*T.castaneum*)	tr|D7EJD3	gb|ADTU01020665.1	gb|AEAB01008798.1	gb|AEAC01003516.1
	CHH-like-protein (*B.mori*)	sp|Q9NL55	gb|ADTU01018997.1	gb|AEAB01021790.1	
	Ion-transport-peptide-like (*A.echinatior*)	tr|F4WAC6	gb|ADTU01018997.1	gb|AEAB01021789.1	gb|AEAC01008001.1

				gb|AEAB01021790.1	
	Ion-transport-peptide (*B.mori*)	tr|Q1XAU6	gb|ADTU01018997.1	gb|AEAB01021789.1	gb|AEAC01008001.1
	Ion-transport-peptide (*M.sexta*)	tr|Q1XAU8	gb|ADTU01018997.1	gb|AEAB01021789.1	gb|AEAC01008001.1
**Peptide hormones/ GPCR ligands**					
	Eclosion-hormone (*A.echinatior*)	tr|F4WBP0	gb|ADTU01024031.1	gb|AEAB01027960.1	gb|AEAC01015798.1
	Eclosion-hormone (*C.floridanus*) §	tr|E2AXD4	gb|ADTU01024031.1	*n.a.*	gb|AEAC01015798.1
	Eclosion-hormone (*H.saltator*) §	tr|E2BSX6	gb|ADTU01024031.1	gb|AEAB01027960.1	*n.a.*
	Eclosion-hormone (*D.melanogaster*)	sp|Q07892	gb|ADTU01024031.1	gb|AEAB01027960.1	gb|AEAC01015798.1
	Neuroparsin-A (*C.floridanus*) §	tr|E1ZXL4	gb|ADTU01005355.1	*n.a.*	gb|AEAC01011937.1
	Neuroparsin-A (*H.saltator*) §	tr|E2BLJ9	gb|ADTU01005355.1	gb|AEAB01002251.1	*n.a.*
	Neuroparsin-A (*L.migratoria*)	sp|P10776	gb|ADTU01005355.1	gb|AEAB01002251.1	gb|AEAC01011937.1
	Parathyroid hormone-related peptide (*A.mellifera*)	XP001122670.2	gb|ADTU01018725.1<br>gb|ADTU01018724.1<br>gb|ADTU01009906.1	gb|AEAB01003949.1<br>gb|AEAB01020496.1	gb|AEAC01011813.1
	Prohormone-1 (*A.mellifera*)	sp|P85798	gb|ADTU01027989.1	gb|AEAB01008908.1	gb|AEAC01000861.1
	Prohormone-2 (*A.mellifera*)	sp|P85799	gb|ADTU01018849.1	gb|AEAB01004019.1	gb|AEAC01018572.1
	Prohormone-3 (*A.mellifera*)	sp|P85828	gb|ADTU01008748.1	gb|AEAB01013608.1<br>gb|AEAB01013607.1	gb|AEAC01022581.1
	Prohormone-4 (*A.mellifera*)	sp|P85831	gb|ADTU01028453.1<br>gb|ADTU01027871.1	gb|AEAB01027841.1	gb|AEAC01017641.1
**Other regulatory peptides and peptide hormones**					
	Bombyxin (*A.mellifera*)	tr|D5L5R1	gb|ADTU01003244.1	gb|AEAB01028366.1<br>gb|AEAB01014084.1	gb|AEAC01019650.1
	Latrophilin-3 (*C.floridanus*) §	tr|E2A464	gb|ADTU01022358.1<br>gb|ADTU01028440.1<br>gb|ADTU01031332.1	*n.a.*	gb|AEAC01003294.1
	Prothoracicotropic hormone (*D.melanogaster*)	tr|Q9VPR8			
	Protein pigment dispersing factor (PDF) (*D.melanogaster*)	sp|O96690	gb|ADTU01028995.1	gb|AEAB01027821.1	gb|AEAC01018247.1
	Queen brain-selective protein-1 (*A.mellifera*)	tr|Q1T786	gb|ADTU01005355.1	gb|AEAB01002251.1	gb|AEAC01011937.1

[*]ant genome WGS database hits of selected peptides were used for further tBLASTn and genome-mining approach as described in the manuscript; color coding: green = putative peptides found in all three ant species, yellow = putative peptides found in at least one species, red = no peptides found in *A.cephalotes*, *C .floridanus*, *H.saltator* using tBLASTn; [#]not applicable, n.a. since the query protein was from this species; [§]UniProtKB entries of these peptides have been released during the preparation of the manuscript for reference see Bonasio *et al.* (2010) Science 329: 1068-1071; [&]During the preparation of this manuscript the receptor sequences for tachykinins were released on UniProtKB: *C.floridanus*: tr|E2AB29, tr|E2AB30, tr|E2ALS3, tr|E2ALS4; *H.saltator*: tr|E2B748, tr|E2B749; [+]GPCRs from *C.floridanus* and *H.saltator* have been annotated for these ligands, for reference see Bonasio *et al.* (2010)
