# Supplementary material for: Discovery of Defense- and Neuropeptides in Social Ants by Genome-Mining
Source: PLoS One. 2012 Mar 20;7(3):e32559. doi: 10.1371/journal.pone.0032559 (PMC3308954; doi:10.1371/journal.pone.0032559)
Supplement: File S1 — tBLASTn results of (putative) ant oxytocin/vasopressin, allatostatin and tachykinin receptors. (PDF) [file pone.0032559.s008.pdf]

tBLASTn results of (putative) ant oxytocin/vasopressin receptors

Query sequence:

```
>gi|145651804|ref|NP_001078830.1| inotocin receptor [Tribolium castaneum]
MYTPKLSQMDISENSTYLFDKHEDRNNTDRDENLARVEVATLAIIFLVTVIGNSTVLLALWTRRRYAGRK
KLSRMYFFILHLSIADLITAFLSVLPQLAWDITYRFYGGFLLCKVVKYGQTLGPYLSSYVLMATAIDRHQ
AICYPLTYCSWTSRRSKVMVYLAWVASLAFCIPQLTIFTYTSVGEDEYDCWATFQEPWGKRAYVTWYSIS
VFMVPLVVLIFTYTSICIEIWQSSESSLRPRSSQKSAPGKRTPLISRAKINTVKQTIAVIVMYIACSTPF
ILAQLWATWDPQSPFIDGPVFVILTLLYSLNSCVNPWIYLAFNRELPRLLLRHYTASSKNYRSATGGNSA
SNSSGDAQSTSLRPFSRWSLCNSARSNKYPTRVPHRPYVAQYNARRWIV
```

Hits in A.cephalotes WGS database:

```
gb|ADTU01023622.1| Atta cephalotes contig23622, whole genome shotgun sequence
Length=21064

 Score = 85.9 bits (211),  Expect = 2e-14, Method: Compositional matrix adjust.
 Identities = 44/134 (33%), Positives = 80/134 (60%), Gaps = 0/134 (0%)
 Frame = -3

Query  20    VEISTLSVILVLAVTSNLTMLIAIWRQRRNRPLSRMYFFMMHLSLADLLVALFNILPQLA  79
             +++  L++I VL++  NL  + +I + RR R  S +Y  + HLS+ADLLV +F +  +
Sbjct  2618  LKVIVLAIISVLSLVGNLATIYSITKNRRKRHGSTIYLLIFHLSVADLLVTIFCLAGEAI  2439

Query  80    WDITYRFQGGDVLCRFVKYAQVMTLYLSTYILMFMAVDRYRAVCCRNLHWNSLKVAKCFV  139
             W  T  +  G++ C+  K+ Q+ +LYLST++L+ + VDR+ AV       N+ +    FV
Sbjct  2438  WSYTVAWLWGNIACKIFKFLQMFSLYLSTFVLVLIGVDRFVAVRYPMKGLNTPQTCSRFV  2259

Query  140   AASWVMAILFSIPQ  153
               +W+++ + + PQ
Sbjct  2258  LLTWLLSFILATPQ  2217


gb|ADTU01011675.1|  Atta cephalotes contig11675, whole genome shotgun sequence
Length=5882

 Score = 85.1 bits (209),  Expect = 4e-14, Method: Compositional matrix adjust.
 Identities = 40/79 (51%), Positives = 57/79 (72%), Gaps = 2/79 (3%)
 Frame = -1

Query  9     SSSLRDEKLASVEISTLSVILVLAVTSNLTMLIAIWRQRR--NRPLSRMYFFMMHLSLAD  66
                 RDE LA  EI+ L+ I ++ +  N+ +L+A++ +RR   R  +RMYFF+MHLS+AD
Sbjct  1379  DDDARDEYLARWEIAVLTSIFLITIIGNVLVLLALYARRRYQRRKFTRMYFFIMHLSVAD  1200

Query  67    LLVALFNILPQLAWDITYR  85
             LL  LF++LPQLAWDIT+R
Sbjct  1199  LLTGLFDVLPQLAWDITFR  1143


gb|ADTU01017567.1|  Atta cephalotes contig17567, whole genome shotgun sequence
Length=6662

 Score = 73.2 bits (178),  Expect = 4e-10, Method: Compositional matrix adjust.
 Identities = 68/301 (23%), Positives = 136/301 (45%), Gaps = 43/301 (14%)
 Frame = +1

Query  26    SVILVLAVTSNLTMLIAIWRQRRNRPLSRMYFFMMHLSLAD--LLVALFNILPQLAWDIT  83
             S++ ++ +  N  ++   + R  + + ++ MY  +++L++AD   L+ +    P L   I+
Sbjct  3457  SIVCIVGLLGNTLVIYVVLRFAKMQTVTNMY--IVNLAIADECFLIGI----PFLVTTIS  3618

Query  84    YRFQG---GDVLCRFVKYAQVMTLYLSTYILMFMAVDRYRAVC--CRNLHWNSLKVAKCF  138
              R  G   G+V+C+       +  + S+  L+ M+ DRY AVC    +    +  ++K
Sbjct  3619  LR--GWIFGNVMCKVYMITTSINQFTSSIFLLIMSADRYIAVCHPISSPKMRTPFISKII  3792

Query  139   VAASWVMAILFSIPQAVIFHEEEISVGVTDCWVQFVEPWGAKAYVTWFVVSI-FGAPLLV  197
               ++W+ +  LF IP   +  +  E + GV  C + +    G     T++++   F  PL+
Sbjct  3793  SISAWITSALFMIPIFLYANTMEYTNGVVSCNIYWPNDHGGHTTFTFYMLIFSFIVPLIF  3972

Query  198   IGVCYGVICRQIWIYsqsalpssqqpptssaypaLTSETGSTLSNRITKAKMKTIKLTLA  257
             I V Y ++ ++                       L +      S    ++  K  +L L
Sbjct  3973  ILVFYLLVIKK-----------------------LRTVGPKNKSKEKKRSHRKVSRLVLT  4083
```

```
Query  258   VVLCFVACWAPFCITQLIMVYCPPTSHVSPVAVIILLLASL----NSCSNPWIYLAFSGS  313
             V++ +V CW P+ +TQ+ ++Y PP    + +++   LLA      NS  NP +Y   S +
Sbjct  4084  VIVVYVFCWLPYWVTQVALIYTPPKQCQTNISIASFLLAGFLSYSNSAMNPILYAFLSDN  4263

Query  314   L  314

Sbjct  4264  F  4266
```

gb|ADTU01001795.1| Atta cephalotes contig01795, whole genome shotgun sequence
Length=5185

```
 Score = 62.8 bits (151),  Expect = 8e-07, Method: Compositional matrix adjust.
 Identities = 78/352 (22%), Positives = 153/352 (43%), Gaps = 51/352 (14%)
 Frame = +3

Query  9     SSSLRDEKLASVEI-STLSV----------ILVLAVTSNLTMLIAIWRQRRNRPLSRMYF  57
             +SS R+    S +I S LSV          I VL +  N+ ++  + R R+   ++ +
Sbjct  1467  NSSARNYSYVSEDITSNLSVQIIFCLFYIIIFVLGIFGNVLVVFVVGRNRQMHTVTNL--  1640

Query  58    FMMHLSLADLLVALFNILPQLAWDITYRFQGGDVLCRFVKYAQVMTLYLSTYILMFMAVD  117
             F+ +L+L+D+L+ +  +     +     +  G  LC  V Y+Q +++Y+ST  L  +AVD
Sbjct  1641  FITNLALSDVLLCVLAVPFTPLYTFLSGWIFGKTLCHLVPYSQGVSVYISTLTLTSIAVD  1820

Query  118   RYRAVCCRNLHWNSLKVAKCFVAASWVMAILFSIPQAVIFHEEEISVGVTDCWVQFVEPW  177
             R+  +         +K+    + + WV+A+L ++P  +    EE  +   +      E W
Sbjct  1821  RFLVIIYPFHPRMKIKMCLAIIVSIWVIALLLTLPYGLYMQLEETYISFCE------ENW  1982

Query  178   GAKAYVTWF--VVSI--FGAPLLVIGVCYGVICRQIWIYsqsalpssqqpptssaypaLT  233
              ++ +   F  + SI  F  P  VI  CY  IC  I +
Sbjct  1983  PSEPFRKVFSSLTSILQFVVPFFVISFCY--ICVSIKL-----------------NDRAR  2105

Query  234   SETGSTLSNRIT---KAKMKTIKLTLAVVLCFVACWAPFCITQLIMVYCPPTSHVSPVAV  290
             ++ G+  S R     + K +T ++ +A+V  F   W P  I  ++  +    +  S   +
Sbjct  2106  AKPGTKTSKREEADRERKRRTNRMLIAMVAIFGVSWLPLNIVNVVDDFYSYANDWSYYKL  2285

Query  291   IILL---LASLNSCSNPWIYLAFSGSLLNQMR---VCSILLLRSTHLFIEQL  336
                +   +A  ++C NP++Y   + +   + +   +C+ LL  S  + + +L
Sbjct  2286  CFFMSHCIAMSSTCYNPFLYAWLNDNFRKEFKQVCICNELLFISLSITLRRL  2441
```

Hits in C.floridanus WGS database:

gb|AEAB01029475.1| Camponotus floridanus CamFlo_1.0_4.contig5512, whole genome shotgun sequence
Length=4331

```
 Score =  108 bits (269),  Expect = 2e-21, Method: Compositional matrix adjust.
 Identities = 48/73 (66%), Positives = 59/73 (81%), Gaps = 0/73 (0%)
 Frame = -2

Query  103   TYRFYGGFLLCKVVKYGQTLGPYLSSYVLMATAIDRHQAICYPLTYCSWTSRRSKVMVYL  162
             T RF GG +LCK++K+GQ  GPYLSSYVL  TAIDR+QAIC+P +YCS TSRRSK+MVY
Sbjct  1714  TIRFQGGAVLCKLIKFGQPFGPYLSSYVLTVTAIDRYQAICHPFSYCSNTSRRSKIMVYG  1535

Query  163   AWVASLAFCIPQL  175
             AWV +   C+PQ+
Sbjct  1534  AWVFAAILCVPQV  1496
```

gb|AEAB01029475.1| Camponotus floridanus
DNTRDEYLARWEIAILTSIFLITLIGNTLILFALYVRRRYQRQKFTRMYFFILHLSVADLLTGLLDVLPQLAWDITFRFQGGAVLCKLIKFGQP
FGPYLSSYVLTVTAIDRYQAICHPFSYCSNTSRRSKIMVYGAWVFAAILCVPQV

```
 Score =  104 bits (259),  Expect = 3e-20, Method: Compositional matrix adjust.
 Identities = 53/80 (66%), Positives = 65/80 (81%), Gaps = 2/80 (3%)
 Frame = -3

Query  26    NNTDRDENLARVEVATLAIIFLVTVIGNSTVLLALWTRRRYAGRKKLSRMYFFILHLSIA  85
             +NT RDE LAR E+A L  IFL+T+IGN+ +L AL+ RRRY  R+K +RMYFFILHLS+A
Sbjct  3132  DNT-RDEYLARWEIAILTSIFLITLIGNTLILFALYVRRRYQ-RQKFTRMYFFILHLSVA  2959

Query  86    DLITAFLSVLPQLAWDITYR  105
             DL+T  L VLPQLAWDIT+R
Sbjct  2958  DLLTGLLDVLPQLAWDITFR  2899
```

```
gb|AEAB01029474.1|  Camponotus floridanus CamFlo_1.0_4.contig5511, whole genome shotgun
sequence
Length=1857

Score = 84.0 bits (206),  Expect = 1e-13, Method: Compositional matrix adjust.
 Identities = 46/103 (45%), Positives = 68/103 (66%), Gaps = 7/103 (7%)
 Frame = -1

Query  207  YSISVFMVPLVVLIFTYTSICIEIWqssess----lrprssqksAPGKRTPLISRAKINT  262
            YSI  F++P +VL++TY SICI IW+S++ S     + ++    +   R P +S+A +NT
Sbjct  816  YSIVQFLLPFIVLVYTYASICIAIWRSNKMSGVIDSKKKNKINFSQQNRNPFMSKAMLNT  637

Query  263  VKQTIAVIVMYIACSTPFILAQLWATWDPQS---PFIDGPVFV  302
            V+QTI VI +YI  S+PFI  +LWATWDP++   PF  G  +V
Sbjct  636  VRQTIIVITLYIVTSSPFIGCELWATWDPKAFSLPFFTGEYYV  508


 Score = 46.6 bits (109),  Expect = 0.10, Method: Compositional matrix adjust.
 Identities = 20/29 (69%), Positives = 23/29 (79%), Gaps = 0/29 (0%)
 Frame = -1

Query  298  GPVFVILTLLYSLNSCVNPWIYLAFNREL  326
            G  F IL+LL SL SCVNPWIY AFN++L
Sbjct  240  GAAFTILSLLNSLTSCVNPWIYFAFNKDL  154


gb|AEAB01012073.1|  Camponotus floridanus CamFlo_1.0_2.contig4201, whole genome shotgun
sequence
Length=8361

 Score = 68.9 bits (167),  Expect = 1e-08, Method: Compositional matrix adjust.
 Identities = 47/170 (28%), Positives = 83/170 (49%), Gaps = 4/170 (2%)
 Frame = -2

Query  11    ISENSTYL-FDKHEDRNNTDRDENLARVEVATLAIIFLVTVIGNSTVLLALWTRRRYAGR  69
             + +++ YL F+      +  R      ++V  LAI+ ++++I N+  + ++   RR   +
Sbjct  4424  VGQSAEYLSFENVTCLEHAPRLTRAVYLQVIVLAIMSVLSLICNAATIYSITKNRR--KQ  4251

Query  70    KKLSRMYFFILHLSIADLITAFLSVLPQLAWDITYRFYGGFLLCKVVKYGQTLGPYLSSY  129
             +  S +Y  ILHLS+ADL+     +     W     +  G   CK+ K+ Q    YLS++
Sbjct  4250  RGCSAIYTLILHLSVADLLVTIFCLAGDAIWSYNVAWLWGNAACKLFKFLQMFSLYLSTF  4071

Query  130   VLMATAIDRHQAICYPLTYCSWTSRRSKVMVYLAWVASLAFCIPQLTIFT  179
             VL+   +DR  A+ YP+   + TS++    V  AW   S     PQ+   T
Sbjct  4070  VLVLIGVDRFVAVRYPMKGLN-TSQKCSRFVLFAWALSFVLATPQVRCIT  3924


gb|AEAB01011169.1| Camponotus floridanus CamFlo_1.0_2.contig3297, whole genome shotgun
sequence
Length=22221

 Score = 68.6 bits (166),  Expect = 2e-08, Method: Compositional matrix adjust.
 Identities = 80/342 (23%), Positives = 147/342 (43%), Gaps = 31/342 (9%)
 Frame = -2

Query  7     SQMDISENSTY-LFDKHEDRNNTDRDE--NLARVEVATLAIIFLVTVIGNSTVLLALWTR  63
             + MD  +NSTY L   H D       E   +A V     +++ +V ++GN+   L ++
Sbjct  7163  TTMDYGQNSTYNLNSTHNDIGQNCEAELPIVALVNQILYSVVCIVGLLGNT---LVIYVV  6993

Query  64    RRYAGRKKLSRMYFFILHLSIADLITAFLSVLPQLAWDITYRFY-GGFLLCKVVKYGQTL  122
              R++  + ++ MY  I++L+IAD    FL  +P L    + R +  G ++CK      ++
Sbjct  6992  LRFSKMQTVTNMY--IVNLAIAD--ECFLIGIPFLVTTFSLRSWIFGKIMCKAYMTTTSI  6825

Query  123   GPYLSSYVLMATAIDRHQAICYPLTYCSW-TSRRSKVMVYLAWVASLAFCIPQLTIFTYT  181
               + SS  L   + DR+ A+C+P++     T   S+V+   AW  S  F +P
Sbjct  6824  NQFTSSIFLFIMSADRYIAVCHPISSPKMRTPFISRVVSLTAWATSALFMVPVFLYANAM  6645

Query  182   SVGEDEYDCWATFQEPWGKRAYVTWYSISV-FMVPLV-VLIFTYTSICIEIWqssesslr  239
                E    C   +    G +   T Y++ + F VPL+ +LIF +  I
Sbjct  6644  ESPEGVVSCNIYWPNDRGGQTSFTLYTLILGFAVPLILILIFYFLVI-------------  6504

Query  240   prssqksAPGKRTPLISRAKINTVKQTIAVIVMYIACSTPFILAQLWATWDPQSPFIDGP  299
              +  +   P  ++    R+     K  + VI +Y+ C  P+ + Q+   + P     +
Sbjct  6503  -KKLRTVGPKNKSKEKKRSHRKVTKLVLTVITVYVFCWLPYWVTQVALIYTPPKQCQNSI  6327
```

```
Query  300   V---FVILTLLYSLNSCVNPWIYLAFNRELPRLLLRHYTASS  338
                 F++   L   NS +NP +Y   +    +  L+  T ++
Sbjct  6326  AITSFLLAGFLSYSNSAMNPILYAFLSDNFKKSFLKACTCAA  6201
```

Hits in H.saltator WGS database:

```
gb|AEAC01024430.1| Harpegnathos saltator strain R22 G/1 HarSal_1.0_3.contig6505,
whole genome shotgun sequence
Length=86885

Score =  108 bits (270),  Expect = 2e-21, Method: Compositional matrix adjust.
 Identities = 63/155 (41%), Positives = 85/155 (55%), Gaps = 30/155 (19%)
 Frame = -2

Query  174   QLTIFTYTSVGEDEYDCWATFQEPWGKRAYVTW--------------------------Y  207
             Q+ IF+Y  +  + ++CWATF   +G+RAY+TW                          Y
Sbjct  7840  QIFIFSYQEISPNVWECWATFHLKYGERAYITW*YI*CRIEYMRLSFQARRCKCEFNRRY  7661

Query  208   SISVFMVPLVVLIFTYTSICIEIWqssesslrp-rssqksAPGKRTPLISRAKINTVKQT  266
             S++ F++P +VL +TY  ICI IW SS+ S          +   R P IS+A INTVKQT
Sbjct  7660  SVTQFLLPFIVLAYTYMRICISIWASSKISGVVDFKKASFSQRNRDPFISKALINTVKQT  7481

Query  267   IAVIVMYIACSTPFILAQLWATWDPQ---SPFIDG  298
             I V+ +YI  S PFI  QLWATWDP+   SPF+ G
Sbjct  7480  IVVVTLYIVTSIPFIGCQLWATWDPRAAISPFLTG  7376


 Score =  105 bits (261),  Expect = 3e-20, Method: Compositional matrix adjust.
 Identities = 54/84 (64%), Positives = 65/84 (77%), Gaps = 2/84 (2%)
 Frame = -1

Query  23    EDRNNTD-RDENLARVEVATLAIIFLVTVIGNSTVLLALWTRRRYAGRKKLSRMYFFILH  81
             E + + D RDE LA+ E+A L  IFL+T+IGN+ VL AL+ RRRY  R K +RMYFFILH
Sbjct  9152  EQKTSVDARDEYLAKWEIAVLTSIFLITLIGNALVLFALYVRRRYQ-RHKFTRMYFFILH  8976

Query  82    LSIADLITAFLSVLPQLAWDITYR  105
             LSIADL+T  L VLPQLAWDIT+R
Sbjct  8975  LSIADLLTGLLDVLPQLAWDITFR  8904


 Score = 98.6 bits (244),  Expect = 4e-18, Method: Compositional matrix adjust.
 Identities = 41/71 (58%), Positives = 56/71 (79%), Gaps = 0/71 (0%)
 Frame = -1

Query  105   RFYGGFLLCKVVKYGQTLGPYLSSYVLMATAIDRHQAICYPLTYCSWTSRRSKVMVYLAW  164
             RF GG +LCK++K+GQ  G YLSSY+L  TA+DR+ AIC+P +YC+ TSRRSK+MVY AW
Sbjct  8432  RFQGGAVLCKLIKFGQPFGLYLSSYILTVTAMDRYYAICHPFSYCNVTSRRSKMMVYAAW  8253

Query  165   VASLAFCIPQL  175
               ++  C+PQ+
Sbjct  8252  TLAVVLCVPQV  8220


 Score = 51.6 bits (122),  Expect = 0.004, Method: Compositional matrix adjust.
 Identities = 26/44 (59%), Positives = 30/44 (68%), Gaps = 1/44 (2%)
 Frame = -2

Query  298   GPVFVILTLLYSLNSCVNPWIYLAFNRELPRLLLRHYTASSKNY  341
             G  F IL LL SL SCVNPWIY AFNREL R+ L ++    K+Y
Sbjct  7114  GAAFTILCLLNSLTSCVNPWIYFAFNREL-RVALTNFFCKRKDY  6986


gb|AEAC01010319.1|  Harpegnathos saltator strain R22 G/1 HarSal_1.0_2.contig1075,
whole genome shotgun sequence
Length=91892

 Score = 68.6 bits (166),  Expect = 2e-08, Method: Compositional matrix adjust.
 Identities = 47/174 (27%), Positives = 88/174 (51%), Gaps = 11/174 (6%)
 Frame = +2

Query  5      KLSQMDISENSTYLFDKHEDRNNTDRDENLARVEVATLAIIFLVTVIGNSTVLLALWTRR  64
               LS++D  EN T L        +  +  +   ++V  LA+I +++++ N   + ++   R
Sbjct  67235  NLSEIDPHENITCL-------EHAPKLTDSVYLKVIILAVISVLSLLCNLATIYSITKNR  67393
```

```
Query  65     RYAGRKKLSRMYFFILHLSIADLITAFLSVLPQLAWDITYRFYGGFLLCKVVKYGQTLGP  124
              R   +++ S +Y  +LHL+IADL+     +  +  W     +  G   CK  K+ Q
Sbjct  67394  R---KQRGSTIYTLLLHLTIADLLVTVFCLAGEAIWSYNVAWLWGNAACKFFKFLQMFSL  67564

Query  125    YLSSYVLMATAIDRHQAICYPLTYCSWTSRRSKVMVYLAWVASLAFCIPQLTIF  178
              YLS++VL+   +DR  A+ YP+   S    + S+ +++ AW+ S    +PQ+  F
Sbjct  67565  YLSTFVLVLIGVDRFVAVRYPMKGLSMNQKCSRFVLF-AWILSCILALPQVRHF  67723
```

gb|AEAC01016841.1| Harpegnathos saltator strain R22 G/1 HarSal_1.0_2.contig7597, whole genome shotgun sequence
Length=85269

```
 Score = 65.5 bits (158),  Expect = 2e-07, Method: Compositional matrix adjust.
 Identities = 82/348 (24%), Positives = 152/348 (44%), Gaps = 35/348 (10%)
 Frame = -2

Query  1      MYTPKLSQMDISENSTYLFDKHEDRNNTDRDENL---ARVEVATLAIIFLVTVIGNSTVL  57
              M    +  +D+ EN TY    +    + + + NL    A V     +I+ +V ++GN+
Sbjct  40211  MMNATMDMIDLFENLTY----NHSTISPNCEANLPIIALVNQVLYSIVCVVGLLGNT---  40053

Query  58     LALWTRRRYAGRKKLSRMYFFILHLSIADLITAFLSVLPQLAWDITYRFY-GGFLLCKVV  116
              L ++   R++  + ++ MY  I++L+IAD    FL  +P L   I+ R +  G ++CKV
Sbjct  40052  LVIYVVLRFSKMQTVTNMY--IVNLAIAD--ECFLIGIPFLVTTISLRSWIFGKIMCKVY  39885

Query  117    KYGQTLGPYLSSYVLMATAIDRHQAICYPLTYCSW-TSRRSKVMVYLAWVASLAFCIPQL  175
                  ++  + SS  L   + DR+ A+C+P+       T   S+V+   AW  S  F +P
Sbjct  39884  MITTSINQFTSSIFLFIMSADRYVAVCHPIFSPKIRTPFISRVVSLSAWATSALFMVPVF  39705

Query  176    TIFTYTSVGEDEYDCWATFQEPWGKRAYVTWYS-ISVFMVPLVVLIFTYTSICIEIWqss  234
                       E    C    + +  G +    T Y+ I  F VPL++++  Y  +  ++
Sbjct  39704  LYANAMESLEGVVSCNIYWPDDHGGQTTFTLYTFILGFAVPLLLILNFYFLVIRKL----  39537

Query  235    esslrprssqksAPGKRTPLISRAKINTVKQTIAVIVMYIACSTPFILAQLWATWDP---  291
                       +   P  ++     R+      K  + VI +Y+ C  P+ + Q+   + P
Sbjct  39536  ---------RTVGPKNKSKEKKRSHRKVTKLVLTVITVYVFCWLPYWVTQVALIYTPPKQ  39384

Query  292    -QSPFIDGPVFVILTLLYSLNSCVNPWIYLAFNRELPRLLLRHYTASS  338
               QS  I    F++   L    NS +NP +Y   +    +  L+  T ++
Sbjct  39383  CQSS-ISITSFLLAGFLSYSNSAMNPILYAFLSDNFKKSFLKACTCAA  39243
```

tBLASTn results of (putative) ant allatostatin receptors

Query sequence 1:

```
>tr|Q9U721|Q9U721_DROME Allatostatin receptor OS=Drosophila melanogaster
GN=AlstR PE=2 SV=1
MAGHQSLALLLATLISSWPKASWGATGNGSIISVSNSSGNNYAFTSEHTDHSDHNANDSM
EYDAESVALERIVSTIVPVFFGIIGFAGLLGNGLVILVVVANQQMRSTTNLLIINLAVSD
ILFVIFCVPFTATDYVLPEWPFGNVWCKFVQYMIVVTCHCSVYTLVLMSFDRFLAVVHPV
TSMSLRTERNATLAIMCAWITIVTTAIPVALSHSVRIYQYHGNAGTACVFSTEEEIWSLV
GFQVSFFLSSYVAPLTLICFLYMGMLARLWKSAPGCKPSAESRKGKRRVTRMVVVVVLAF
AICWLPIHVILVLKALNLYGGSHLSVIIQIISHVVAYTNSCINPILYAFLSDNFRKAFRK
VVWCGSPPPLMTNQQVTKTTRTATGNGTSNIEML
```

Hits in A.cephalotes WGS database:

```
gb|ADTU01019622.1| Atta cephalotes contig19622, whole genome shotgun sequence
Length=20912

Score = 89.7 bits (221),  Expect(2) = 4e-34, Method: Compositional matrix adjust.
 Identities = 73/162 (45%), Positives = 94/162 (58%), Gaps = 30/162 (19%)
 Frame = +1

Query  172   RFLAVVHPVTSMSLRTERNATLAIMCAWITIVTTAIPVALSHS-----------------  214
             R+LAVVHP+ SMS RTE +A  AI   W+ I+T +IP  + H
Sbjct  6988  RYLAVVHPIASMSWRTEIHAIHAICILWVMILTLSIPAFVIHGEVSELHPNRNVSNSDVV  7167

Query  215   ----VRI---YQYHGNAGTACVFSTEEEIWSLVGFQVSFFLSSYVAPLTLICFLYMGMLA  267
                 V +   Y + G   TAC    + + W+   +QV FFLSSYV PL LIC  YM MLA
Sbjct  7168  L*CIVYL*IHYIFQGQNLTACRILDQYD-WT--SYQVPFFLSSYVMPLVLICVFYMFMLA  7338

Query  268   RLWKSAPGCKPSAESRKGKrrvtrmvvvvvLAFAICWLPIHV  309
             RLW+   G + SAE+R+G+RRVTR+VV+VV+ FA CW PI V
Sbjct  7339  RLWR---GVRTSAENRRGRRRVTRLVVIVVVVFAFCWCPIQV  7455


 Score = 86.3 bits (212),  Expect(2) = 4e-34, Method: Compositional matrix adjust.
 Identities = 35/58 (60%), Positives = 52/58 (90%), Gaps = 0/58 (0%)
 Frame = +2

Query  307   IHVILVLKALNLYGGSHLSVIIQIISHVVAYTNSCINPILYAFLSDNFRKAFRKVVWC  364
             + +ILVLK+L++Y  +  ++++QI+SH++AYTNSCINP LYAFLSD+FRKAFRK++C
Sbjct  7535  LQMILVLKSLDMYPLTTATIMVQIVSHILAYTNSCINPFLYAFLSDHFRKAFRKIIYC  7708


 Score =  121 bits (304),  Expect = 8e-26, Method: Compositional matrix adjust.
 Identities = 81/136 (60%), Positives = 101/136 (74%), Gaps = 5/136 (4%)
 Frame = +1

Query  44    FTSEHTD-HSDHNANDSMEYDAESVALERIVSTIVPVffgiigfagllgnglvilvvvAN  102
             FTS   D  +  N     EY+     + RI+  +VP+FFGIIG  GL+GN LV++VV AN
Sbjct  4000  FTSFEGDVETCRNYYHGFEYEF----VHRILVVVVPLFFGIIGILGLVGNLLVVVVVAAN  4167

Query  103   QQMRSTTNLLIINLAVSDILFVIFCVPFTATDYVLPEWPFGNVWCKFVQYMIVVTCHCSV  162
               MRSTTN+LIINLAV+D+LFV+FC+PFTATD+VLP WPFGN+WCK VQYMI+VT + SV
Sbjct  4168  PGMRSTTNILIINLAVADLLFVMFCIPFTATDFVLPYWPFGNIWCKIVQYMIIVTAYASV  4347

Query  163   YTLVLMSFDRFLAVVH  178
             YTL+LMS DR +   H
Sbjct  4348  YTLILMSLDRLVCQDH  4395
```

Query sequence 2:

```
>sp|Q8WPA2|AR_BOMMO Allatostatin-A receptor OS=Bombyx mori GN=AR PE=2 SV=1
MESTEDEFYTICLNLTAEDPSFGNCNYTTDFENGELLEKVVSRVVPIFFGFIGIVGLVGN
ALVVLVVAANPGMRSTTNLLIINLAVADLLFVIFCVPFTATDYVMPRWPFGDWWCKVVQY
FIVVTAHASVYTLVLMSLDRFMAVVHPIASMSIRTEKNALLAIACIWVVILTTAIPVGIC
HGEREYSYFNRNHSSCVFLEERGYSKLGFQMSFFLSSYVIPLALISVLYMCMLTRLWKSA
PGGRVSAESRRGRKKVTRMVVVVVVVFAVCWCPIQIILLVKALNKYHITYFTVTAQIVSH
VLAYMNSCVNPVLYAFLSENFRVAFRKVMYCPPPYNDGFSGRPQATKTTRTGNGNSCHDI
V
```

Hits in C.floridanus WGS database:

```
gb|AEAB01007356.1| Camponotus floridanus CamFlo_1.0_1.contig7356, whole genome shotgun
sequence
Length=44483

Score =  167 bits (423),  Expect = 9e-42, Method: Compositional matrix adjust.
 Identities = 122/276 (44%), Positives = 153/276 (55%), Gaps = 60/276 (22%)
 Frame = +3

Query  137   SLDRFMAVVHPIASMSIRTEKNALLAIACIWVVILTTAIPVGICHGE-----REYSYFNR  191
             ++ R++AVVHPIASMS RTE +A+LAI   WVVIL   + P   + HGE       YSY
Sbjct  7224  TVRRYLAVVHPIASMSWRTENHAILAIGVTWVVILALSAPALVIHGEVSTRDATYSYPRA  7403

Query  192   NHSSC----------VFLEERG-------------YSKLGFQMSFFLSSYVIPLALISVL  228
             +  S           VF E  G               Y     FQ+SFF  SYV+PL LI +
Sbjct  7404  DSRSLT*FPCT*IRYVFQEGDGPPENLTACRVLPQYDWTSFQVSFFFMSYVLPLVLICIF  7583

Query  229   YMCMLTRLWKSAPGGRVSAESRRGRKKVTRMvvvvvvvFAVCWCPI--------------  274
             YM +L +LW+    G RVSAESRRGR++VTR+V VVV VFA CWCPI
Sbjct  7584  YMSVLIKLWR---GARVSAESRRGRRRVTRLVFVVVGVFAACWCPIQVKLSFIGEHPRRF  7754

Query  275   --------------QIILLVKALNKYHITYFTVTAQIVSHVLAYMNSCVNPVLYAFLSEN  320
                           Q+IL+ K+L+ + +T   T+  QIVSH+LAY NSCVNP LYAFLS+N
Sbjct  7755  SHFIPDVIPIVIVSQVILVSKSLDVFPLTTTTIMVQIVSHILAYTNSCVNPFLYAFLSDN  7934

Query  321   FRVAFRKVMYCPPPYNDGFSGRPQATKTTRTGNGNS  356
             FR AFRK++YC P          P AT+TTR  +
Sbjct  7935  FRKAFRKIIYCRPRAEQNNRLGP-ATRTTRAASSGD  8039


 Score =  130 bits (328),  Expect = 3e-29, Method: Compositional matrix adjust.
 Identities = 90/137 (66%), Positives = 111/137 (81%), Gaps = 8/137 (6%)
 Frame = +3

Query  13    LNLTAEDPSFGNCN-------YTTDFENGELLEKVVSRvvpiffgfigivglvgnalvvl  65
             +NLT    +  +C+       Y  D+ N EL+E +V  VVP+FFG IGI+GL GN+LVV+
Sbjct  4191  VNLTNASDALADCSLRFACEVYEFDY-NRELVESIVVVVVPLFFGIIGILGLAGNSLVVV  4367

Query  66    vvaanPGMRSTTNLLIINLAVADLLFVIFCVPFTATDYVMPRWPFGDWWCKVVQYFIVVT  125
             VVAANPGMRSTTN+LIINLA+ADLLFVIFC+PFTATD+V+P WPFG+ WCK+VQY I+VT
Sbjct  4368  VVAANPGMRSTTNILIINLAIADLLFVIFCIPFTATDFVLPYWPFGNVWCKIVQYLIIVT  4547

Query  126   AHASVYTLVLMSLDRFM  142
             A+ASVYTLVLMSLDR +
Sbjct  4548  AYASVYTLVLMSLDRLV  4598
```

Query sequence 3:

```
>gi|48139558|ref|XP_397024.1| PREDICTED: allatostatin-A receptor-like [Apis
mellifera]
MLQEMEITSERVIKNLTNVSNSMNNNETLRYNEEFDYTFDRDQVEKIVVVVVPIFFGMIGILGLVGNSLV
VIVVAANPTMRSTTNILIINLAVADLLFVILCIPFTATDFVLPFWPFGNFWCKMVQYLIIVTAYASVYTL
VLMSLDRYLAVVHPISSMSWRTENHAIIAICIAWAMIFAISTPAFFVHGEDMDGSSSENLTACRILPQYN
WPLFQMSFFLMSYLLPLMLICFFYICMLIRLWRTDRVSAESRRGRKRVTRLVFVVVGVFAFCWCPIQVIL
VTKSLDVYPLTSATIMVQIASHILAYTNSCVNPILYAFLSDSFRKAFRKIIYCRPRSEQNRQLGPLTKTT
RAASTGDII
```

Hits in A.cephalotes WGS database:

```
gb|ADTU01019622.1| Atta cephalotes contig19622, whole genome shotgun sequence
Length=20912


Score =  139 bits (351),  Expect(2) = 3e-58, Method: Compositional matrix adjust.
 Identities = 64/84 (76%), Positives = 74/84 (88%), Gaps = 0/84 (0%)
 Frame = +2

Query  276   IQVILVTKSLDVYPLTSATIMVQIASHILAYTNSCVNPILYAFLSDSFRKAFRKIIYCRP  335
             +Q+ILV KSLD+YPLT+ATIMVQI SHILAYTNSC+NP LYAFLSD FRKAFRKIIYCRP
Sbjct  7535  LQMILVLKSLDMYPLTTATIMVQIVSHILAYTNSCINPFLYAFLSDHFRKAFRKIIYCRP  7714

Query  336   RSEQNRQLGPLTKTTRAASTGDII  359
              +  + Q+GP T+TTRAAS GDI+
Sbjct  7715  TTNSDNQMGPATRTTRAASAGDIL  7786


 Score =  116 bits (291),  Expect(2) = 3e-58, Method: Compositional matrix adjust.
 Identities = 92/156 (59%), Positives = 109/156 (70%), Gaps = 24/156 (15%)
 Frame = +1

Query  147   RYLAVVHPISSMSWRTENHaiiaiciawamifaiSTPAFFVHGE------DMDGSSS---  197
             RYLAVVHPI+SMSWRTE HAI AICI W MI  +S PAF +HGE      + + S+S
Sbjct  6988  RYLAVVHPIASMSWRTEIHAIHAICILWVMILTLSIPAFVIHGEVSELHPNRNVSNSDVV  7167

Query  198   ---------------ENLTACRILPQYNWPLFQMSFFLMSYLLPLMLICFFYICMLIRLW  242
                            +NLTACRIL QY+W  +Q+ FFL SY++PL+LIC FY+ ML RLW
Sbjct  7168  L*CIVYL*IHYIFQGQNLTACRILDQYDWTSYQVPFFLSSYVMPLVLICVFYMFMLARLW  7347

Query  243   RTDRVSAESRRGrkrvtrlvfvvvgvfafCWCPIQV  278
             R  R SAE+RRGR+RVTRLV +VV VFAFCWCPIQV
Sbjct  7348  RGVRTSAENRRGRRRVTRLVVIVVVVFAFCWCPIQV  7455


 Score =  148 bits (373),  Expect = 3e-35, Method: Compositional matrix adjust.
 Identities = 94/124 (76%), Positives = 106/124 (85%), Gaps = 4/124 (3%)
 Frame = +1

Query  30    RYNEEFDYTFDRDQVEKIvvvvvPIFFGMIGILGLVGNSLVVIVVAANPTMRSTTNILII  89
              Y   F+Y F    V +I+VVVVP+FFG+IGILGLVGN LVV+VVAANP MRSTTNILII
Sbjct  4036  NYYHGFEYEF----VHRILVVVVPLFFGIIGILGLVGNLLVVVVVAANPGMRSTTNILII  4203

Query  90    NLAVADLLFVILCIPFTATDFVLPFWPFGNFWCKMVQYLIIVTAYASVYTLVLMSLDRYL  149
             NLAVADLLFV+ CIPFTATDFVLP+WPFGN WCK+VQY+IIVTAYASVYTL+LMSLDR +
Sbjct  4204  NLAVADLLFVMFCIPFTATDFVLPYWPFGNIWCKIVQYMIIVTAYASVYTLILMSLDRLV  4383

Query  150   AVVH  153
                H
Sbjct  4384  CQDH  4395
```

Hits in C.floridanus WGS database:

```
gb|AEAB01007356.1| Camponotus floridanus CamFlo_1.0_1.contig7356, whole genome shotgun
sequence
Length=44483

Score =  256 bits (654),  Expect = 1e-72, Method: Compositional matrix adjust.
 Identities = 164/275 (60%), Positives = 191/275 (69%), Gaps = 60/275 (22%)
 Frame = +3

Query  144   SLDRYLAVVHPISSMSWRTENHaiiaiciawamifaiSTPAFFVHGE-------------  190
             ++ RYLAVVHPI+SMSWRTENHAI+AI + W +I A+S PA  +HGE
Sbjct  7224  TVRRYLAVVHPIASMSWRTENHAILAIGVTWVVILALSAPALVIHGEVSTRDATYSYPRA  7403

Query  191   ------------------DMDGSSSENLTACRILPQYNWPLFQMSFFLMSYLLPLMLICF  232
                               + DG   ENLTACR+LPQY+W  FQ+SFF MSY+LPL+LIC
Sbjct  7404  DSRSLT*FPCT*IRYVFQEGDGPP-ENLTACRVLPQYDWTSFQVSFFFMSYVLPLVLICI  7580

Query  233   FYICMLIRLWRTDRVSAESRRGrkrvtrlvfvvvgvfafCWCPI----------------  276
             FY+ +LI+LWR  RVSAESRRGR+RVTRLVFVVVGVFA CWCPI
Sbjct  7581  FYMSVLIKLWRGARVSAESRRGRRRVTRLVFVVVGVFAACWCPIQVKLSFIGEHPRRFSH  7760
```

```
Query  277   ------------QVILVTKSLDVYPLTSATIMVQIASHILAYTNSCVNPILYAFLSDSFR  324
                         QVILV+KSLDV+PLT+ TIMVQI SHILAYTNSCVNP LYAFLSD+FR
Sbjct  7761  FIPDVIPIVIVSQVILVSKSLDVFPLTTTTIMVQIVSHILAYTNSCVNPFLYAFLSDNFR  7940

Query  325   KAFRKIIYCRPRSEQNRQLGPLTKTTRAASTGDII  359
             KAFRKIIYCRPR+EQN +LGP T+TTRAAS+GDI+
Sbjct  7941  KAFRKIIYCRPRAEQNNRLGPATRTTRAASSGDIL  8045


 Score =  186 bits (473),  Expect = 2e-48, Method: Compositional matrix adjust.
 Identities = 95/115 (83%), Positives = 107/115 (93%), Gaps = 0/115 (0%)
 Frame = +3

Query  33    EEFDYTFDRDQVEKIvvvvvPIFFGMIGILGLVGNSLVVIVVAANPTMRSTTNILIINLA  92
             E +++ ++R+ VE IVVVVVP+FFG+IGILGL GNSLVV+VVAANP MRSTTNILIINLA
Sbjct  4248  EVYEFDYNRELVESIVVVVVPLFFGIIGILGLAGNSLVVVVVAANPGMRSTTNILIINLA  4427

Query  93    VADLLFVILCIPFTATDFVLPFWPFGNFWCKMVQYLIIVTAYASVYTLVLMSLDR  147
             +ADLLFVI CIPFTATDFVLP+WPFGN WCK+VQYLIIVTAYASVYTLVLMSLDR
Sbjct  4428  IADLLFVIFCIPFTATDFVLPYWPFGNVWCKIVQYLIIVTAYASVYTLVLMSLDR  4592
```

Hits in H.saltator WGS database:

```
gb|AEAC01015547.1| Harpegnathos saltator strain R22 G/1 HarSal_1.0_2.contig6303,
whole genome shotgun sequence
Length=85976

Score =  238 bits (607),  Expect(2) = 3e-69, Method: Compositional matrix adjust.
 Identities = 137/207 (66%), Positives = 155/207 (75%), Gaps = 37/207 (18%)
 Frame = -1

Query  190    EDMDGSSSENLTACRILPQYNWPLFQMSFFLMSYLLPLMLICFFYICMLIRLWRTDRVSA  249
              +++DGSSSENLTACRIL QY+W  FQ+SFFL SY+LPL LIC FY+CMLI+LWR  R+SA
Sbjct  84554  QEVDGSSSENLTACRILEQYDWSFFQVSFFLTSYVLPLKLICIFYVCMLIKLWRGARISA  84375

Query  250    ESRRGrkrvtrlvfvvvgvfafCWCPIQV------------------------------  278
              ESRRGR+RVTRLV VVVGVFA CWCPIQV
Sbjct  84374  ESRRGRRRVTRLVLVVVGVFAVCWCPIQVKVSL*AGERERERERESNLCFSISSCRDVVR  84195

Query  279    ------ILVTKSLDVYPLTSATIMVQIASHILAYTNSCVNPILYAFLSDSFRKAFRKIIY  332
                    ILVTKS+D++PLT+ATIM+QIASHILAYTNSCVNP LYAFLSD+FRKAFRKIIY
Sbjct  84194  NVTLQVILVTKSVDMFPLTTATIMLQIASHILAYTNSCVNPFLYAFLSDNFRKAFRKIIY  84015

Query  333    CRPRSEQNRQLGPLTKTTRAASTGDII  359
              CRPR E N +LGP TKTTRAASTGDI+
Sbjct  84014  CRPRPESNNRLGPATKTTRAASTGDIL  83934


 Score = 55.5 bits (132),  Expect(2) = 3e-69, Method: Compositional matrix adjust.
 Identities = 35/44 (80%), Positives = 40/44 (91%), Gaps = 0/44 (0%)
 Frame = -2

Query  147    RYLAVVHPISSMSWRTENHaiiaiciawamifaiSTPAFFVHGE  190
              RYLAVVHPI+SMSWRTENHAI+AIC+ WA+I AISTPA  +HGE
Sbjct  84775  RYLAVVHPIASMSWRTENHAILAICVTWAVILAISTPALVIHGE  84644
```

tBLASTn results of (putative) ant tachykinin receptors

Query sequence 1:

```
>tr|E2AB29|E2AB29_CAMFO Tachykinin-like peptides receptor 99D OS=Camponotus
floridanus GN=EAG_01045 PE=4 SV=1
MAIVNPLRPRMGKKATLCVAIVIWIVGAILSLPMLVFYTTYTQNFMNGEVRVICYSDWPN
RDDNGLSYDEYLYNVIFMILTYFLPIGSMTFTYARVGLELWGSQSIGEATARQLENIRSK
RRVVKMMIVVVVIFAVCWLPFHVYFIVTSYLPEITNEPYIQEVFLGIYWLAMSNSMYNPI
IYCWMNTRFRRGFAQFFFWCPWVRITTEPSLSRSEAVTSRYSCTGSPDMHTRISRNGTVR
LPLHLQSMRGGNDRLLAHHRGHGRKWRTSQTRGHVS
```

Hits in A.cephalotes WGS database:

```
gb|ADTU01004570.1| Atta cephalotes contig04570, whole genome shotgun sequence
Length=21306


Score =  125 bits (315),  Expect = 2e-28, Method: Compositional matrix adjust.
 Identities = 66/72 (92%), Positives = 71/72 (99%), Gaps = 0/72 (0%)
 Frame = +3

Query  1    MAIVNPLRPRMGKKATLCVAIVIWIVGAILSLPMLVFYTTYTQNFMNGEVRVICYSDWPN  60
            MAIVNPL+PRMGKKATLCVAI+IWI+GAILSLPML+FYTTYTQNFMNGEVRV+CYSDWPN
Sbjct  726  MAIVNPLKPRMGKKATLCVAIIIWIIGAILSLPMLLFYTTYTQNFMNGEVRVVCYSDWPN  905

Query  61   RDDNGLSYDEYL  72
             DDNGLSYDEYL
Sbjct  906  TDDNGLSYDEYL  941


 Score =  117 bits (293),  Expect = 1e-25, Method: Compositional matrix adjust.
 Identities = 54/55 (98%), Positives = 55/55 (100%), Gaps = 0/55 (0%)
 Frame = +3

Query  134    FAVCWLPFHVYFIVTSYLPEITNEPYIQEVFLGIYWLAMSNSMYNPIIYCWMNTR  188
              FAVCWLPFH+YFIVTSYLPEITNEPYIQEVFLGIYWLAMSNSMYNPIIYCWMNTR
Sbjct  17436  FAVCWLPFHMYFIVTSYLPEITNEPYIQEVFLGIYWLAMSNSMYNPIIYCWMNTR  17600


 Score =  110 bits (276),  Expect = 2e-23, Method: Compositional matrix adjust.
 Identities = 50/57 (88%), Positives = 52/57 (91%), Gaps = 0/57 (0%)
 Frame = +2

Query  188    RFRRGFAQFFFWCPWVRITTEPSLSRSEAVTSRYSCTGSPDMHTRISRNGTVRLPLH  244
              RFRRGFAQFFFWCPWVR+T+EPSLSRSEAVTSRYSCTGSPDMHTRISRNGT     H
Sbjct  20783  RFRRGFAQFFFWCPWVRVTSEPSLSRSEAVTSRYSCTGSPDMHTRISRNGTCTFSEH  20953


 Score = 97.4 bits (241),  Expect = 1e-18, Method: Compositional matrix adjust.
 Identities = 47/47 (100%), Positives = 47/47 (100%), Gaps = 0/47 (0%)
 Frame = +2

Query  73    YNVIFMILTYFLPIGSMTFTYARVGLELWGSQSIGEATARQLENIRS  119
             YNVIFMILTYFLPIGSMTFTYARVGLELWGSQSIGEATARQLENIRS
Sbjct  4766  YNVIFMILTYFLPIGSMTFTYARVGLELWGSQSIGEATARQLENIRS  4906
```

Query sequence 2:

```
>tr|E2ALS3|E2ALS3_CAMFO Tachykinin-like peptides receptor 86C OS=Camponotus
floridanus GN=EAG_10587 PE=4 SV=1
MTVMAVCYACMGRKLWGSKSIGELTHYQKESMKSKRKVVKMFIIVVTIFAVCWLPYQGFF
IFVYHYRHFTESSYVQHVYLGFYWLAMSNSMVNPIIYYWMNSRFRVYFDLVICKCCCVVD
RTNVRRRETQELTGLQRSELVPCNSGRFKSTSIRWRQSMAESQVQTFKTNRTMCNSLQLK
EDVAII
```

Hits in A.cephalotes WGS database:

```
gb|ADTU01007800.1|  Atta cephalotes contig07800, whole genome shotgun sequence
Length=6963

Score =  133 bits (335),  Expect = 2e-32, Method: Compositional matrix adjust.
 Identities = 61/68 (90%), Positives = 66/68 (97%), Gaps = 0/68 (0%)
 Frame = +2

Query  37    KVVKMFIIVVTIFAVCWLPYQGFFIFVYHYRHFTESSYVQHVYLGFYWLAMSNSMVNPII  96
             +VVKMFIIVVTIFAVCWLPYQGFFIFVYH+RHF E+SYVQHVYL FYWLAMSNSMVNPII
Sbjct  2009  QVVKMFIIVVTIFAVCWLPYQGFFIFVYHHRHFAENSYVQHVYLSFYWLAMSNSMVNPII  2188

Query  97    YYWMNSRF  104
             YYWMN+R+
Sbjct  2189  YYWMNNRY  2212


 Score = 79.3 bits (194),  Expect = 1e-13, Method: Compositional matrix adjust.
 Identities = 37/45 (82%), Positives = 41/45 (91%), Gaps = 0/45 (0%)
 Frame = +3

Query  1     MTVMAVCYACMGRKLWGSKSIGELTHYQKESMKSKRKVVKMFIIV  45
             M+VMAVCY CMGRKLWGSKSIGELTHYQKE+MKSKRKV   FI++
Sbjct  927   MSVMAVCYTCMGRKLWGSKSIGELTHYQKEAMKSKRKVRYTFILL  1061
```

Hits in H.saltator WGS database:

```
gb|AEAC01003153.1| Harpegnathos saltator strain R22 G/1 HarSal_1.0_1.contig3153,
whole genome shotgun sequence
Length=31451

Score =  132 bits (332),  Expect = 4e-32, Method: Compositional matrix adjust.
 Identities = 74/155 (48%), Positives = 98/155 (63%), Gaps = 16/155 (10%)
 Frame = +2

Query  37     KVVKMFIIVVTIFAVCWLPYQGFFIFVYHYRHFTESSYVQHVYLGFYWLAMSNSMVNPII  96
              +VVKMF +VVTIFAVCWLPYQGFFIFVYH+RHF ESSYVQHV+L FYWLAMSNSMVNPII
Sbjct  12563  QVVKMFGVVVTIFAVCWLPYQGFFIFVYHHRHFAESSYVQHVFLSFYWLAMSNSMVNPII  12742

Query  97     YYWMNSRFRVYFDLVICKCCCVVDRTNVRRRET-----QELTGLQRSELVPCNSGRFKST  151
              YYWMN+R   YF ++       V +  + +R       +     +    + C +   +S
Sbjct  12743  YYWMNNR---YFTILTLSDMNSVVKLFLNKRSAVIFF*KNNFTFKVMLFLNCYANVMESN  12913

Query  152    SIRWRQSMAESQVQTFKTNRTMCNSLQLKEDVAII  186
              ++    Q M +   +  FKT++     L ++E+  II
Sbjct  12914  NL---QEMCKINLSRFKTDK-----LNVREEFEII  12994


 Score = 62.8 bits (151),  Expect = 7e-08, Method: Compositional matrix adjust.
 Identities = 32/58 (55%), Positives = 38/58 (66%), Gaps = 2/58 (3%)
 Frame = +1

Query  103    RFRVYFDLVICKCCCVVDRTNVRRRETQELTGLQRSELVPCNSGRFKSTSIRWRQSMA  160
              RFRVYF LVICKCC   + R N +   E QELTG QRS  V CNSG + S   +  R ++A
Sbjct  17284  RFRVYFKLVICKCCRAIGRKNTQSHEMQELTGFQRS--VACNSGEYSSFLLGLRDNVA  17451


 Score = 58.9 bits (141),  Expect = 1e-06, Method: Compositional matrix adjust.
 Identities = 28/38 (74%), Positives = 32/38 (84%), Gaps = 0/38 (0%)
 Frame = +1

Query  1      MTVMAVCYACMGRKLWGSKSIGELTHYQKESMKSKRKV  38
              MTVM VCY  MGR+L  S+SIGE+TH Q+ESMKSKRKV
Sbjct  11476  MTVMIVCYGLMGRELCCSRSIGEVTHNQRESMKSKRKV  11589


 Score = 55.8 bits (133),  Expect = 2e-05, Method: Compositional matrix adjust.
 Identities = 26/42 (62%), Positives = 33/42 (79%), Gaps = 1/42 (2%)
 Frame = +1

Query  146    GRFKSTSIRWRQSMAESQVQTFKTN-RTMCNSLQLKEDVAII  186
              GR++STSI+WR S AESQV TFK N RT+C  +Q KE ++II
Sbjct  20284  GRYRSTSIKWRHSTAESQVHTFKINPRTVCEKIQSKEGISII  20409
```

Query sequence 3:

```
>tr|E2B749|E2B749_HARSA Tachykinin-like peptides receptor 86C
OS=Harpegnathos saltator GN=EAI_02552 PE=4 SV=1
MTVMIVCYGLMGRELCCSRSIGEVTHNQRESMKSKRKVVKMFGVVVTIFAVCWLPYQGFF
IFVYHHRHFAESSYVQHVFLSFYWLAMSNSMVNPIIYYWMNNRYFTILTLSDMNSVVKLF
LNKRSAVIFF
```

Hits in A.cephalotes WGS database:

```
gb|ADTU01007800.1|  Atta cephalotes contig07800, whole genome shotgun sequence
Length=6963

Score =  138 bits (348),  Expect = 3e-35, Method: Compositional matrix adjust.
 Identities = 64/71 (90%), Positives = 70/71 (99%), Gaps = 0/71 (0%)
 Frame = +2

Query  37    KVVKMFGVVVTIFAVCWLPYQGFFIFVYHHRHFAESSYVQHVFLSFYWLAMSNSMVNPII  96
             +VVKMF +VVTIFAVCWLPYQGFFIFVYHHRHFAE+SYVQHV+LSFYWLAMSNSMVNPII
Sbjct  2009  QVVKMFIIVVTIFAVCWLPYQGFFIFVYHHRHFAENSYVQHVYLSFYWLAMSNSMVNPII  2188

Query  97    YYWMNNRYFTI  107
             YYWMNNRY++I
Sbjct  2189  YYWMNNRYYSI  2221


 Score = 53.9 bits (128),  Expect = 1e-05, Method: Compositional matrix adjust.
 Identities = 26/38 (68%), Positives = 32/38 (84%), Gaps = 0/38 (0%)
 Frame = +3

Query  1     MTVMIVCYGLMGRELCCSRSIGEVTHNQRESMKSKRKV  38
             M+VM VCY  MGR+L  S+SIGE+TH Q+E+MKSKRKV
Sbjct  927   MSVMAVCYTCMGRKLWGSKSIGELTHYQKEAMKSKRKV  1040
```

Hits in C.floridanus WGS database:

```
gb|AEAB01019143.1|  Camponotus floridanus CamFlo_1.0_3.contig3594, whole genome shotgun
sequence
Length=7789

Score =  130 bits (326),  Expect = 4e-32, Method: Compositional matrix adjust.
 Identities = 60/68 (88%), Positives = 65/68 (96%), Gaps = 0/68 (0%)
 Frame = -3

Query  37    KVVKMFGVVVTIFAVCWLPYQGFFIFVYHHRHFAESSYVQHVFLSFYWLAMSNSMVNPII  96
             +VVKMF +VVTIFAVCWLPYQGFFIFVYH+RHF ESSYVQHV+L FYWLAMSNSMVNPII
Sbjct  2225  QVVKMFIIVVTIFAVCWLPYQGFFIFVYHYRHFTESSYVQHVYLGFYWLAMSNSMVNPII  2046

Query  97    YYWMNNRY  104
             YYWMN+RY
Sbjct  2045  YYWMNSRY  2022


 Score = 57.4 bits (137),  Expect = 9e-07, Method: Compositional matrix adjust.
 Identities = 28/38 (74%), Positives = 32/38 (84%), Gaps = 0/38 (0%)
 Frame = -2

Query  1     MTVMIVCYGLMGRELCCSRSIGEVTHNQRESMKSKRKV  38
             MTVM VCY  MGR+L  S+SIGE+TH Q+ESMKSKRKV
Sbjct  3585  MTVMAVCYACMGRKLWGSKSIGELTHYQKESMKSKRKV  3472
```
