# Supplementary material for: Discovery of Defense- and Neuropeptides in Social Ants by Genome-Mining
Source: PLoS One. 2012 Mar 20;7(3):e32559. doi: 10.1371/journal.pone.0032559 (PMC3308954; doi:10.1371/journal.pone.0032559)
Supplement: File S2 — Annotated DNA and translated (putative) precursor proteins from ants. (PDF) [file pone.0032559.s009.pdf]

Novel peptides (DNA and precursor protein sequences): putative CDS

Sequence ID	GenBank accession
>Cf_pilosulin-like	AEAB00001185.1
>Cf_defensin-1	AEAB01018225.1
>Cf_defensin-2	AEAB01026853.1
>Ac_defensin	ADTU01021145.1
>Hs_defensin-1	AEAC01007503.1
>Hs_defensin-1a	AEAC01007503.1
>Hs_defensin-2	AEAC01015843.1
>Cf_diuretic-hormone-like	AEAB01029697.1
>Ac_diuretic-hormone-like	ADTU01016305.1
>Hs_diuretic-hormone-like	AEAC01024560.1
>Cf_abaecin-like	AEAB01017814.1
>Ac_abaecin-like	ADTU01009315.1
>Hs_abaecin-like	AEAC01003437.1
>Ac_eclosion-hormone-like	ADTU01024031.1
>Ac_neuroparsin-like	ADTU01005355.1
>Cf_allatostatin	AEAB01013854.1
>Hs_allatostatin	AEAC01017332.1
>Ac_allatostatin	ADTU01011027.1
>Hs_inotocin	AEAC01019310.1
>Cf_inotocin	AEAB01028364.1
>Ac_inotocin	ADTU01000445.1
>Cf_ion-transport-peptide-like	AEAB01021789.1
>Ac_ion-transport-peptide-like	ADTU01018997.1
>Hs_ion-transport-peptide	AEAC01008001.1
>Cf_CHH-like	AEAB01021790.1
>Ac_tachykinin-related-peptide	ADTU01000331.1

Pilosulin–like

>Cf_pilosulin-like [organism= Camponotus floridanus] putative precursor protein (partial)
MRLLCLSLALAIIYIMAIMHSSRVEARATTSPDLSSSSSSESWEIPPKPPKPPTSADLDEDAALARPIVNAVSLI
INFVFEGRRSFL*
>Cf_pilosulin-like [organism= Camponotus floridanus] (partial)
ATGCGATTATTGTGTTTGTCATTAGCGCTCGCTATAATCTATATTATGGCAATCATGCACTCTTCTAGAGTGGAA
GCGAGAGCAACCACTTCTCCTGATTTATCTTCTTCTTCTTCTTCCGAAAGTTGGGAAATACCTCCAAAACCTCCA
AAACCACCTACTTCTGCGGATCTCGACGAAGATGCTGCTTTAGCCAGACCAATTGTTAATGCCGTAAGCTTAATT
ATAAATTTCGTATTCGAGGGGAGGAGGAGTTTCCTATAA

Defensins

>Cf_defensin-1 [organism= Camponotus floridanus] putative precursor protein (complete)
MKLLVIFATFAVLAYVSANTLSAVYDGPTYELTTIDEPQYDEMASNLSPIRHRRVTCDLLSWQSQWLTINHSACA
AKCLVQRRRGGRCRDGICVCRN*
>Cf_defensin-1 [organism= Camponotus floridanus]
ATGAAGCTGCTCGTTATCTTCGCCACTTTTGCTGTACTAGCTTACGTTTCGGCAAACACACTATCGGCTGTTTAT
GACGGACCTACTTACGAATTGAGTAAGTACGTTTTATCGTAAAAGAGAGGACGACGGAAAAATTTCTCGTAGCTC
AAATGAAAAGTCTCTCGATAATTGAACAATTTTATTAAGCTTTAATTAATTTCAAATAATCTCTCTGCTTTTTTT
TATCGGAGATTTTTTGCGGAGATATCAAGAAAGATATTTGAAAGACAGAGAGATATTGTTAAGTATTAAAAATAC
TAAATATTATTTTTGTATTATGAAGAAATTATGATGCAAATATTAGTGTTTGTTGTTGTTATAGCCTTATTTTTC
GAGGAGAGTAATATTGCATTGTACAATATAGGGAAACTTTTCAACTTTGATTAATAAATATCTAGAATGCATTAT
CTTTTGTCAATAATAATGTGTTATACACATAAATACATATATTCTCTGTCTCGCTCTTTCTCCTTCTCTCTCTAA
TCTTTTCTTTCTCCAATGTACCCTTTTAGCATACATTGATAATACATTGTCGATATCCTTTCAATATGACACGAC
CTTTCTCTCTGATGAATCATTTGACTTATCGCATCATTTCCGAATGCTAAATTAGAAATAGTATTTACTTTACTC
TCTGAAATATCATTTAAATATTAAAATTCAATATTATTTAAAATTAAATATTATTATTAAATATTATTTAAATAT
TAAAATCAATCTTCCGTAATTAAAGTCTCAAATAAAAATTAAATTCTCTTTAAAAACTCGAAATTTATTTAATAA
AAATAACTCCCAATATAAGGTAATTCGTAAAAGAAAGAAGTGCTTTCAAATTTCCTTCAATTTCTCACCTTACTT
TAAACGAAAAAGACAGCGCAAAAGGAAGAATTAACTCTATTCTCGTGAGAGAATGTGCATTCCTGCTACAAAATT
CTCTTTTCGTTCGTCCCGTAAATACGATTTCGCATTGTAACATACATGGTGTCGAATATATATCCTGTGTTCGTG
TAAAAATTCAATATACTCTGTGAAACGCGGAATTTCGGTCTTATTATCGCACGAGGGAAAATAATGGGAAAATTG
CTCGAATTGTTTCAGCCACCATCGACGAGCCGCAATACGACGAGATGGCATCGAATCTGTCGCCCATCCGACACC
GGCGCGTAACCTGCGACCTTTTATCGTGGCAATCCCAATGGTTGACCATAAACCACAGTGCGTGCGCGGCTAAAT
GCCTGGTGCAGCGTCGTCGGGGCGGCCGCTGCCGCGACGGCATCTGCGTCTGCAGGAATTAG

```
//
Gene 1
Gene 25203 26536
  Exon 25203 25299 phase 0
  Exon 26343 26533 phase 1
//
FT          CDS    join(25203..25299,26343..26536)
FT                   /note="Match to DEF2_APIME_Defensin-2"
//
>CamFlo_2676.[25203:26536].sp.tr
MKLLVIFATFAVLAYVSANTLSAVYDGPTYELTTIDEPQYDEMASNLSPIRHRRVTCDLL
SWQSQWLTINHSACAAKCLVQRRRGGRCRDGICVCRN
//
>CamFlo_2676.[25203:26536].sp
ATGAAGCTGCTCGTTATCTTCGCCACTTTTGCTGTACTAGCTTACGTTTCGGCAAACACA
CTATCGGCTGTTTATGACGGACCTACTTACGAATTGACCACCATCGACGAGCCGCAATAC
GACGAGATGGCATCGAATCTGTCGCCCATCCGACACCGGCGCGTAACCTGCGACCTTTTA
TCGTGGCAATCCCAATGGTTGACCATAAACCACAGTGCGTGCGCGGCTAAATGCCTGGTG
CAGCGTCGTCGGGGCGGCCGCTGCCGCGACGGCATCTGCGTCTGCAGGAAT
//
```

>Cf_defensin-2 [organism= Camponotus floridanus] putative precursor protein (complete)
MKIYVFTLLVVTAIAVAFPTEELELEENVTVESPDFLILKKDKSLQETPIKEHNRTRRATCDLLSGFGVNHSACA
AHCILRGKTGGRCNSNAVCVCRA*
>Cf_defensin-2 [organism= Camponotus floridanus]
ATGAAGATCTACGTATTCACTCTTTTGGTGGTGACGGCAATCGCCGTTGCATTCCCTACTGAAGAGCTAGAGCTA
GAGGAAAATGTAACGGTAGAGTCTCCGGACTTTTTGATACTGAAAAAAGATAAATCTTTGCAAGAGACACCTATC

```
AAAGAACATAATCGTACCCGTAGAGCTACCTGTGATCTTTTATCTGGCTTCGGTGTTAATCATAGCGCTTGCGCA
GCTCACTGCATTCTAAGGGGAAAAACCGGGGGAAGATGTAACAGTAACGCTGTTTGCGTGTGTCGCGCGTAA
//
Gene 1
Gene 652 1344
  Exon 652 715 phase 0
  Exon 1115 1344 phase 1
//
FT          CDS    join(652..715,1115..1344)
FT                   /note="Match to DEF_FORAQ_Defensin"
//
> CamFlo_1.0_4.contig2890,.[652:1344].sp.tr
MKIYVFTLLVVTAIAVAFPTEELELEENVTVESPDFLILKKDKSLQETPIKEHNRTRRAT
CDLLSGFGVNHSACAAHCILRGKTGGRCNSNAVCVCRA
//
> CamFlo_1.0_4.contig2890,.[652:1344].sp
ATGAAGATCTACGTATTCACTCTTTTGGTGGTGACGGCAATCGCCGTTGCATTCCCTACT
GAAGAGCTAGAGCTAGAGGAAAATGTAACGGTAGAGTCTCCGGACTTTTTGATACTGAAA
AAAGATAAATCTTTGCAAGAGACACCTATCAAAGAACATAATCGTACCCGTAGAGCTACC
TGTGATCTTTTATCTGGCTTCGGTGTTAATCATAGCGCTTGCGCAGCTCACTGCATTCTA
AGGGGAAAAACCGGGGGAAGATGTAACAGTAACGCTGTTTGCGTGTGTCGCGCGTAA
//
```

```
>Ac_defensin [organism= Atta cephalotes] putative precursor protein
(complete)
MKLFAILVIFVVLACTSVSTLPAVYDGPTYELTTIEDPVNDEMPSDLSPIRNRRVTCDLLSWQSKWLSINHSACA
ARCLSQRRKGGRCRDGICVCRN*
>Ac_defensin [organism= Atta cephalotes] putative
ATGAAGCTGTTCGCTATCCTCGTTATCTTCGTCGTATTGGCTTGTACCTCAGTCAGCACACTACCGGCTGTCTAT
GACGGACCTACTTATGAACTGAGTAAGCATACATTTCACTGAGAAAGACTGAGAAAATAATTCATAAATAAATTT
ATGAGTTATCTTAATCTTCCGGTGCTGACGCGAGCAGCATCTGCTCTAGTAATACAGTACTGATGCTGGGTTTGT
AATATTCTTCTACATCGATAATACTGTCAGTACCAACAAATATTGATAATATTATTGATTGTGTGCGGTTAATAT
TGATGGATTGAATATGGATTTCTTGATTTTTGAGCATCAGAACAAATTGAGCACAGGATACTGTTGCTTGAGAAT
CATCGTTAATAATACTGTCAATATTTATGGGTATTAACAGATTTTTGATTGTATAAAATTCGTAAAATTGAGTTA
AAATATTATTATCAATAATGCACAGAAAGAACAATTGTTGATCAAGTATCTAAAAATTTAGTTAGATTACAGATC
TGAAGATAATTTTGTTGACCATCAAAATAATTATATACATGTTCAAACTGGTTGTTAGAATGTCAAAACTTTTTA
CAGCATTACTCGTGCTTGACATTTTTATATAATTATTTTAATGATCCAACAAAATTATTTTTAAACCTGTATCTA
GCTAAATTTTTAGACTTCAGTAAAACCATTTTTTTCGTATATTAATCATGTTAAATTATAAAAATATTGATGTAT
ACTATCAGTATTACTGATTCAATATTACTAATCGATGTTATTAATCGTAAACAGAAGATTCACTTAATTTAGACT
AATCAAAAAGCACTAATTAATTTCAGAAAATCAAAATAACATAACTCATATTTAAAAAAGAATCAATATTAGGCA
TATTTTAATGAGAATAATCCGAATTTGTAGATGTAATATAAAGAAAATTAAAAAATATAATGACTAACGGGCAAT
TGAGAACAATAATCACGCAATTATTCATGCGTAATAATTAATAAATGTATTGTTCAAGACGTTATATACAAAGTA
ATTATGTTTATGGCGGAATCGTCATTTCAACAATTTATTTGGTGCGAACCAGTACTCTGAACAAATTTTGCGTAT
CCATTCTTTATAAATAACTTATTGTACTTTTTCAATATTGATCATAATTAGCAGCTGTGCTCACTCGGCTTATTG
ACATGAATGTTTCTCAAACTTCCTCAACTGCTCTTTTATATTTATTTTATCCGATTCGATTAATTTTTGAATCTA
CCATAGGACATTGCTGATGATGACTCGAATGGAGCCGAAATGTTCGATTCTTCTTATTTATATGTAACGTCTAGA
ACAATACATTTATTAATTATTACGCACGAATAATTGCATTGATTATTGCTTTCAATTGCCTGTTAATCATTACAT
TTTTTATCTCAAAAGCTCAATTTATTTTACTTTTATATTATTATATATTACTTTTATAATAAAGAAAATTGTTTG
TCATTTTTGACAGAGAGATTTTAATTGAGAACCAGCAATATATACATATATCAGTTTCGAAATTTAGATTTAAAT
AATAGTCAAGTTCCTTTAGTAATGGAGCGACTCTTAACAAGTTGTAATTAATCTTAGATTAATCTATATATAATT
CTTACTTTATCAGAGATTTATTTCACTGAGATATCAAGTTTGAGTTACATTAAGTAACTTTTTTGTCGATAATTA
GATCGGATATTATAATAAATGTTTGGCTCGTTGAATATTATACCGTAATTTTATTTTCACGGAAAACGGGCCGTC
GCCGAGATACAACGTGACGATACTTTGCAATAGAGTAATAAATATCTAGAATGCTCAGTCTTTTGTCTCGCGCTG
CATAATAGTTGCACATATATTCTTCCTCTTCTCGCTTTCCTCTCCTCTCGCTGACACTCTTTTGGCGTTCTCTGA
TAATACGCTGTCGATATCCTTTCATATCGAGCTCGACACGACCTTTTCTACTGATGAATTCCATTATTATGAGTT
ACTATATCATTTCCGAATGCTGAATTAGAAATAATCTTCGCGCTTAGTTGCGTACTAAATAAGCTGTTTCCATAA
AGGCCCTGCTAAATATTTTTTAAATATCAATTCAAATGTCAAAGTGATTTGCATAATTAGAGTTACATAAAAAGG
TTGAAGTTTGAAATTTAATTCGCAAGAGTCATACAATTCCCATTATAAAACTTTGAAAACAGAGAGAAAGGGAGA
CGAAGTTGTTTTAAATTTCTTCGAAAACTTTTTACTTATCTCGCTTTATGTATCAAGAACAATAAAGAAAAGGAG
TTAACTTCATCTTCATAAGAGAATATACATATCTATACGTATCCTTGATAAAACCTCTTCTACGCAGTCTAACGA
AAGCTCTCAAGACACCGGATACTTTTAAAGGGTACATAAAACGCGATACTCCGCAATAATTGATGTTCTCTACCT
TTATCGGAAAATAAAGCGGAGGAATTGCATTTCAGCTACCATCGAGGATCCAGTGAACGATGAGATGCCGTCGGA
TCTGTCGCCCATCCGAAACCGGCGCGTGACCTGCGACCTTCTGTCCTGGCAGTCCAAGTGGTTAAGTATCAATCA
```

```
CAGTGCCTGCGCAGCTAGATGCCTGTCGCAACGACGCAAAGGTGGCCGCTGCCGCGACGGTATCTGCGTCTGCAG
AAATTAG
//
Gene 1
Gene 339 3042
  Exon 339 435 phase 0
  Exon 2849 3042 phase 1
//
FT          CDS    join(339..435,2849..3042)
FT                   /note="Match to DEF2_APIME_Defensin-2"
//
>Atta_cephalotes_contig21145,.[339:3042].sp.tr
LFAILVIFVVLACTSVSTLPAVYDGPTYELTTIEDPVNDEMPSDLSPIRNRRVTCDLLSW
QSKWLSINHSACAARCLSQRRKGGRCRDGICVCRN
//
>Atta_cephalotes_contig21145,.[339:3042].sp
ATGAAGCTGTTCGCTATCCTCGTTATCTTCGTCGTATTGGCTTGTACCTCAGTCAGCACA
CTACCGGCTGTCTATGACGGACCTACTTATGAACTGACTACCATCGAGGATCCAGTGAAC
GATGAGATGCCGTCGGATCTGTCGCCCATCCGAAACCGGCGCGTGACCTGCGACCTTCTG
TCCTGGCAGTCCAAGTGGTTAAGTATCAATCACAGTGCCTGCGCAGCTAGATGCCTGTCG
CAACGACGCAAAGGTGGCCGCTGCCGCGACGGTATCTGCGTCTGCAGAAAT
//



>Hs_defensin-1 [organism= Harpegnathos saltator] putative precursor protein
(complete)
MKLLAIFALFCVLAYASASALPAVYDGPIYELTPIEEAASDNTPRDMAEEVPIRQRRVTCDLLSWTSKWFSFNNS
ACAAKCLVQRRRGGSCSGGVCVCRG*
>Hs_defensin-1 [organism= Harpegnathos saltator]
ATGAAGCTGCTCGCTATCTTCGCCCTCTTCTGCGTGTTGGCGTACGCCTCGGCGAGCGCGTTGCCTGCCGTCTAC
GATGGACCGATTTATGAGCTGAGTAAGCATATTTTGTCGATTCGGAATTCACCGCGCGATGCGTCCGCTTCTTA
CACGTCCGCGACGATCGGTCATCGAGCTTTCGCCCGGGCCGGCATTCAGAGTCCGATTTTGCACCTGAGAACTGA
GAGTCTTGAGCTCCGCTTGAGGTGGCACGAAGCTCGCCTGATCGACCCGTCGCGCAAATAGTCGTCGCTTGAAAC
TGCTCTCCGATGAGACACTCTGGTCGGAATGTAGAGTCGGATCTGGGTGAATGTTTCGATTGGATCATACGATAC
ACGCGACGTTGTGCTTGTAATCGCGATTCAACTGCCAGAGTCACATCTTGAGATTCCAATCCGACTGTCTGAATC
TCGAGGCAAGTTTCGAGTAACGACTTAGTTGACACGCGAGAGAGCCTCGGCGTCTCGAGAACGGACCGGGGGAAG
TCCCGGCTATCAGATCGGACGGCGAGAATGTCGCCGTCAGGTTCCTCGCGAAAATTCCCCGTGGTTAGGCGACTA
CGTGCGATACGTCCCGATACTCTTCATCTCGACACGTCACTTCGGGATACAGCGCCGGGGAAGTCCTCGATGGAG
TAATAAATATCCAGAGTACACGCTACAGAATAGCCCCGTCACTCCGATTCTACTCCACGCTTCTGGCATGCTCCG
ATAATACGCTGTCGATATCCCCTCACGTCGTAGTCCGACGCGTCCTTCTGCTCCGTCGAGAACTATTTCGTTATT
ATTACAGCGAGTTACGCGTCGAGTCGATCGCAGCTTCCCCCCGTCGACGCGCGTGAATCCTCCGCGGTGAGAAGG
AAAAAATTGTATCGGTATTTGCCGACTATAATTGCCCCGCGAGTGAAATATTAGGCGTACAGCTTAATTCTCGGC
CCCAAAATTCAATGAATGACAGCTTCTATTCAAATTGACATTTATTCCCGCCCTATGGTGGTCTGAGAGTACTAA
GTCACAGCTTTGCAATATGCAGTATAACAGATGGCAGCCCTGTTGATAAAGGGTGTCCCAAAATTCACGCAAGAA
GTAATTTTGATAGAAAACACAGGTTTATAATTAAAAATTGTAAATTTTTTATTGATTCATAAAGTATAGGGTTAT
GTATGGAATAGCATATCGGGTAAATGGCCTCCACGGCTTTGCTGGCACATATGCGCTCTTTTGTTGAAATTTTCC
ATGACCGTTTTGCATAAATGTGGCTAAATTTCGTTGATACAGCGCTCAATTTCCTTCTTCAAGGCGTGGGTGTCG
TGGGTTTGTTGGTATAAATCTTAGACTTCAAAAGAAGAAACCCCAAAGAAAAAAGTACAACGGCGTTAAATCGCA
TGATCTGATCACCAAAACGGGAAATTACACGACCAGAAAATGACTCATGCAGTAATTGAATTGTTTCTCGGGCTG
TATGGCATGTGGCACCGTCGTCTTGTTGAAAGCACATGTCGTCCACATCCATATCTTGCAATTTAGGCACAAAAA
ACTGGATTATCATGTCGCGATAGCGAGCACCATTAAATAGTTATTGCCTGACCAACCGCATTTTCGAAGAAGAAT
GGTCCGATGATGCCCCCAGCCGCCAAAATCCGCACCAAACAGTGACACGCTGTGGATGCATTTGATCTTCTGAAC
CCCAAATGCGACAATTTTGTCGATTAACCAAGCCATCGAGATGAAAATGAGCCTCATCGCTGAAGATGATTTTGT
TCGTAAAATCAGCACTTGTTGTTGTTCCATAATCCATTCAACGAACTCTCTTCGCTGTGCGTGGTCAGTTTAAAA
ACCCTGAACCCTGTCAGCTGTCACGCTGTCTTTTTTATTCGTTGGCAACACTTTGCCGCACAAATGGCGCAAAAT
TCAAATCTTGCGTGAATTTTGGGACACCTTTTGTAACCTCAAAGACAGTAGTGAATGCAATGTCGTTCGAAAAGC
AACATTTGAGGCACTGCCTCCTTTTCGCTTTCCAACTTAAAAAAAATGCAGCTGAAGCGCAGGAAGTGATTTGTT
GTTCGGCTTTGGGCGAAAATGCTGTTTTCCACAGCACTTGCAAGAAGTGGTTTCAGAGGTTTCGAGAAGGAAATT
CCGGTCTTGAAGACGAACAACGACTGGATCAGCCAAGGAAGGATGTTGCGTACTACGAATTGCTTGAACCCGGTC
AAACGCTCAATGCTGAACGCTACAAGCAACAACTAATCAAAGTGAATGACGCACTCGAGCAAAAAAGACCATTTA
CTGGTAGCGGAACGAGGCAGGTATCACTGCTGCATTATAACGCACGGCCACACACGGCAAAAGCGACCCTGGAAA
TCATCTCCGACTCAGACTGGAAAATCCTTCCTCACGCGACGAATTCTTCAGACCTCGCCTCATCAGAGATCATCA
CCTGTTCCGATCGCTACATCATCACCTAGCTGATTCGCACTTCCGAACTTCTGAAGAAGTCAGAAAAAGCACCGA
TGACTTCATTGAGTCCAAACCACCGTCGTTTTTCCAAAGCGGAATTCGAAAGTTGCCTGGAAGGTTGCAGAAATG
TGTAGAAAGTGAGGGAGATTACTTTCAGGATTAGGTCCTTTATTATCCTTTCTTTTGAAACTGAGCATTGAAGTT
GAGCAAAAAGGCCGAGAATTAAGTTGTACACCTAATAATTGCACTCGCCGATCATTTCTTTCGTAGCGCGCGCTA
```

```
CGAAAGTCGGTGCTGCGTGAGAAAATAGTGTTACCGATGATAAGTTTATTGTGCCGCTGCTATATTTCTCTCGCT
GGAATTGGAGCGATCGGAAGAGATACGACTGACGGCAGAAATAACTGAAATGTGTAGCGAGATCGGTGATTCTTT
CTTTCTCACAACGTACGAACGCTTCGCTGTCCAGCGGAATATCCGCGTGACGTAAGAATCTCGCGGTCGCGCACG
TCGCGAGACTCGCCCCCGCGAAAAATTTCCCGGCTAAATTTTCCGTGCCTTCTTCCGACGGTAAACCGATGAGAC
ATCTTCTACGAATTCCACGAATATTGGCGAAACGTTGGCGAAGGATCGTACTTGTCTCGTAAATGTTCTCGGCTG
AGATCCCACGTGAAAGCCAAAGCAACAAACAACCTACGTAGTCGCGCTCGGGTCCGAGTCCGAGTGGCTTTCCAA
CTCGCGCAGAATAAATTCTCCGCGCTGAATCGTCGGGTGAATATCTCCCCTTGGAACTGGAGGCCGGAATCTGAC
TGACATGTGCTCCCCGATCATCGGAATGAATCCGCTCTGATTGCGCGATTGAAACATTCATTTGTTCATGACTTT
AAAAACCTCGTCGATTTGAGTGGAACACACGCACTCTGCCGATTTCAAGTGACAGCGTCGAGTGATCTTCAGCAT
CGAGCTTCGAGCGAATTTTCATTCGTTCCCGGTGAGAATCACTCCACTTGAAGAATTTCGAAGGGGAATCGATTT
TAACGCAACGCGTCCTTGCCCCGATTATTCCGTCTATCCGACCCTCTAATTCCAGCGAGATATTTCACCTGAGGA
GACTCAGAGAGGAGGCAACAAGACAACGTCATTCGTATCGTGTTCGGAAGCCTAGACAAGAGAGACCTTCTTGCG
CCTCGTCTTGTTGTCCGTGCAAGAAGCACAGGTCCAACGAGCCGTGTTAAGGGTTCCAAACTCGGTTCGAAAATC
GAACCGAACTACTGTGATGAAAAAGTATCCGAAGTTTATTTTTAAAAATCAAAATTACAGTTCAATCATTCATGA
ACATTCTTATAGTTTCCTTCAAAGTAGTCCCCATTGCTTGCAACGCACATATGCCAGCGTTTGATCCAATCCTGG
AAACATCGCTCATACATCGCGGCGGACGCGGTATGGCCTTCAATTCACGCAGCGATTTTTCTCTCATGTCCTCAA
TCGATTCGAAACCCGCTGTCCGCGAAGTGGTTTTTTCAGTAGGGGGAACAGGAAAAAGTCGCAGGGGGCCATGTC
TGGTGAATACGGTGCTTGCGCAGCGACATTCGGTGAGGTTTTTTGTCAGAAATTCACGCACAAATGTCGAGTGGT
ATGCGATCAGGTGCAAAAGTTGTCGCGATGCAAAATCCACGAGTTGTTTTCCCACAATTCCAGTCGTTTCTTATG
GATTTTTTGCTGGCACGATCCAAATGATACTCCAACTTCATCTGCTATCTCTCGCACAGTCAATCGACAATTTTG
GAGCACCGATTCTTTCACTTTCTCAATGTGAGCTTCATGCGTGACGTTGATGGTCTTCCAGAGCGTGCTGCGTGG
GTCGTCTTCAACGGATTCTCGTCCTTCTTGAAACTTCTTAAACCACTCAAACGTACTTGTTTTCGATAAACAATC
ATCGCGCCATAAGCTTTCTCATGCAACATTTTCAACGCGTTTGTGAACGAAATTGCGCTGGCAACACAAAATTTC
AAACAAATTCTTTGCTCCATATTTTCCGACATCACAAAAATCGCCGTGCACAACAATCTTCACTGACACAAACAA
CTCCCACTTTCAACTGCGGCTTGAAGGAACGCCATGATTTTTTCACCAAATGTGAAGGACATGCGTACCAATTTG
AGAAAGCAAACGGATTTGAGTCACAGTTTACCCGCGATTTTTAAAAAATAAAGTTCGCATACTTTCTGATCACCT
GATAAGTAAACTTCGTTACGCGCAAACGCCTCGCTTCGGACTGATGAATGAAAAAGAGATCGCGTTTTGCTTCAG
CCCCCATTGAGGAAGCGGCGTCCGACAACACGCCACGGGACATGGCAGAGGAAGTGCCCATCCGACAGCGTCGGG
TCACCTGCGACCTCCTCTCTTGGACGAGCAAGTGGTTCAGCTTCAACAACAGTGCCTGCGCGGCAAAGTGCTTAG
TGCAACGTCGTCGGGGCGGTTCTTGCAGTGGCGGTGTCTGCGTCTGCAGGGGTTAA
//
Gene 1
Gene 1252 6253
  Exon 1252 1348 phase 0
  Exon 6051 6253 phase 1
//
FT          CDS    join(1252..1348,6051..6253)
FT                   /note="Match to DEF2_APIME_Defensin-2"
//
>HarSal_7503.[1252:6253].sp.tr
MKLLAIFALFCVLAYASASALPAVYDGPIYELTPIEEAASDNTPRDMAEEVPIRQRRVTC
DLLSWTSKWFSFNNSACAAKCLVQRRRGGSCSGGVCVCRG
//
>HarSal_7503.[1252:6253].sp
ATGAAGCTGCTCGCTATCTTCGCCCTCTTCTGCGTGTTGGCGTACGCCTCGGCGAGCGCG
TTGCCTGCCGTCTACGATGGACCGATTTATGAGCTGACCCCCATTGAGGAAGCGGCGTCC
GACAACACGCCACGGGACATGGCAGAGGAAGTGCCCATCCGACAGCGTCGGGTCACCTGC
GACCTCCTCTCTTGGACGAGCAAGTGGTTCAGCTTCAACAACAGTGCCTGCGCGGCAAAG
TGCTTAGTGCAACGTCGTCGGGGCGGTTCTTGCAGTGGCGGTGTCTGCGTCTGCAGGGGT
//
```

```
>Hs_defensin-1a [organism= Harpegnathos saltator] putative precursor
protein (complete)
MKKRSRFASAPIEEAASDNTPRDMAEEVPIRQRRVTCDLLSWTSKWFSFNNSACAAKCLVQRRRGGSCSGGVCVC
RG*
>Hs_defensin-1a [organism= Harpegnathos saltator]
ATGAAAAAGAGATCGCGTTTTGCTTCAGCCCCCATTGAGGAAGCGGCGTCCGACAACACGCCACGGGACATGGCA
GAGGAAGTGCCCATCCGACAGCGTCGGGTCACCTGCGACCTCCTCTCTTGGACGAGCAAGTGGTTCAGCTTCAAC
AACAGTGCCTGCGCGGCAAAGTGCTTAGTGCAACGTCGTCGGGGCGGTTCTTGCAGTGGCGGTGTCTGCGTCTGC
AGGGGTTAA
```

```
>Hs_defensin-2 [organism= Harpegnathos saltator] putative precursor protein
(partial)
SLSNYLFAGDNTRKSSKLLLDRNVHLRARGATCDVLGAFKHWTTPLASRRCIARERSGGSRNSQYVCVCCK*
```

>Hs_defensin-2 [organism= Harpegnathos saltator] (partial)
TCTCTGTCTAACTATTTGTTTGCAGGTGACAACACACGGAAGTCATCTAAGTTACTTCTCGACAGAAACGTTCAT
CTCCGTGCACGTGGGGCCACTTGCGACGTCCTCGGTGCTTTTAAGCACTGGACCACTCCGCTTGCGTCGCGGCGC
TGCATAGCTCGAGAAAGGTCCGGAGGCAGCCGCAACAGCCAATATGTCTGCGTGTGCTGCAAGTGA

Diuretic hormone-like

>Cf_diuretic-hormone-like [organism= Camponotus floridanus] putative precursor protein (partial)
CIIPSLLLYVIFAIKVNRSIQFCARSHESYWDQQDDIDRDEFLELLSRLSRTVMNRPEMENSKRGLDLGLSRGFS
GSQAAKHLMGLAAANYAGGPGRRRRSEQA*
>Cf_diuretic-hormone-like [organism= Camponotus floridanus] (partial)
TGTATAATACCAAGCCTGTTGCTATATGTCATTTTCGCAATCAAAGTTAACCGATCGATCCAATTTTGCGCACGC
AGCCATGAAAGTTACTGGGATCAACAGGATGACATAGACCGAGACGAGTTTTTAGAGCTACTTTCTCGTCTAAGT
CGCACCGTTATGAACCGTCCCGAAATGGAAAACAGCAAACGAGGATTGGATCTGGGTCTCAGTCGTGGCTTCAGC
GGTTCTCAAGCGGCGAAACATTTGATGGGACTCGCGGCGGCGAATTATGCCGGCGGTCCCGGACGGAGGCGTCGC
TCGGAACAGGCGTAA

>Ac_diuretic-hormone-like [organism= Atta cephalotes] putative precursor protein (partial)
RSTKRGLDLGLNRGYSGSQAAKHMMGLAAANYAGGPGRRRRSEQAVIRCLLLCAVFTINRSIPQSILLHYARSHE
SYWDQQDDIDRDEFLEILSRLSRFNRPEME
>Ac_diuretic-hormone-like [organism= Atta cephalotes] (partial)
CGCTTGTTCCGAGCGACGCCTTCGTCCGGGACCGCCGGCGTAATTCGCTGCCGCGAGTCCCATCATGTGTTTCGC
CGCCTGAGAACCGCTGTAGCCACGATTGAGACCCAGATCCAATCCTCGTTTGGTACTGCGAACTTCCATCTCAGG
ACGGTTGAAGCGACTAAGACGAGAGAGTATCTCTAGAAACTCGTCTCGGTCTATGTCATCTTGCTGATCCCAATA
ACTTTCATGGCTGCGTGCGTAGTGTAAAAGAATTGATTGAGGAATTGATCGATTAATCGTGAAAACTGCACACAA
TAATAAACATCGAATTAC

>Hs_diuretic-hormone-like [organism= Harpegnathos saltator] putative precursor protein (partial)
NRTFSRSTKRGLDLGLSRGFSGSQSAKHMMGLAAANYAGGPGRRRRSEQAHESYWDQQDDIDRDEFLEILSRLSR
TVMSHPEMEK
>Hs_diuretic-hormone-like [organism= Harpegnathos saltator] (partial)
CGCCTGTTCCGAGCGACGCCTCCGTCCGGGACCGCCGGCGTAGTTCGCTGCTGCGAGCCCCATCATGTGTTTCGC
CGATTGAGAACCGCTGAAGCCGCGACTTAGACCCAAATCCAATCCTCGCTTGGTGCTGCGAGAAAACGTGCGGTT
TACTTTTCCATTTCAGGATGGCTCATTACGGTGCGACTGAGACGAGAGAGTATCTCTAGAAACTCGTCCCGGTCT
ATGTCGTCTTGCTGATCCCAGTAACTTTCATG

Abaecin-like

>Cf_abaecin-like [organism= Camponotus floridanus] putative precursor protein (partial)
VKFYLSIFVLLIAMAVLACSPIRQSPGGWKPISSFPSHEPFNFLLNF
>Cf_abaecin-like [organism= Camponotus floridanus] (partial)
GTGAAATTTTATTTGTCTATTTTTGTGCTCTTGATAGCAATGGCGGTTCTTGCGTGCTCTCCAATCAGACAATCT
CCAGGTGGTTGGAAACCAATTTCGTCTTTCCCCAGTCACGAACCGTTTAACTTCTTATTAAATTTC

>Ac_abaecin-like [organism= Atta cephalotes] putative precursor protein (complete)
MKFHLFIFTLLLAVMTVFAYQSPVRKPPPGGWKPFPTFPGQGPYNPKIRFPH*
>Ac_abaecin-like [organism= Atta cephalotes]
ATGAAATTTCACTTGTTTATCTTCACTCTGCTCTTGGCAGTAATGACAGTCTTTGCGTATCAATCTCCGGTCAGA
AAACCGCCTCCAGGTGGATGGAAGCCTTTCCCGACTTTTCCTGGTCAAGGACCATATAATCCGAAGATCAGATTT
CCTCATTGA

>Hs_abaecin-like [organism= Harpegnathos saltator] putative precursor protein (complete)

MKFHLFIFTLLLAVMAALAFRVPPQQPPPGGWKKFPTFPGQGPFNPKIGK*
>Hs_abaecin-like [organism= Harpegnathos saltator]
ATGAAATTCCACTTGTTCATCTTTACTCTGCTCTTGGCAGTGATGGCCGCTCTTGCGTTCAGGGTTCCGCCTCAA
CAACCGCCTCCAGGCGGCTGGAAAAAATTCCCAACTTTTCCCGGTCAAGGACCCTTCAACCCAAAGATCGGGAAG
TGA

Eclosion hormone-like

>Ac_eclosion-hormone-like [organism= Atta cephalotes] putative precursor protein (partial)
IGVCIRNCAQCRKMFGVYFMGQKCADFCMKYKGKLIPDCEDEYSIRPFLQVAEYDY*
>Ac_eclosion-hormone-like [organism= Atta cephalotes] (partial)
ATAGGTGTTTGCATACGAAACTGCGCGCAATGCAGAAAAATGTTCGGCGTGTATTTCATGGGTCAGAAGTGCGCG
GACTTCTGCATGAAGTATAAGGGAAAGCTCATTCCAGATTGCGAGGACGAGTACTCCATTCGTCCTTTTCTTCAA
GTAGCAGAGTATGATTATTAA

Neuroparsin-like

>Ac_neuroparsin-like [organism= Atta cephalotes] putative precursor protein (partial)
GPGHICGGPSDSWGVCGDGLICSCNRCTGCSVDNLTCF
>Ac_neuroparsin-like [organism= Atta cephalotes] (partial)
GGCCCCGGACATATTTGTGGTGGTCCAAGCGATTCTTGGGGAGTGTGCGGCGACGGATTGATCTGCAGTTGCAAT
CGCTGCACTGGTTGCAGCGTGGACAACCTTACGTGTTTC

Allatostatin

>Cf_allatostatin [organism= Camponotus floridanus] putative precursor protein (complete)
MKTSSLIAMRLIIFYLLSVVGRSTAAVEEAPASSLHIPRLNPLSSNLEYDEPSEKRAYAYISEYKRLPLYNFGIG
KRWIDNSEDKRTRPFSFGIGKRLRDYRFGIGKRNSGYRPLGMDFSVDNMDFHSREDNLDDFIDDKRGGQPFSFGI
GKRGWKLPMGEMAVSGRRLNDVVGPKYLLGLGKGLSENENLIQ*
>Cf_allatostatin [organism= Camponotus floridanus]
ATGAAAACTTCGAGTCTAATCGCTATGAGACTCATTATCTTCTACCTGTTGAGCGTCGTTGGACGATCAACAGCG
GCGGTAGAGGAGGCACCGGCCTCGTCCTTGCATATTCCACGATTGAATCCGTTATCGAGCAACTTGGAGTACGAT
GAGCCCTCTGAAAAAAGGGCGTACGCTTACATTTCCGAATACAAGAGGCTACCTCTTTACAACTTCGGCATCGGA
AAGCGATGGATCGACAATAGCGAGGATAAAGTGAGTTCCGGATGAAAATAAAATGTTCAATTTCTCGGCGCGAAT
AATTGCTTCGTTATAAGATTGGAAGATCTGGAATTAGAGAGAAATTAAATATCAGATCAAATATATTTTCAAGAA
ATATCTTTGCTAAATTTCTTTTTTATTTAAATTGATAAATAATACGGTTCTATTTAACAACATGATAATTAAGAA
ACGAGTAGTAAGAGCCTAAAAAACGCGGATGGCAATGCGTAAATTTTTTCGAACATTAATTGTTCGTGAAAAAAT
TTTTAAGACATATACATATATGCCAATATAGTTATAGATTCTTTTTTATTCTTTTTTATTGCTTTATTAAGTTAT
TTAAAATTTCCCAATGTCGAATAGTGCATAAGAATAAAATGTGTGATGCGACGAATAGAAATGAAATTAATTTGT
CATAATCATACTTGTCGCCCTCATATTTTTCTTTGCATTGAAACTTTATTTGCAGCGAACGCGGCCGTTCTCGTT
CGGTATCGGAAAACGTCTTAGGGACTACAGGTTCGGCATAGGAAAGCGTAATAGCGGATACCGTCCCTTGGGTAT
GGATTTCTCGGTCGACAACATGGACTTTCATTCTCGCGAGGATAACCTGGACGACTTTATAGACGACAAGCGCGG
CGGTCAGCCTTTCAGTTTCGGCATCGGAAAACGAGGCTGGAAGCTGCCAATGGGCGAAATGGCCGTATCCGGAAG
GAGACTAAACGACGTTGTCGGCCCGAAATATCTGCTCGGTTTGGGCAAAGGACTAAGCGAGAACGAAAATCTGAT
TCAATAA

```
//
Gene 1
Gene 17753 18806
  Exon 17753 18007 phase 0
  Exon 18483 18806 phase 0
//
FT          CDS    join(17753..18007,18483..18806)
FT                   /note="Match to ALLS_APIME_Allatostatins"
//
>CamFlo_contig5982.[17753:18806].sp.tr
MKTSSLIAMRLIIFYLLSVVGRSTAAVEEAPASSLHIPRLNPLSSNLEYDEPSEKRAYAY
ISEYKRLPLYNFGIGKRWIDNSEDKRTRPFSFGIGKRLRDYRFGIGKRNSGYRPLGMDFS
VDNMDFHSREDNLDDFIDDKRGGQPFSFGIGKRGWKLPMGEMAVSGRRLNDVVGPKYLLG
LGKGLSENENLIQ
//
```

```
>CamFlo_contig5982.[17753:18806].sp
ATGAAAACTTCGAGTCTAATCGCTATGAGACTCATTATCTTCTACCTGTTGAGCGTCGTT
GGACGATCAACAGCGGCGGTAGAGGAGGCACCGGCCTCGTCCTTGCATATTCCACGATTG
AATCCGTTATCGAGCAACTTGGAGTACGATGAGCCCTCTGAAAAAAGGGCGTACGCTTAC
ATTTCCGAATACAAGAGGCTACCTCTTTACAACTTCGGCATCGGAAAGCGATGGATCGAC
AATAGCGAGGATAAACGAACGCGGCCGTTCTCGTTCGGTATCGGAAAACGTCTTAGGGAC
TACAGGTTCGGCATAGGAAAGCGTAATAGCGGATACCGTCCCTTGGGTATGGATTTCTCG
GTCGACAACATGGACTTTCATTCTCGCGAGGATAACCTGGACGACTTTATAGACGACAAG
CGCGGCGGTCAGCCTTTCAGTTTCGGCATCGGAAAACGAGGCTGGAAGCTGCCAATGGGC
GAAATGGCCGTATCCGGAAGGAGACTAAACGACGTTGTCGGCCCGAAATATCTGCTCGGT
TTGGGCAAAGGACTAAGCGAGAACGAAAATCTGATTCAA
//
```

```
>Hs_allatostatin [organism= Harpegnathos saltator] putative precursor
protein (complete)
MRPTSTSLTAMRLIMLCLLSVVGESTAAMEDMPSSSLHMPRLNPLLNHVEYEEPSEKRSYAYVSEYKRLPLYNFG
IGKRWVDDNEDKRTRPFSFGIGKRLRDYRFGIGKRNSHPLNLDYLPADNLEAYHSREDNADDLMEEKRSNQPFSF
GIGKRGWKLAGATARRPNADVVAAPRYLLSLGKGIGEDEEMAQ*
>Hs_allatostatin [organism= Harpegnathos saltator]
ATGAGACCAACATCAACGAGTCTGACTGCCATGAGGCTCATCATGCTCTGTCTGTTGAGCGTGGTTGGTGAATCG
ACGGCGGCGATGGAGGATATGCCGTCTTCATCTCTGCATATGCCGCGATTAAATCCATTGTTGAACCATGTTGAG
TACGAGGAGCCTTCTGAGAAGAGGTCGTACGCTTACGTTTCCGAGTACAAGAGGCTACCACTGTACAACTTCGGC
ATCGGAAAGCGATGGGTCGACGACAACGAGGATAAAGTGAGTTGCGGGCGGATAAAGCCGTCTCACCCTTCTTCA
TTAAGAAATCTCTTTGCTTTGCTGGTGGCAAACGTCTCGCTCTCGAATGTGTGCGAAAGAATATTAAATAAAATT
GATGCCCGATAGAATAGCATGATGCAAAAACAATGTATAAATTTGTGTATTATTGTTTGAAGACTGAACCAAAAG
ATGAACGTTTAATTGAATCAAATTAAATCAAATCAAAAAGTCTAACTAAATCGAACTAAATTAAAGGATTCCGGT
CGATCGGCAAAGTGATAATATTGAAATGTATATAATTTCTTTCTCCCCCTTTTTCTATACATTAAGTTGTCCCAT
TTTAAATGAGCCAGGCAAAAATGTCGAAAAATATGAAAGATAATACGAAAGGTTTCAGACAAAAGTTGTATGGTT
TCAAAGGGGATATAAAACAACTATCATTTTGTTTACAGATGCCGTTACTTTTTAAAATTTCCAAAAGTCTATAAG
TTAATAAAAGTTAAGGCATTATTGTAACAATATATTCTGGAGACTAAATAAAGTAAAAGAGTCAAGTACTATTTT
TTTTTCGACAAAGAATTAGCGAGATATCGTAAATTAAATGTTGAAAAAGTTACCAAGTTTTCGCAAATATCTAAA
AAAATATTAGGAAAATAAAAAAATATTTCAGACAAAAATTGTTCAAATTAATTGGTAGAAAATAATGATATAATA
AAATGATATGATATAATAAAAATTGGAGTTTCCATTTAAGTAATGAAATCTGAAACATTTTTCGGTTTAACGCAG
TGTTTCTGAGATATTTGAGTGGATCCTTTAAAATGCCTGGTCCTTTAAAATATATCGCTGAAATTGCGCGCCAGG
CGACGCAGTTCACTAAATCGAGCGAATTCCCTTGCGAGCGAGCAGTTATTATGCGCGATCGAAACGTACCTCAAA
TTTTCCGGGTAAGTGAAGTAATTCTCTGTCCATTCCGAATTACAGGAATTTGAAAATCAATCTCAATCTCGGACT
GACACGAAAGTTTGTAAGAACATTTGCCAGTTTCGAGATATTCACTGATCATTACTTTTTTTAAATGGAAACTCC
AATTTTTTTATTATATCATCCTTTTTTATGAATCAATTTGAACAACTTTTGTTTGAGATATTTTTTTATTTGCTT
AGTATTTTTTTAGATATTTAATGAAAATTTGATAACTTTTTTAACATTCAATTTACGATATCTCGCTAATTATTT
GTCGAAAGAAGAAATAGTATTAGAATTTTTTACTCTAGTTAGTCTTGAGAATATGCTATTAGAATAGTGCCCTAA
CTTTTGTTAACTTATCAACCTTTGGAAATTTCAAAATCATCAAATTAACAAAAGTCGTAGAGAGAGGAAAAATAT
CGTAGAGGAAGAACGATATTCCCGAGAGCATTTTAAAGCCAGAAACCGCAGCTTTCATATATTGTCAAGCTCAGT
GTCGCATTTGCTCCAGGCGGCACTTCTTCCAAGTCATCTATGAGAAATTGCCGGGTAAATGTCGACTTTGAGGTC
ACACGTACACAAAGGATGAATCATTTTGTTTTATTCTGTTTAAGACAACTTGAGAACTTAAAGTCTATTTATTTT
GCCAAAATATCCGTTTTGAAATTAAACTACTATTTTTTCCGCCAAAACTTTTGTCTGAAACATTTTTCCATATTT
AAAGCCGTTTCCGAGATCCAGTGGTGGAGTCGGACTTTACAGATATCCTGTATAGAGCGATCCACATAAAGTGTA
ATAACCCAAAAACTAAAAATATTTGTCGTATTAAAAAGTTATTCATGAGAAAATTTCATATATATCGTCTTTCTA
TATAAATAAAATTTTGCCCCTCCTTCTTCTTGAAGGAAAGTGGGAAATAACTTCGTAATTTTAAATACCACTTCC
TATAAAATGTTACATATTCGGATTGTGTGAGAAAAAAGAAATAAACTTTATCTGGAACATTTCTTTAAAAATTGC
TTCTATATAAAATTATAAACATTAAGTTTTAAAATAGAATGTATTCAAACTAATGTGATAAAATCATAAATTCAA
TAAAAGGTTACATAGTTCTTCTGACTACATAGTTTTTAGCTTTTAATAGTTAATAAGTTTTAACATAGCTTTTTT
TACTTTATTACATGTTTAAACTATTAATCATCCACATTTCATAAATACTTATTAATTCTTTCTACAAAATTTTGT
CTAACTCTTTCATAACTTTTCTAAAGTTTCATTTGAAATTACGAAATTCCTCCCCCCCTTCCCACTCTCTTCAAG
GCTGAATTTTTATTTATATAGTTAGAAAGACTTCATGTGAATACATGAAACTTTTTTATGAACAACTTTTTGGCA
CGATAAATATTTTTAGAGTTTTTTAGGATTGTCACACTTTACGTGTAGGCCACCCTGTATAATAATTTTTCTCCG
GATATTCCGTGCACCAATTACAAATAAACTCGCATACCGTCTTCTCGCGAGTTTTGTTGAATAACACACAATTAA
TTAGCATTAAATAACATACATAATTAATTAACATTGAAATAATACATGCTGAATTAAAAATTCAAGAAACACACC
GCGTCGGAAGCGAAGTATTCTCCTATATAGCACAAAAGTTGCAGCAATATTGTTGCAACGTTGCAGCGATGTTGC
TGCAACTTCTCTACTGTATAGGTTAGGTATAGACTGATATGTTGCAGTCAACGTGTAGCCAATGTATCGTACAAT
AGTCCTGGAACACTCTGTATATCCTAATTGTGTATGTGTGTGTGTGTGTGTGTGTGTGTGTGTGTGTATGTGTGC
GCACGCGTGATACGATGTATCATGCAATGAGAATTATCCGCACACGTACAGTCGCATAATGCAATTATGAAACCT
TCACACGTCCTAATAATTCCAATTCGCAGAGAACACGGCCCTTCTCTTTCGGCATCGGCAAGCGTCTGCGAGACT
ACAGGTTCGGCATAGGCAAGCGCAACAGCCACCCCCTCAACCTGGATTACCTTCCGGCCGACAACCTCGAGGCTT
```

```
ATCACTCTCGCGAGGACAACGCGGACGATCTCATGGAGGAGAAGCGCAGTAACCAGCCGTTCAGCTTCGGCATCG
GGAAACGCGGCTGGAAGTTGGCCGGAGCGACAGCGAGGAGACCCAACGCCGACGTCGTCGCTGCTCCGCGTTATT
TACTGAGCTTGGGCAAAGGCATAGGCGAAGACGAGGAGATGGCTCAGTAG
//
Gene 1
Gene 83921 87417
  Exon 83921 84181 phase 0
  Exon 87100 87417 phase 0
//
FT          CDS    join(83921..84181,87100..87417)
FT                   /note="Match to ALLS_APIME_Allatostatins"
//
>HarSal_contig8088.[83921:87417].sp.tr
TSLTAMRLIMLCLLSVVGESTAAMEDMPSSSLHMPRLNPLLNHVEYEEPSEKRSYAYVSE
YKRLPLYNFGIGKRWVDDNEDKRTRPFSFGIGKRLRDYRFGIGKRNSHPLNLDYLPADNL
EAYHSREDNADDLMEEKRSNQPFSFGIGKRGWKLAGATARRPNADVVAAPRYLLSLGKGI
GEDEE
//
>HarSal_contig8088.[83921:87417].sp
ATGAGACCAACATCAACGAGTCTGACTGCCATGAGGCTCATCATGCTCTGTCTGTTGAGC
GTGGTTGGTGAATCGACGGCGGCGATGGAGGATATGCCGTCTTCATCTCTGCATATGCCG
CGATTAAATCCATTGTTGAACCATGTTGAGTACGAGGAGCCTTCTGAGAAGAGGTCGTAC
GCTTACGTTTCCGAGTACAAGAGGCTACCACTGTACAACTTCGGCATCGGAAAGCGATGG
GTCGACGACAACGAGGATAAAGAACACGGCCCTTCTCTTTCGGCATCGGCAAGCGTCTG
CGAGACTACAGGTTCGGCATAGGCAAGCGCAACAGCCACCCCCTCAACCTGGATTACCTT
CCGGCCGACAACCTCGAGGCTTATCACTCTCGCGAGGACAACGCGGACGATCTCATGGAG
GAGAAGCGCAGTAACCAGCCGTTCAGCTTCGGCATCGGGAAACGCGGCTGGAAGTTGGCC
GGAGCGACAGCGAGGAGACCCAACGCCGACGTCGTCGCTGCTCCGCGTTATTTACTGAGC
TTGGGCAAAGGCATAGGCGAAGACGAGGAGATGGCTCAG
//
```

```
>Ac_allatostatin [organism= Atta cephalotes] putative precursor protein
(complete)
MKTATSLITIRIIMFYLLSVVGRSAVAIEEASSSSLHIQRLHPLLNNMEYNEEPMKKAYIAEYKRLPLYTFGIGK
RWIDNNEDKRTRQFSFGIGKRLRNYDFGIGKRNNEYHPLDYFLVDNMGNYQSHEDNSNDFIENKRGNHQFGFGIG
KRVWKLATGETAVSGRRLNDAIVPKYWFSTLTKELDENEDLNQ*
>Ac_allatostatin [organism= Atta cephalotes]
ATGAAGACAGCAACAAGTTTAATTACTATACGAATTATTATGTTCTATCTGTTGAGCGTTGTTGGACGATCAGCA
GTGGCAATAGAAGAAGCATCTTCCTCATCTTTACACATTCAACGATTGCATCCGTTATTAAACAACATGGAATAT
AATGAAGAACCTATGAAAAAGGCATACATTGCCGAATATAAGAGATTACCCCTTTATACTTTCGGTATTGGAAAA
CGATGGATTGACAATAATGAAGATAAAGTGAGTTTTAAATGAAATGAAATATCCTCTTTTAATGTTTAAAAATGA
ATGAATTTTTCTGTTACAAAAAAATTTAATTACAGAAATGTCATAAATCCAGCAAATATCCGAATAAATAGTGCA
ATTACAAAAATTGCGTAATAAATTTTTGTAATTAACAAACATGAACTTTTATTATGTTACTTGTACTTATACGAT
ACAATACTATTAAAAGTATTATAAGATTGTAACATATAAAATTCGATTATATTTTAATTAAGGTCAAAATTAATG
TTTCTTCAATGTAATGTTTTCACGTTTTCAATTTTAAATATATTGATTCGGACTTTAATAACATAAACTTTAACA
TATCAATGTTTTATTATACAAGTTATAATAATTAATTAACATACAGAAAAATAAAAACATCCACTAATTAATTTA
AATAAGAAATTAAATAATGGATATTTTTATTGTTCTGATAATATTTCTGATAATTGTATCTTTATGTGTTGAAAT
TATATCTTTATACATAGCGAACCCGGCAATTCTCGTTCGGTATTGGCAAGCGTCTCCGAAATTACGATTTCGGTA
TAGGAAAGCGCAACAATGAATATCATCCTTTGGACTATTTCTTGGTCGATAACATGGGGAACTATCAATCTCACG
AGGACAACTCAAATGATTTTATAGAAAACAAACGTGGTAACCATCAATTCGGCTTTGGAATTGGAAAACGAGTTT
GGAAATTGGCGACTGGAGAAACGGCTGTATCCGGAAGAAGATTAAACGATGCTATAGTCCCGAAATACTGGTTCA
GTACTTTGACCAAAGAACTAGATGAGAATGAGGATTTGAATCAGTAA
```

Inotocin

```
>Hs_inotocin (vasopressin/vasotocin-like) [organism= Harpegnathos saltator]
putative prepro-protein (complete)
MLRELVVFASLIFLSYACLITNCPRGGKRGDIIPSLGTVTRECPPCGPNHLGQCFGPHICCGPTIGCFIGTPETY
RCRKESPYARPCIAGYAMCRGNTARCATNGICCSQDSCHMDTSCRISDVVSNDRKMDADLSAILSSNEASHEIIQ
*
>Hs_ inotocin (vasopressin/vasotocin-like) [organism= Harpegnathos saltator]
ATGCTAAGGGAACTCGTCGTTTTTGCAAGTCTCATTTTCTTAAGTTATGCTTGTTTAATTACGAATTGTCCTCGC
GGTGGAAAAAGAGGTGACATTATACCTTCTTTGGGAACTGTCACTCGAGAAGTGAGTAATGTTCCAAATCATATA
ACATATCTTTTCTATAATTAAAAAATTGTTGCATTTATCTATTTTTTCCCGCATTGAAAAGAGTGTAGAATATTG
ACAAAATTCGTATTATTAGAAGAAAGTATTATCAGAGCAAAATTTCTGATCATTTTGTTAAATCAATCATTGTTC
ATCTAACCAATATCTTCGTGAATTTGTATTGATATATATTGTAATGTGCGATATGACTTTCTGATATAATCTAAT
CCTTATAATATTCTCTGAACTCAGTGCCCTCCATGTGGTCCCAATCATCTGGGTCAATGCTTTGGGCCTCACATT
```

```
TGCTGCGGCCCTACCATTGGATGCTTCATTGGAACACCAGAAACATACAGATGCAGAAAGGAAAGTCCATATGCT
AGACCTTGCATCGCAGGCTATGCAATGTGCCGTGGAAACACAGCTAGATGTGCTACAAATGGAATTTGTTGCTCA
CAAGGTAAATATAAACACTATATAACATTGAACATTTAAAAATAAACTTTGGAACAATCTCTCTCCAGAGATTTA
ATCTAATCAAATTTTTATGACTAATTTTATGACTAATGAGGGAAATCCTATTAGCTAAATATATATGATATAACA
ACAGAATGTTAAAACAGTATAGTAAAATTTATTATATAATAGAAACGCCCTCGAATGTAATGGTAATCAATTGCA
TAATGGTAAAAATAATTTGCAATTAGAAGTAAACCGTTTTGGAGATTACATTGTTGATGCCATCATGAAATATGA
GGAAGTTATGTTACAATAAAGATGTATTATTTTGTCCTAGACTCTTGCCACATGGATACATCGTGCAGAATTTCT
GATGTCGTTAGCAATGATCGCAAAATGGATGCTGATCTGAGTGCGATATTGTCGAGTAACGAAGCCTCCCATGAG
ATAATTCAATAA
//
Gene 1
Gene 31248 32174
  Exon 31248 31322 phase 0
  Exon 31596 31800 phase 0
  Exon 32137 32174 phase 1
//
FT          CDS    join(31248..31322,31596..31800,32137..32174)
FT                   /note="Match to A3RE83_TRICA_Arginine-vasopressin-like-peptide"
//
>HarSal_1385.[31248:32174].sp.tr
CLITNCPRGGKRGDIIPSLGTVTRECPPCGPNHLGQCFGPHICCGPTIGCFIGTPETYRC
RKESPYARPCIAGYAMCRGNTARCATNGICCSQDSCHMDTSCRISD
//
>HarSal_1385.[31248:32174].sp
TGTTTAATTACGAATTGTCCTCGCGGTGGAAAAAGAGGTGACATTATACCTTCTTTGGGA
ACTGTCACTCGAGAATGCCCTCCATGTGGTCCCAATCATCTGGGTCAATGCTTTGGGCCT
CACATTTGCTGCGGCCCTACCATTGGATGCTTCATTGGAACACCAGAAACATACAGATGC
AGAAAGGAAAGTCCATATGCTAGACCTTGCATCGCAGGCTATGCAATGTGCCGTGGAAAC
ACAGCTAGATGTGCTACAAATGGAATTTGTTGCTCACAAGACTCTTGCCACATGGATACA
TCGTGCAGAATTTCTGAT
//
```

```
>Cf_inotocin (vasopressin/vasotocin-like) [organism= Camponotus floridanus]
putative precursor protein (partial)
MLKQLVICASLIFLSHACLIVNCPRGGKRSDIASFLKTVTRE
>Cf_inotocin (vasopressin/vasotocin-like) [organism= Camponotus floridanus]
(partial)
ATGCTAAAGCAACTTGTCATTTGTGCGAGTTTGATTTTCTTGAGTCATGCTTGTTTAATAGTGAATTGTCCACGT
GGTGGAAAAAGGAGTGATATCGCGTCTTTTTTGAAAACTGTTACACGAGAAG
```

```
>Ac_inotocin (vasopressin/vasotocin-like) [organism= Atta cephalotes]
putative prepro-protein (partial)
MLKELIVFASLIFLSYACLITNCPRGGKRSDVASSLRTVIRECPSCGPNHLGQCFGPYICCGPSIGCFIGTPETF
RCRKESLYTRPCIAGYAMCRGNTARCASNGICCSQ
>Ac_inotocin (vasopressin/vasotocin-like) [organism= Atta cephalotes]
(partial)
ATGTTAAAGGAACTCATTGTTTTTGCGAGTTTGATTTTTCTGAGTTATGCTTGTTTGATTACGAATTGTCCTCGT
GGCGGAAAGAGAAGTGATGTAGCATCTTCTTTGAGAACTGTTATTCGAGAAGTATGTTATATTACATTATATTTT
TATCTTTACAGTAAAATTCTTTCGATTTTAATCATCTTGCAAGTAAATTTTATTTTATAAAGACTTTAGCAAATT
CTTATATTTACTCAATTAAACATGTCATTAAAAATTAAATAATTAGAATTTATTTATCTTATATATCTCTTCTAT
TATTTATGTTGCTCTTTTCTATTTATAATGATTTTAATAGTTACATTAATTCAAGAATTGTACTTATTTTTATTT
GCACTGATAAAATTTTGACGATTTGTATTAAAGAAAATTTAGAAGATGATCAATAGAGAATTTATACTTATAGAA
ACAATTATAGAAACAAATTATATTAATCTTAGAAACAAATGTCTGCGTTCCGTTATTTACTGCGAGTATTGAAAA
TCTAATTCACGTATAGTTGTATAGATTTTCAGTATTCACATGAAATTCGAATGAATAACGGAACGGAGCTGTAGC
ATAATTGTAACAAAATTTCAGAAGAACAAAAAATTTCGAGGTTTAATTTTAATTGATTGACAAAAATTGTTCATA
TTATTTAATAATTAATTGGATAATTAGAAGATGTTTATCTATGGAAATATATGAAACAAAAAATTACAAAAAAAT
TTTCTTTATATAATGAAATAAAATATATCTTGATGTTTAAATCTTAAAATTTTTTCTATTTATTTCCTAGTAAAA
TATTCACATAAATATTATTAATGATAAGTCATAAGTCATAAGTAATTTCATAAGTCATAAGTAAATACACAAAAT
GTAATTCGTTCATATTTACCATAACATTCTCCTAGTGTCCTTCATGTGGTCCTAATCATCTGGGTCAATGCTTTG
GGCCTTATATTTGTTGTGGCCCTAGTATCGGTTGCTTCATAGGAACACCAGAAACATTTCGATGCAGAAAGGAAA
GTTTATACACCAGACCCTGCATTGCTGGATACGCAATGTGTCGTGGAAATACAGCAAGATGCGCTTCAAACGGAA
TTTGTTGCTCGCAA
```

Ion transport peptide-like

```
>Cf_ion-transport-peptide-like [organism= Camponotus floridanus] putative
precursor protein (partial)
FTWSLTFLLISSCIGLGADAAAMNAHSLGKRSFFDIQCKGVYDKSIFARLDRICEDCYNLFREPQLHMLCKYV*
>Cf_ion-transport-peptide-like [organism= Camponotus floridanus] (partial)
TTCACGTGGTCCCTGACGTTTCTGCTGATCTCATCTTGCATCGGTCTGGGCGCGGATGCCGCGGCTATGAACGCC
CATTCGCTGGGTAAAAGGTCCTTCTTCGACATCCAGTGCAAGGGCGTGTATGACAAAAGCATCTTCGCCCGTCTG
GACCGCATCTGCGAGGATTGCTACAACTTGTTCCGGGAACCACAACTGCATATGCTTTGCAAGTACGTCTGA


>Ac_ion-transport-peptide-like [organism= Atta cephalotes] putative
precursor protein (partial)
MSVLTWSLTLLLISSCIGLGADAASLSGHPLGKRSFFDIQCKGVYDKSIFARLDRICEDCYNLFREPQLHMLCKQ
DCFSTQYFTSCIQALLLEDEKERLQEMVEYLGRKK*
>Ac_ion-transport-peptide-like [organism= Atta cephalotes] (partial)
ATGTCCGTCCTCACGTGGTCGCTGACGTTACTATTGATCTCGTCCTGCATCGGCCTAGGCGCGGATGCCGCCAGC
CTTAGCGGCCATCCTCTGGGCAAAAGGTCGTTCTTCGACATCCAGTGCAAGGGCGTGTACGACAAGAGCATTTTC
GCCCGTCTGGACCGCATCTGCGAAGACTGCTACAACCTCTTCCGGGAACCGCAATTGCATATGCTTTGCAAGCAA
GACTGCTTCAGCACACAATACTTCACGAGCTGCATCCAAGCGCTGCTGCTTGAGGACGAGAAGGAAAGGTTGCAG
GAGATGGTCGAGTACCTAGGTCGCAAGAAGTAA


>Hs_ion-transport-peptide [organism= Harpegnathos saltator] putative
precursor protein (complete)
MYPSAAYHSSHSTLPSSTSLSASSSSSPASPSSLPLPSRPLSASSSPILLSVLTWSLALLLISSCINLTDARTLN
GHPLSKRSFFDIQCKGVYDKSIFARLDRICEDCYNLFREPQLHMLCKKNCFTTDYFKGCLDVLLLSDEVEKIQMW
IKQLHGADPGV*
>Hs_ion-transport-peptide [organism= Harpegnathos saltator]
ATGTACCCGTCGGCCGCCTACCACTCCTCGCACTCGACGCTACCGTCGTCGACGTCGCTGTCGGCGTCGTCCTCG
TCGTCACCGGCGTCGCCGTCGTCCTTGCCGTTGCCGTCGAGACCGCTCTCGGCGTCGTCGTCTCCGATCCTGCTG
TCTGTCCTCACGTGGTCGCTTGCACTGCTGCTGATCTCGTCCTGCATCAATCTGACGGACGCGCGCACCCTCAAC
GGCCATCCCCTGAGCAAAAGGTCCTTCTTCGATATCCAATGCAAGGGCGTGTACGACAAGAGCATCTTCGCCCGC
CTGGACCGCATCTGCGAAGACTGCTACAACCTCTTCCGGGAGCCGCAGTTGCACATGCTTTGCAAGTAATTGTCA
AGCGATCAAGTCAGCTCCTCCGGGTATCAAATGCGAAAGCCGGTGTTGCGCGCGCGGAGAGAAGTCGCTCGCCTC
CGCGGAGAGGAGAGGAGAGGAGAGGAGAGCCAGCCAGCCAGCCGGAGCTTGAGCGCTGCCGCTTTTCCCGAAACG
CGTCCCGTAATTTGCCGTTTTCCGATCGAGGATCGAGCGCGCCTCTGCTCGGATAATTCTCCCTTCCCGCGACAA
CAACGTCGACGATGACGCAACCGCACGCGGCGTCATCATCGGCGGGGTTAACTTGATATTTTTAACGATGCTCAT
CCTCGAGCGAGCAAAACCGGCCACGCGGGCGAGAACGGTCTTTCCCGCGAAAGACCCGCTAAAAATCGGCCGTTC
AAACGTTCCGCGCTGTCAGGGTCAAGGTGGCGCCAGCGATCGGCAACCTTCGACGTGGACTTGTCCGCTTAGCGT
GTCTATCTTTCATTTCGCTGTGAAACCCGAGCGGGAATTATCGAGCGCCTCGTCTTCTCGGCCGAGACCAAGAGA
AAAGGAATCGAGTTTACGGGATACGCGTGCGGGAGCGCCTCGCCAGCCGGGAAACCCGCGGAGGCGAAGGCGACA
GCGCGCGGAGAGGAAAGTCGCGGGCGACTTCCGCCGCGTGATTTCTACATTCAAAAATGCCGGCGGCGGCGGCTG
ACTCGGCGAACAACGGCCAAGGTGGACAGATACGTACCTTTGTAGGCGCGCGCACACGTGCTCCGGTCTCGGTGC
CACCGTGTACACCGTGCGAATACCCCGAGTCCGCCGCGCGTCGGCACGTGTATCGAAATGGAATGAGATTTCTCT
TCTATTTATAGCTCGCGCGAGCGAATGCCAACGCACATCCGCCGCTACCTGCCGTTTGTGCCGCGCCGCGCTCGG
CCGCTTAACGGCCGTCTCGAAAGAGCTATTGTACCGGCAAACGGGTTCAACGCGAGCAGGGGAAGGGGGATTGAC
GTCCAGCTCGAGAAAGAGAGAGAGAGAGAGAGAGTCTGCGGTTGAGCTTGGAATTTCGAGAAAAATTCATCCGCGCG
TTTTCGTGTGGAAAATTCGCGACGGGGATGGCGGCTGAGTCGATAGTCGAGAGCGGAGCGTTCGAAATTTGTCAC
ACGTTTCCTCGCTGAACGCAGATGTCAGCAGCATTTGTACATTCGTTGTTAATCACTGTACATTCGTCTACTTGG
TTTCTGAGTAATTTTCGCCGTCGGATCTCTCTCGAGTGTCGGCTTGGCGCAGAGGCCAGCTGGTGTTTCTCTCGA
GTCGATGAGAGAGCGCGCTGCGGATATTTCAAAATGACGGGCGGCGTTAATCGGTTAGGTTTTGTCTACTTCCGA
AAAGGAAAGGATATGATCGCGTTGCTTTCGGTGTTACCGCGAAAGTGAGTATGAACGCAGGGTCTCTCTCTCTCT
CTCTCTCTCTCTCTCTCTCTCCCCCTCCCTCTCCCTCTCTCGACTCAACACTCAGCCTGCCCGCGAGAGCAGCAA
AGAAGGAATTCCTCGAATCTCACGGTCGCAATCTAATCACAATACGATACGATTGTAAGGAGTAGATTTGTCGCG
TGAGAGAGGGAGAGAGAGAGAGAGAGAGACGAGAGCGCTCTCGCGCGATCGTTTTCACAGCGCATCCTTCGGGACTT
CCAGGCCACCGGCGGAGCGAATGGCGTTGACTCACGATTCACCGTGTCGTTGTCTTGTTTTCGAAATGTTTGCAG
GAAGAATTGTTTCACGACGGACTACTTTAAAGGATGCTTAGACGTGCTGCTGCTGTCGGACGAGGTGGAGAAAAT
CCAAATGTGGATCAAACAACTCCACGGAGCTGACCCTGGGGTTTAG

//
Gene 1
```

```
Gene 1 2218
  Exon 1 365 phase 0
  Exon 2101 2218 phase 2
//
>HarSal_8001.[1:2218].sp.tr
MYPSAAYHSSHSTLPSSTSLSASSSSSPASPSSLPLPSRPLSASSSPILLSVLTWSLALL
LISSCINLTDARTLNGHPLSKRSFFDIQCKGVYDKSIFARLDRICEDCYNLFREPQLHML
CKKNCFTTDYFKGCLDVLLLSDEVEKIQMWIKQLHGADPGV
//
>HarSal_8001.[1:2218].sp
ATGTACCCGTCGGCCGCCTACCACTCCTCGCACTCGACGCTACCGTCGTCGACGTCGCTG
TCGGCGTCGTCCTCGTCGTCACCGGCGTCGCCGTCGTCCTTGCCGTTGCCGTCGAGACCG
CTCTCGGCGTCGTCGTCTCCGATCCTGCTGTCTGTCCTCACGTGGTCGCTTGCACTGCTG
CTGATCTCGTCCTGCATCAATCTGACGGACGCGCGCACCCTCAACGGCCATCCCCTGAGC
AAAAGGTCCTTCTTCGATATCCAATGCAAGGGCGTGTACGACAAGAGCATCTTCGCCCGC
CTGGACCGCATCTGCGAAGACTGCTACAACCTCTTCCGGGAGCCGCAGTTGCACATGCTT
TGCAAGAAGAATTGTTTCACGACGGACTACTTTAAAGGATGCTTAGACGTGCTGCTGCTG
TCGGACGAGGTGGAGAAAATCCAAATGTGGATCAAACAACTCCACGGAGCTGACCCTGGG
GTTTAG
//
```

CHH-like

```
>Cf_CHH-like [organism= Camponotus floridanus] putative precursor protein
(partial)
RQECFSTEYFTSCMQVLLLEDEKETLQEMARYLGRKK*
>Cf_CHH-like [organism= Camponotus floridanus] (partial)
AGGCAAGAGTGCTTCAGCACAGAATATTTCACGAGCTGCATGCAAGTGCTGCTGCTTGAAGATGAGAAGGAAACG
CTCCAGGAGATGGCCAGGTACCTAGGTCGCAAGAAGTAA
```

Tachykinin-related peptide

```
>Ac_tachykinin-related-peptide [organism= Atta cephalotes] putative
precursor protein (complete)
MLFSSVLFLAVWTSSSFAEESSNDAASAKRAPMGFQGMRGKKDLIPTVAEHNELSKRTLVNFQGMRGKKDYLIPD
FEDSYFLEDYDKRAPMGFQGMRGKKAILEDEYYKRAPMGFQGMRGKKSLEEVLSEIEKRAASLGFYGTRGKKTYI
FEYPQDYEKRLLAMEFQDMHNKIKEEWEKRASMGFQGMRGKKALYDEIEELEKRTLMGFQGMRGKKDGFENYIDY
YIDDPDMDFDKRASMSFQGMRGKKDTDKRAPMGFQGMRGKRSVGQRFEPSTNFGPLNEYQGMGNRRHILASCQVE
KRSPFRYFEMRGKKNPRWELRGMFVGVRGKKWANAPYEDNSPFIRVLDNTERIGMDGDSPTTLGNRLADSQLVSN
ILVAILFIASFIFYAYLNMLVVFIKYQAVYQNVEKEKLNLSLSTQTFKNLLERILNAMYHFQKFLLNITLFYVFT
LHATHRAYLNVFSIFVICVDILYSYCNNCFYSRY*
>Ac_tachykinin-related-peptide [organism= Atta cephalotes]
ATGCTTTTTAGTTCGGTTCTTTTCCTGGCAGTCTGGACTAGTTCGTCATTCGCAGAGGAATCCTCCAATGATGCC
GCGTCTGCCAAACGAGCGCCCATGGGATTTCAAGGTATGCGCGGAAAAAAAGACCTCATTCCTACGGTCGCAGAA
CACAATGAACTTTCCAAAAGAACATTAGTGAATTTTCAGGTAAGCTATTATCTAACTTTTTTTATAAAATTGACA
TAGGAGAAGAAGAAGGAAAGTCTAATTTTATATGTTTAGTTTATTATTTTTTAAATTATTTATAAGATAAAGAGA
TTCCGATAATTACAGGATAAGGTTTCGAGCGCGTCCGAAATTGAGGACAATCTTCTGCACGATGAGTTTGCCAAA
AGAGCACCGAAGGGATTTCAGGGTATGAGGGGGAAAAAGGATTATTTGATACCTGATTTTGAAGACTCCTACTTT
CTTGAGGACTACGACAAAAGAGCGCCAATGGGTTTTCAGGGTATGAGAGGCAAGAAGGCTATATTAGAAGACGAA
TATTATAAACGTGCACCTATGGGATTTCAAGGAATGAGAGGGAAGAAATCTTTGGAGGAGGTAAGCGTGAACATT
TAGTGATTAATTGAATTATTATGCTGCAAGAGCTCAGGACGATAATAGATCAACATTAACAACTTATAAGATTCT
TAAGTATGAAATTATTTACAAAATGGATGTCATAAATTATAGAATTATTCTGCTTTTTCTTCTTTAGTTTTCTCT
ATTCACATGATTAGTTTATCTTTCTTAAATAAACATAATCATTATAAAAAGATTCTTGGTCATTTAAGCATTTTA
AATAGCAATTTAATGTATCTTAAATTAACAGATTTTGATTTGCCTGAAATTTAGTCGTCGTTGTTTATATTTTAT
TTTATTAAAAAATATTAAATTACAGGTGCTGAGTGAAATTGAAAAGAGAGCTGCGTCGTTGGGCTTTTATGGTAC
TAGAGGAAAAAAAACATATATTTTCGAGTATCCACAAGATTACGAAAAGAGACTTTTAGCAATGGAATTCCAAGA
TATGCACAATAAGATAAAAGAAGAATGGGAAAAAAGGGCTTCTATGGGATTTCAGGGGATGAGAGGCAAGAAAGC
ATTGTATGACGAAATAGAAGAACTTGAGAAAAGAACCCTTATGGGTTTTCAGGTAAATTAATTAGTTGCTTAATC
AGTTCTGGAAACATAGATTGTTTTCTATTTAATTGAATATATTTAACTTTCAATATAATTGATAAGAAATATTTA
ATTAAGAAAACGTTAAAAGTTAAAAAAAGAAATGTTTTTAATAATTATTTAATTAATTAATTAATTGTTTTAGGG
CATGAGAGGCAAGAAAGACGGCTTTGAAAATTACATAGATTATTACATAGATGATCCCGACATGGATTTTGACAA
GAGGGCATCAATGAGTTTTCAAGGGATGAGAGGCAAGAAAGACACCGATAAGAGAGCACCGATGGGTTTTCAAGG
TATGCGGGGCAAGAGGAGTGTAGGACAAAGATTTGAGCCCAGCACGAATTTTGGACCATTAAACGAATATCAAGG
TAAGGTCATATTGCTTACATTCTATATTTATGATAATTGAAAAAGAGAGAAACATTTGAATATCATTATAATTAA
CATTTGTTAGTAATATTATTTTTCATAAAACATTCATAACTCTACAATTATTAATGACTTACAAAATAATTTATT
```

```
TGTAAGAAAGATTTTATTAATAAGTTATAAAATAATTGAGTTTTATACTTAATTTATTAATTAATTACTTCGAAA
CTTATGTGATCTCTAATTTCATAAATCTTGCTTTAATCATTATAATATGTAAATATGTTAAAACTTGTATTATCT
TTAATATAAATTTTAATATGAAAAATGGTTCATGCGGAAAGTTAAAAATTTCTCTTTTTATATGAGAAGTTTTGA
GAAGCTGGAGACACGATTACTTGCGATATTAAAACGAAATAAAAATTATTTGCATACGGGAGCAGTTTTTATCAG
TGTATGTTTGATTAAATGTGTTATAACTCGATGAAAAATATTTTCTGTCGTTTTTATTGCGCACTCTAGAATGTC
TACTCGTACTTTTTGAATGCCCCTTGTAATATTACTCGACGATATAATCGATCGAGTCTAAGTGGAACGTAACGA
AGGCTTCCCAAATATTTGTCTATTATTCATGCACCTCCAAACAAACTCTGTAGTGAGAAGGTAGACAATTTTTTT
TATTTTCTTTTTTTTTACCGTTCTTATATATGCCGTGCGATTTTGAGGTCGAAATGGTCACATTGGCCCACGTTG
TGTTTCCCCCGAGCAGGAATGGGCAATAGAAGACACATTCTAGCCTCGTGCCAAGTCGAAAAACGATCGCCTTTC
CGATATTTTGAGATGCGCGGTAAGAAAAATCCACGATGGGAGTTACGGGGGATGTTTGTGGGGGTGAGAGGCAAA
AAATGGGCGAATGCCCCGTACGAGGACAACAGCCCATTCATAAGGGTGCTTGATAACACCGAGAGGATCGGGATG
GATGGGGATTCGCCGACAACATTAGGTAATCGATTGGCTGACAGTCAATTGGTATCAAACATTCTTGTCGCAATT
CTCTTTATCGCTTCGTTTATCTTTTATGCGTATCTAAATATGCTTGTAGTATTTATTAAGTATCAAGCAGTTTAC
CAGAATGTAGAGAAAGAAAAATTAAATTTATCTCTTTCCACACAAACATTCAAAAATCTTCTTGAGCGTATTTTA
AATGCAATGTATCATTTTCAAAAATTTTTGCTAAATATTACACTCTTTTATGTATTTACTTTGCATGCGACACAT
CGAGCATATTTAAATGTATTCAGTATATTTGTAATATGTGTCGATATACTTTATTCTTACTGTAATAATTGTTTT
TATTCACGCTATTGA
//
Gene 1
Gene 10749 13226
  Exon 10749 10937 phase 0
  Exon 11145 11333 phase 0
  Exon 11674 11925 phase 0
  Exon 12097 12322 phase 0
  Exon 13090 13226 phase 1
//
FT          CDS    join(10749..10937,11145..11333,11674..11925,12097..12322,
FT                   13090..13226)
FT                   /note="Match to TACHY_APIME_Tachykinins"
//
>Atta_cephalotes_contig00331.[10749:13226].sp.tr
MLFSSVLFLAVWTSSSFAEESSNDAASAKRAPMGFQGMRGKKDLIPTVAEHNELSKRTLV
NFQGMRGKKDYLIPDFEDSYFLEDYDKRAPMGFQGMRGKKAILEDEYYKRAPMGFQGMRG
KKSLEEVLSEIEKRAASLGFYGTRGKKTYIFEYPQDYEKRLLAMEFQDMHNKIKEEWEKR
ASMGFQGMRGKKALYDEIEELEKRTLMGFQGMRGKKDGFENYIDYYIDDPDMDFDKRASM
SFQGMRGKKDTDKRAPMGFQGMRGKRSVGQRFEPSTNFGPLNEYQGMGNRRHILASCQVE
KRSPFRYFEMRGKKNPRWELRGMFVGVRGKK
//
>Atta_cephalotes_contig00331.[10749:13226].sp
ATGCTTTTTAGTTCGGTTCTTTTCCTGGCAGTCTGGACTAGTTCGTCATTCGCAGAGGAA
TCCTCCAATGATGCCGCGTCTGCCAAACGAGCGCCCATGGGATTTCAAGGTATGCGCGGA
AAAAAAGACCTCATTCCTACGGTCGCAGAACACAATGAACTTTCCAAAAGAACATTAGTG
AATTTTCAGGGTATGAGGGGGAAAAAGGATTATTTGATACCTGATTTTGAAGACTCCTAC
TTTCTTGAGGACTACGACAAAAGAGCGCCAATGGGTTTTCAGGGTATGAGAGGCAAGAAG
GCTATATTAGAAGACGAATATTATAAACGTGCACCTATGGGATTTCAAGGAATGAGAGGG
AAGAAATCTTTGGAGGAGGTGCTGAGTGAAATTGAAAAGAGAGCTGCGTCGTTGGGCTTT
TATGGTACTAGAGGAAAAAAAACATATATTTTCGAGTATCCACAAGATTACGAAAAGAGA
CTTTTAGCAATGGAATTCCAAGATATGCACAATAAGATAAAAGAAGAATGGGAAAAAAGG
GCTTCTATGGGATTTCAGGGGATGAGAGGCAAGAAAGCATTGTATGACGAAATAGAAGAA
CTTGAGAAAAGAACCCTTATGGGTTTTCAGGGCATGAGAGGCAAGAAAGACGGCTTTGAA
AATTACATAGATTATTACATAGATGATCCCGACATGGATTTTGACAAGAGGGCATCAATG
AGTTTTCAAGGGATGAGAGGCAAGAAAGACACCGATAAGAGAGCACCGATGGGTTTTCAA
GGTATGCGGGGCAAGAGGAGTGTAGGACAAAGATTTGAGCCCAGCACGAATTTTGGACCA
TTAAACGAATATCAAGGAATGGGCAATAGAAGACACATTCTAGCCTCGTGCCAAGTCGAA
AAACGATCGCCTTTCCGATATTTTGAGATGCGCGGTAAGAAAAATCCACGATGGGAGTTA
CGGGGGATGTTTGTGGGGGTGAGAGGCAAAAAA
//
```
