# Supplementary material for: Discovery of Defense- and Neuropeptides in Social Ants by Genome-Mining
Source: PLoS One. 2012 Mar 20;7(3):e32559. doi: 10.1371/journal.pone.0032559 (PMC3308954; doi:10.1371/journal.pone.0032559)
Supplement: File S3 — GeneWise raw data for prediction of gene structure. (PDF) [file pone.0032559.s010.pdf]

GeneWise data of selected ant peptide genes and control organisms

Experimental algorithm (for all queries):

**Query protein:**	**as indicated**
Comp Matrix:	blosum62.bla
Gap open:	12
Gap extension:	2
Start/End	local
**Target Sequence:**	**species and contig as indicated (see Methods Section)**
Strand:	forward
Start/End (protein)	local
Gene Paras:	human.gf
Codon Table:	codon.table
Subs error:	1e-05
Indel error:	1e-05
Model splice	model
Model codon bias	flat
Model intron bias	tied
Null model	syn
Algorithm	623

1. Defensins:

Query protein: DEF_FORAQ_Defensin
Target Sequence CamFlo, contig 2890

```
genewise output
Score 130.35 bits over entire alignment
Scores as bits over a synchronous coding model

DEF_FORAQ_Defen    1 MKNYVFALLVVTAVAIALPNE                         KN
                     MK YVF LLVVTA+A+A P E                         +N
                     MKIYVFTLLVVTAIAVAFPTE          D:E[gag]       EN
CamFlo_1.0_4.co  652 aaatgtactggagagggtcagGGTATTTA  Intron 1   TAGAGga
                     tatattcttttcctctctcca <1-----[716  : 1126]-1>  aa
                     ggccacttggggacctactta                         at


DEF_FORAQ_Defen   25 APMRV-HLLPQKEDESLKLEVTPVKEHHRTRRFTCDLLSGAGVDHSACA
                       +     L  K+D+SL    TP+KEH+RTRR TCDLLSG GV+HSACA
                     VTVESPDFLILKKDKSL--QETPIKEHNRTRRATCDLLSGFGVNHSACA
CamFlo_1.0_4.co 1135 gaggtcgttacaagatt  cgacaagcacacagatgcttgtggacagtg
                     tctaccattttaaaact  aacctaaaagcggccgattcgtgtaagcgc
                     agagtgctgagaatatg  agatcaatttctatctttatccttttctca


DEF_FORAQ_Defen   73 AHCILRGKTGGRCNSDRVCVCRA
                     AHCILRGKTGGRCNS+ VCVCRA
                     AHCILRGKTGGRCNSNAVCVCRA
CamFlo_1.0_4.co 1276 gctacagaaggataaaggtgtcg
                     cagttggacggggagactgtggc
                     tcctagaacgaatctcttcgtcg
//
Manually refined intron-exon borders
Gene 1
Gene 652 1344
  Exon 652 715 phase 0
  Exon 1127 1344 phase 1
//
FT          CDS    join(652..715,1127..1344)
FT                   /note="Match to DEF_FORAQ_Defensin"
//
>CamFlo_1.0_4.contig2890,.[652:1344].sp.tr
MKIYVFTLLVVTAIAVAFPTEELELEENVTVESPDFLILKKDKSLQETPIKEHNRTRRATC
DLLSGFGVNHSACAAHCILRGKTGGRCNSNAVCVCRA
//
>CamFlo_1.0_4.contig2890,.[652:1344].sp
ATGAAGATCTACGTATTCACTCTTTTGGTGGTGACGGCAATCGCCGTTGCATTCCCTACT
GAAGAGCTAGAGCTAGAGGAAAATGTAACGGTAGAGTCTCCGGACTTTTTGATACTGAAA
AAAGATAAATCTTTGCAAGAGACACCTATCAAAGAACATAATCGTACCCGTAGAGCTACC
TGTGATCTTTTATCTGGCTTCGGTGTTAATCATAGCGCTTGCGCAGCTCACTGCATTCTA
AGGGGAAAAACCGGGGGAAGATGTAACAGTAACGCTGTTTGCGTGTGTCGCGCG//
```

Query protein: DEF2_APIME_Defensin-2
Target Sequence HarSal, contig 7503

```
genewise output
Score 114.89 bits over entire alignment
Scores as bits over a synchronous coding model

DEF2_APIME_Defe    1 MKFFVLFAILIAIVHASCASVPKVVYDGPIYEL
                     MK++ +FA+   + +AS +++P  VYDGPIYEL
                     MKLLAIFALFCVLAYASASALP-AVYDGPIYEL
HarSal_7503     1252 aaccgatgcttgtgtgtgagtc ggtggcatgc
                     tattcttcttgttcacccgctc ctaagctaat
                     gggctccccccgggccggcggt ccctagttgg


DEF2_APIME_Defe   34                          QIEEENIEPDTELMDSNEPLLPL
                                               IEE     D    D  E + P+
                               R:T[acc]       PIEE--AASDNTPRDMAEEV-PI
HarSal_7503     1348 AGTAAGCA  Intron 1   CAGCCcagg  ggtgaaccgagggg ca
                      <1-----[1349 : 6050]-1>  ctaa  cccaaccgatcaat ct
                                              ctga  ggcccgagcgagag cc


DEF2_APIME_Defe   58 RHRRVTCDVLSWQSKWLSINHSACAIRCLAQRRKGGSCRNGVCICR
                     R RRVTCD+LSW SKW S+N+SACA +CL QRR+GGSC  GVC+CR
                     RQRRVTCDLLSWTSKWFSFNNSACAAKCLVQRRRGGSCSGGVCVCR
HarSal_7503     6113 ccccgatgccttaaattataaagtggattgccccggttagggtgta
                     gaggtcgattcgcgagtgtaagcgccagttagggggcggggtgtgg
                     agtgccccccctggcggccccctccgagcagattgcttctctccccg


//
Gene 1
Gene 1252 6250
  Exon 1252 1348 phase 0
  Exon 6051 6250 phase 1
//
FT          CDS    join(1252..1348,6051..6250)
FT                   /note="Match to DEF2_APIME_Defensin-2"
//
>HarSal_7503.[1252:6250].sp.tr
MKLLAIFALFCVLAYASASALPAVYDGPIYELTPIEEAASDNTPRDMAEEVPIRQRRVTC
DLLSWTSKWFSFNNSACAAKCLVQRRRGGSCSGGVCVCR
//
>HarSal_7503.[1252:6250].sp
ATGAAGCTGCTCGCTATCTTCGCCCTCTTCTGCGTGTTGGCGTACGCCTCGGCGAGCGCG
TTGCCTGCCGTCTACGATGGACCGATTTATGAGCTGACCCCCATTGAGGAAGCGGCGTCC
GACAACACGCCACGGGACATGGCAGAGGAAGTGCCCATCCGACAGCGTCGGGTCACCTGC
GACCTCCTCTCTTGGACGAGCAAGTGGTTCAGCTTCAACAACAGTGCCTGCGCGGCAAAG
TGCTTAGTGCAACGTCGTCGGGGCGGTTCTTGCAGTGGCGGTGTCTGCGTCTGCAGG
//
```

Query protein: DEF2_APIME_Defensin-2
Target Sequence Atta cephalotes, contig 21145

```
genewise output
Score 124.58 bits over entire alignment
Scores as bits over a synchronous coding model

DEF2_APIME_Defe    6 LFAILIAIVHASCASVPKV--VYDGPIYEL
                     LFAIL+ +V  +C SV  +  VYDGP YEL
                     LFAILVIFVVLACTSVSTLPAVYDGPTYEL
Atta_cephalotes  345 ctgacgatggtgtatgaaccggtggcatgc
                     ttcttttttttcgcctgctcctaagccaat
                     gctcctcccagttcaccaagtctcatttag


DEF2_APIME_Defe   34                           QIEEENIEPDTELMDSNEPLLPL
                                                IE+    P  + M S+  L P+
                               R:T[act]        TIED----PVNDEMPSD--LSPI
Atta_cephalotes  435 AGTAAGCA  Intron 1   CAGCTaagg    cgaggactg  ctca
                      <1-----[436  : 2848]-1>  ctaa    ctaaatcca  tcct
                                               ccgt    agctggggt  ggcc


DEF2_APIME_Defe   58 RHRRVTCDVLSWQSKWLSINHSACAIRCLAQRRKGGSCRNGVCICR
                     R+RRVTCD+LSWQSKWLSINHSACA RCL+QRRKGG CR+G+C+CR
                     RNRRVTCDLLSWQSKWLSINHSACAARCLSQRRKGGRCRDGICVCR
Atta_cephalotes 2902 caccgatgccttctattaaacagtggatctcccaggctcggatgta
                     gaggtcgattcgacagtgtaagcgccggtcaggagggggagtgtgg
                     acgcgccctgcggcggatctctccatacggaacatccccctcccca


//
Gene 1
Gene 345 3039
  Exon 345 435 phase 0
  Exon 2849 3039 phase 1
//
FT          CDS    join(345..435,2849..3039)
FT                   /note="Match to DEF2_APIME_Defensin-2"
//
>Atta_cephalotes_contig21145,.[345:3039].sp.tr
LFAILVIFVVLACTSVSTLPAVYDGPTYELTTIEDPVNDEMPSDLSPIRNRRVTCDLLSW
QSKWLSINHSACAARCLSQRRKGGRCRDGICVCR
//
>Atta_cephalotes_contig21145,.[345:3039].sp
CTGTTCGCTATCCTCGTTATCTTCGTCGTATTGGCTTGTACCTCAGTCAGCACACTACCG
GCTGTCTATGACGGACCTACTTATGAACTGACTACCATCGAGGATCCAGTGAACGATGAG
ATGCCGTCGGATCTGTCGCCCATCCGAAACCGGCGCGTGACCTGCGACCTTCTGTCCTGG
CAGTCCAAGTGGTTAAGTATCAATCACAGTGCCTGCGCAGCTAGATGCCTGTCGCAACGA
CGCAAAGGTGGCCGCTGCCGCGACGGTATCTGCGTCTGCAGA
//
```

Query protein: DEF2_APIME_Defensin-2
Target Sequence CamFlo, contig 2676

```
genewise output
Score 121.81 bits over entire alignment
Scores as bits over a synchronous coding model

DEF2_APIME_Defe    1 MKFFVLFAILIAIVHASCASVPKVVYDGPIYEL
                     MK++V+FA    + + S A+    VYDGP YEL
                     MKLLVIFATFAVLAYVS-ANTLSAVYDGPTYEL
CamFlo_2676    25203 aaccgatgatggcgtgt gaactggtggcatgt
                     tatttttcctcttcatc cactcctaagccaat
                     gggctccctttaatctg acaagtttcattcag


DEF2_APIME_Defe   34                           QIEEENIEPDTELMDSNEPLLPL
                                                I+E    P  + M SN  L P+
                               R:T[acc]        TIDE----PQYDEMASN--LSPI
CamFlo_2676    25299 AGTAAGTA  Intron 1   CAGCCaagg    cctggagta  ctca
                      <1-----[25300:26342]-1>  ctaa    caaaatcca  tcct
                                               cccg    gaccggagt  ggcc


DEF2_APIME_Defe   58 RHRRVTCDVLSWQSKWLSINHSACAIRCLAQRRKGGSCRNGVCICR
                     RHRRVTCD+LSWQS+WL+INHSACA +CL QRR+GG CR+G+C+CR
                     RHRRVTCDLLSWQSQWLTINHSACAAKCLVQRRRGGRCRDGICVCR
CamFlo_2676    26396 ccccgatgctttctcttaaacagtggatcgccccggctcggatgta
                     gaggtcgattcgacagtctaagcgccagttagggggggagtgtgg
                     acgcaccctaggacaggcacctgcgtacgggttgcccccccccccg


//
Gene 1
Gene 25203 26533
  Exon 25203 25299 phase 0
  Exon 26343 26533 phase 1
//
FT          CDS    join(25203..25299,26343..26533)
FT                   /note="Match to DEF2_APIME_Defensin-2"
//
>CamFlo_2676.[25203:26533].sp.tr
MKLLVIFATFAVLAYVSANTLSAVYDGPTYELTTIDEPQYDEMASNLSPIRHRRVTCDLL
SWQSQWLTINHSACAAKCLVQRRRGGRCRDGICVCR
//
>CamFlo_2676.[25203:26533].sp
ATGAAGCTGCTCGTTATCTTCGCCACTTTTGCTGTACTAGCTTACGTTTCGGCAAACACA
CTATCGGCTGTTTATGACGGACCTACTTACGAATTGACCACCATCGACGAGCCGCAATAC
GACGAGATGGCATCGAATCTGTCGCCCATCCGACACCGGCGCGTAACCTGCGACCTTTTA
TCGTGGCAATCCCAATGGTTGACCATAAACCACAGTGCGTGCGCGGCTAAATGCCTGGTG
CAGCGTCGTCGGGGCGGCCGCTGCCGCGACGGCATCTGCGTCTGCAGG
//
```

```
Query protein:       DEF1_APIME_Defensin-1 (NP_001011616.2)
Target Sequence      NC_007075.3

NP_001011616.2     1 MKIYFIVGLLFMAMVAIMAAPV                        D
                     MKIYFIVGLLFMAMVAIMAAPV                        D
                     MKIYFIVGLLFMAMVAIMAAPV          E:E[gag]      D
8886984-8888512   71 aaattaggcctagaggaaggcgGGTGGGTA  Intron 1   TAGAGg
                     tatatttgttttcttcttccct <1-----[138  :  708]-1>  a
                     gactttcctccgtgttcgtatt                        t


NP_001011616.2    25 EFEPLEHFENEERADRHRRVTCDLLSFKGQVNDSACAANCLSLGKAGGH
                     EFEPLEHFENEERADRHRRVTCDLLSFKGQVNDSACAANCLSLGKAGGH
                     EFEPLEHFENEERADRHRRVTCDLLSFKGQVNDSACAANCLSLGKAGGH
8886984-8888512  714 gtgccgctgaggcggacaagatgccttagcgagagtggatcatgagggc
                     atactaataaaagcagaggtcgattctagataagcgccagtgtgacgga
                     acgatgttgtaatccataaattctcacaaattcttcttctctgtatatt


NP_001011616.2    74 CEKGVCICR                          TSFKDLWDKRFG
                     CEKGVCICR                          TSFKDLWDKRFG
                     CEKGVCICR           K:K[aaa]       TSFKDLWDKRFG
8886984-8888512  861 tgaggtatcAAGTATGTA  Intron 2   CAGAaatagctgactg
                     gaagtgtgg  <2-----[890  : 1167]-2> cgtaatgaagtg
                     cgaatttta                          ctcatcgcatct


//
Gene 1
Gene 71 1204
  Exon 71 137 phase 0
  Exon 709 889 phase 1
  Exon 1168 1204 phase 2
//
>8886984-8888512.[71:1204].sp.tr
MKIYFIVGLLFMAMVAIMAAPVEDEFEPLEHFENEERADRHRRVTCDLLSFKGQVNDSAC
AANCLSLGKAGGHCEKGVCICRKTSFKDLWDKRFG
//
>8886984-8888512.[71:1204].sp
ATGAAAATCTATTTTATTGTCGGCCTTCTCTTCATGGCTATGGTTGCTATCATGGCTGCA
CCTGTTGAGGATGAATTCGAGCCACTTGAGCATTTTGAGAATGAAGAACGTGCCGACAGA
CATAGAAGAGTAACTTGTGACCTTCTCTCATTCAAAGGACAAGTTAATGACAGTGCTTGC
GCTGCTAACTGTCTCAGTTTGGGTAAAGCTGGAGGTCATTGCGAGAAAGGAGTTTGTATT
TGTCGAAAAACCAGTTTCAAAGATCTCTGGGACAAACGTTTCGGT
//
```

Query protein: DEF2_APIME_Defensin-2 (NP_001011638.1)
Target Sequence NC_007085.3

```
NP_001011638.1     1 MKFFVLFAILIAIVHASCASVPKVVYDGPIYEL
                     MKFFVLFAIL+AIVHASCASVPKVVYDGPIYEL
                     MKFFVLFAILVAIVHASCASVPKVVYDGPIYEL
4369928-4370847   17 aattgctgacggagcgttgagcaggtggcatgt
                     tatttttctttcttaccgcgtcattaagctaat
                     ggctattgtcctctcatcatttattctagtcgg


NP_001011638.1    34                           QIEEENIEPDTELMDSNEPLLPL
                                               QIEEENIEPDTELMDSNEPLLPL
                               R:R[agg]        QIEEENIEPDTELMDSNEPLLPL
4369928-4370847  116 AGTGAGTA  Intron 1   TAGGGcagggaagcgagtagtagccccc
                      <1-----[117  :  452]-1>  ataaaatacacattacaacttct
                                               atggataaataaggtccaggaaa


NP_001011638.1    58 RHRRVTCDVLSWQSKWLSINHSACAIRCLAQRRKGGSCRNGVCICRK
                     RHRRVTCDVLSWQSKWLSINHSACAIRCLAQRRKGGSCRNGVCICRK
                     RHRRVTCDVLSWQSKWLSINHSACAIRCLAQRRKGGSCRNGVCICRK
4369928-4370847  524 cccagatggtttctatcaaactgtgaattgcccaggataaggtatca
                     gaggtcgattcgacagtgtaaccgctggtcaggaggggagtgtgga
                     atagagcctaagaaaggctttatctcatataatacttcatcgtctag


//
Gene 1
Gene 17 664
  Exon 17 116 phase 0
  Exon 453 664 phase 1
//
>4369928-4370847.[17:664].sp.tr
MKFFVLFAILVAIVHASCASVPKVVYDGPIYELRQIEEENIEPDTELMDSNEPLLPLRHR
RVTCDVLSWQSKWLSINHSACAIRCLAQRRKGGSCRNGVCICRK
//
>4369928-4370847.[17:664].sp
ATGAAGTTCTTTGTACTTTTTGCGATTCTCGTCGCTATCGTTCACGCATCTTGCGCAAGT
GTTCCTAAAGTTGTTTACGATGGACCGATTTACGAGTTGAGGCAAATTGAGGAGGAAAAT
ATAGAACCAGATACAGAATTGATGGATTCCAACGAACCGCTGCTACCACTACGACATCGA
AGGGTAACGTGCGACGTTTTATCATGGCAATCAAAATGGCTGAGCATTAATCATTCAGCT
TGCGCTATCAGATGTTTAGCTCAACGACGTAAAGGCGGTAGTTGCAGAAATGGCGTGTGT
ATCTGTCGAAAG
//
```

Query protein: DEF_DROME_Defensin (NP_523672.1)
Target Sequence NT_033778.3

```
NP_523672.1        1 MKFFVLVAIAFALLACVAQAQPVSDVDPIPEDHVLVHEDAHQEVLQHSR
                     MKFFVLVAIAFALLACVAQAQPVSDVDPIPEDHVLVHEDAHQEVLQHSR
                     MKFFVLVAIAFALLACVAQAQPVSDVDPIPEDHVLVHEDAHQEVLQHSR
c5942081-594168   10 aattgcggagtgccgtggcgccgtgggcacggcgcgcgggccggcccac
                     tatttttctctcttcgtcacactcatactcaaatttaaacaaattaagg
                     ggcctcgtctttgttcgggtgatctgtatagttcggtgtccgggggtcc


NP_523672.1       50 QKRATCDLLSKWNWNHTACAGHCIAKGFKGGYCNDKAVCVCRN
                     QKRATCDLLSKWNWNHTACAGHCIAKGFKGGYCNDKAVCVCRN
                     QKRATCDLLSKWNWNHTACAGHCIAKGFKGGYCNDKAVCVCRN
c5942081-594168  157 cacgatgcctatatacagtggctagagtaggttagaggtgtca
                     aagccgattcagagaaccgcgagtcagtaggagaaactgtgga
                     ggacaccaccggcgccccccccctcggcaccccccgccctcct


//
Gene 1
Gene 10 285
  Exon 10 285 phase 0
//
>c5942081-5941683.[10:285].sp.tr
MKFFVLVAIAFALLACVAQAQPVSDVDPIPEDHVLVHEDAHQEVLQHSRQKRATCDLLSK
WNWNHTACAGHCIAKGFKGGYCNDKAVCVCRN
//
>c5942081-5941683.[10:285].sp
ATGAAGTTCTTCGTTCTCGTGGCTATCGCTTTTGCTCTGCTTGCTTGCGTGGCGCAGGCT
CAGCCAGTTTCCGATGTGGATCCAATTCCAGAGGATCATGTCCTGGTGCATGAGGATGCC
CACCAGGAGGTGCTGCAGCATAGCCGCCAGAAGCGAGCCACATGCGACCTACTCTCCAAG
TGGAACTGGAACCACACCGCCTGCGCCGGCCACTGCATTGCCAAGGGGTTCAAAGGCGGC
TACTGCAACGACAAGGCCGTCTGCGTTTGCCGCAAT
//
```

2. AVP:

Query protein: A3RE83_TRICA_Arginine-vasopressin-like-peptide
Target Sequence HarSal, contig 1385

```
genewise output
Score 119.03 bits over entire alignment
Scores as bits over a synchronous coding model

A3RE83_TRICA_Ar   21 CLITNCPRGGKRSKFAISENAV-KP                       C
                     CLITNCPRGGKR  +  S   V                          C
                     CLITNCPRGGKRGDIIPSLGTVTRE                       C
HarSal_1385    31248 ttaaatccggaaggaacttgagacgGTGAGTA  Intron 1   CAGt
                     gttcagcgggaggattcctgctcga<0-----[31323:31595]-0>g
                     tatgtttctaaatctattgatctaa                       c


A3RE83_TRICA_Ar   46 VSCGPGQSGQCFGPSICCGP-FGCLVGTPETLRCQREGFFHEREPCIAG
                       CGP   GQCFGP ICCGP  GC +GTPET RC++E  +    PCIAG
                     PPCGPNHLGQCFGPHICCGPTIGCFIGTPETYRCRKESPY--ARPCIAG
HarSal_1385    31599 cctgcaccgcttgccattgcaagttagacgatataagact  gactagg
                     ccggcaatgagtgcatgggcctggttgccacagggaagca  cgcgtcg
                     tattcttgtactgtctccctctacctaaaaacacagatat  tatccac


A3RE83_TRICA_Ar   94 SAPCRKNTGRCAFDGICCSQ                          SCH
                      A CR NT RCA +GICCSQ                          SCH
                     YAMCRGNTARCATNGICCSQ          D:D[gac]        SCH
HarSal_1385    31740 tgatcgaagatgaagatttcGGTAAATA  Intron 2   TAGACttc
                     actgggaccggccagtggca <1-----[31801:32136]-1>  cga
                     tagctacatattatattcaa                          tcc


A3RE83_TRICA_Ar  118 ADKSCASDD
                      D SC   D
                     MDTSCRISD
HarSal_1385    32148 agattaatg
                     taccggtca
                     gtagcattt


//
Gene 1
Gene 31248 32174
  Exon 31248 31322 phase 0
  Exon 31596 31800 phase 0
  Exon 32137 32174 phase 1
//
FT          CDS    join(31248..31322,31596..31800,32137..32174)
FT                   /note="Match to A3RE83_TRICA_Arginine-vasopressin-like-peptide"
//
>HarSal_1385.[31248:32174].sp.tr
CLITNCPRGGKRGDIIPSLGTVTRECPPCGPNHLGQCFGPHICCGPTIGCFIGTPETYRC
RKESPYARPCIAGYAMCRGNTARCATNGICCSQDSCHMDTSCRISD
//
>HarSal_1385.[31248:32174].sp
TGTTTAATTACGAATTGTCCTCGCGGTGGAAAAAGAGGTGACATTATACCTTCTTTGGGA
ACTGTCACTCGAGAATGCCCTCCATGTGGTCCCAATCATCTGGGTCAATGCTTTGGGCCT
CACATTTGCTGCGGCCCTACCATTGGATGCTTCATTGGAACACCAGAAACATACAGATGC
AGAAAGGAAAGTCCATATGCTAGACCTTGCATCGCAGGCTATGCAATGTGCCGTGGAAAC
ACAGCTAGATGTGCTACAAATGGAATTTGTTGCTCACAAGACTCTTGCCACATGGATACA
TCGTGCAGAATTTCTGAT
//
```

Query protein: A3RE83_TRICA_Arginine-vasopressin-like-peptide
Target Sequence NC_007423.2

```
ABX52000.1          1 MSTIITSIILLVLSESLVSGCLITNCPRGGKRSKFAISENAVKP
                        MSTIITSIILLVLSESLVSGCLITNCPRGGKRSKFAISENAVKP
                        MSTIITSIILLVLSESLVSGCLITNCPRGGKRSKFAISENAVKP
c14920575-14919    19 ataaaataatcgtagttgtgtcaaatccggacaatgaagaggac
                        tccttcctttttgacttcggttcagcgggaggatctgaactac
                        gcacctgctactgcatataatctccttcccagcgtcatacccag


ABX52000.1         45                         CVSCGPGQSGQCFGPSICCGPFGCLV
                                              CVSCGPGQSGQCFGPSICCGPFGCLV
                                              CVSCGPGQSGQCFGPSICCGPFGCLV
c14920575-14919   151 GTAAGAA  Intron 1   CAGtgatgcgcagcttgcaattgctgtcg
                      <0-----[151  :  194]-0>gtgggcgaggagtgcgtgggctggtt
                                              tgtctccacgatcccctccatcgttg


ABX52000.1         71 GTPETLRCQREGFFHEREPCIAGSAPCRKNTGRCAFDGICCSQ
                      GTPETLRCQREGFFHEREPCIAGSAPCRKNTGRCAFDGICCSQ
                      GTPETLRCQREGFFHEREPCIAGSAPCRKNTGRCAFDGICCSQ
c14920575-14919   273 gacgacctccggttcgcgctaggagctaaaagctgtggattac
                      gccactggagagttaagacgtcggccggaacgggctagtggga
                      accgggtcatgctccacgcctcccccgacaggctccgttcta


ABX52000.1        114                            SCHADKSCASDDKSPIDLYTLIN
                                                 SCHADKSCASDDKSPIDLYTLIN
                                D:D[gac]         SCHADKSCASDDKSPIDLYTLIN
c14920575-14919   402 GGTAATTT  Intron 2   CAGACttcggaatgaggaacagttacaa
                       <1-----[403  :  451]-1>  cgacaaggcgaaagctatactta
                                                 tctgtactctctatgatgctctt


ABX52000.1        138 YQAELAGDK
                      YQAELAGDK
                      YQAELAGDK
c14920575-14919   523 tcggtggga
                      aacatcgaa
                      catggttta


//
Gene 1
Gene 19 549
  Exon 19 150 phase 0
  Exon 195 402 phase 0
  Exon 452 549 phase 1
//
>c14920575-14919920.[19:549].sp.tr
MSTIITSIILLVLSESLVSGCLITNCPRGGKRSKFAISENAVKPCVSCGPGQSGQCFGPS
ICCGPFGCLVGTPETLRCQREGFFHEREPCIAGSAPCRKNTGRCAFDGICCSQDSCHADK
SCASDDKSPIDLYTLINYQAELAGDK
//
>c14920575-14919920.[19:549].sp
ATGTCCACAATCATCACTTCGATCATTTTACTCGTTTTGAGCGAATCTTTAGTTTCAGGA
TGTCTCATTACCAACTGTCCTCGCGGCGGCAAACGGAGCAAGTTTGCCATAAGTGAAAAC
GCCGTCAAACCGTGTGTGAGTTGCGGTCCCGGCCAAAGCGGGCAATGTTTCGGCCCCAGC
ATTTGCTGCGGACCTTTCGGGTGTCTTGTGGGAACCCCCGAGACGCTGCGTTGCCAACGT
GAGGGCTTTTTCCACGAACGCGAGCCCTGCATTGCCGGCAGCGCCCCCTGCAGGAAAAAC
ACAGGGCGGTGCGCTTTCGACGGGATTTGTTGCAGTCAAGACTCTTGCCATGCGGATAAA
AGCTGTGCCAGTGACGATAAAAGTCCGATAGATTTGTACACTCTCATTAATTACCAAGCT
GAGTTGGCTGGTGATAAA
//
```

Query protein: NP_000906.1| oxytocin-neurophysin 1 preproprotein [Homo sapiens]
Target Sequence NC_000020.10

```
NP_000906.1        1 MAGPSLACCLLGLLALTSACYIQNCPLGGKRAAPDLDVRK
                     MAGPSLACCLLGLLALTSACYIQNCPLGGKRAAPDLDVRK
                     MAGPSLACCLLGLLALTSACYIQNCPLGGKRAAPDLDVRK
3052266-3053162   37 aggcacgttccgccgcatgttacatccggaaggcgcggca
                     tcgcgtcggttgttctcccgataagctggagcccatatga
                     gccccctctgcccgggcccccgcccgacggcggcccgcg


NP_000906.1       41                        CLPCGPGGKGRCFGPNICCAEELGCF
                                            CLPCGPGGKGRCFGPNICCAEELGCF
                                            CLPCGPGGKGRCFGPNICCAEELGCF
3052266-3053162  157 GTGAGTC  Intron 1   CAGtcctgcggagcttgcaattgggcgtt
                     <0-----[157  :  457]-0>gtcggcggagggtgcatggcaatggt
                                            cccccgcaccccgctcccgaggccc


NP_000906.1       67 VGTAEALRCQEENYLPSPCQSGQKACGSGGRCAVLGLCCSP
                     VGTAEALRCQEENYLPSPCQSGQKACGSGGRCAVLGLCCSP
                     VGTAEALRCQEENYLPSPCQSGQKACGSGGRCAVLGLCCSP
3052266-3053162  536 ggagggcctcggatcctctctgcagtgaggctggtgcttac
                     tgccactggaaaaatcccgacgaacgggggggcttgtgggc
                     gcccaggccgggccgggccgccgggcgcgcccgcgcccccg


NP_000906.1      108                           GCHADPACDAEATFSQR
                                               GCHADPACDAEATFSQR
                               D:D[gac]        GCHADPACDAEATFSQR
3052266-3053162  659 GGTGAGCG  Intron 2   CAGACgtcggcgtggggattcc
                      <1-----[660  :  743]-1>  ggacaccgacacctcag
                                               ccccctcccgaccccgc


//
Gene 1
Gene 37 796
  Exon 37 156 phase 0
  Exon 458 659 phase 0
  Exon 744 796 phase 1
//
>3052266-3053162.[37:796].sp.tr
MAGPSLACCLLGLLALTSACYIQNCPLGGKRAAPDLDVRKCLPCGPGGKGRCFGPNICCA
EELGCFVGTAEALRCQEENYLPSPCQSGQKACGSGGRCAVLGLCCSPDGCHADPACDAEA
TFSQR
//
>3052266-3053162.[37:796].sp
ATGGCCGGCCCCAGCCTCGCTTGCTGTCTGCTCGGCCTCCTGGCGCTGACCTCCGCCTGC
TACATCCAGAACTGCCCCCTGGGAGGCAAGAGGGCCGCGCCGGACCTCGACGTGCGCAAG
TGCCTCCCCTGCGGCCCCGGGGGCAAAGGCCGCTGCTTCGGGCCCAATATCTGCTGCGCG
GAAGAGCTGGGCTGCTTCGTGGGCACCGCCGAAGCGCTGCGCTGCCAGGAGGAGAACTAC
CTGCCGTCGCCCTGCCAGTCCGGCCAGAAGGCGTGCGGGAGCGGGGGCCGCTGCGCGGTC
TTGGGCCTCTGCTGCAGCCCGGACGGCTGCCACGCCGACCCTGCCTGCGACGCGGAAGCC
ACCTTCTCCCAGCGC
//
```

Query protein: NP_000481.2| vasopressin-neurophysin 2-copeptin preproprotein [Homo sapiens]
Target Sequence   NC_000020.10

```
NP_000481.2        1 MPDTMLPACFLGLLAFSSACYFQNCPRGGKRAMSDLELRQ
                     MPDTMLPACFLGLLAFSSACYFQNCPRGGKRAMSDLELRQ
                     MPDTMLPACFLGLLAFSSACYFQNCPRGGKRAMSDLELRQ
c3065370-306320   51 acgaaccgttcgccgtttgtttcatcaggaagatgcgcac
                     tcacttccgttgttctcccgataagcgggagctcatatga
                     gtccggcccccagccccgcccgccggccggcgccgggag


NP_000481.2       41                        CLPCGPGGKGRCFGPSICCADELGCF
                                            CLPCGPGGKGRCFGPSICCADELGCF
                                            CLPCGPGGKGRCFGPSICCADELGCF
c3065370-306320  171 GTACTTC  Intron 1   CAGtcctgcggagcttgcaattgggcgtt
                     <0-----[171  : 1546]-0>gtcggcggagggtgcgtggcaatggt
                                            ccccccgcaccccgcccccgcggccc


NP_000481.2       67 VGTAEALRCQEENYLPSPCQSGQKACGSGGRCAAFGVCCND
                     VGTAEALRCQEENYLPSPCQSGQKACGSGGRCAAFGVCCND
                     VGTAEALRCQEENYLPSPCQSGQKACGSGGRCAAFGVCCND
c3065370-306320 1625 ggagggcctcggatcctctctgcagtgaggctggtggttag
                     tgccactggaaaaatcccgacgaacggggggggcctgtggaa
                     gcgtgggccgggccgggccgccgggcgcgcccccccctcccc


NP_000481.2      108                           SCVTEPECREGFHRRARASDRSN
                                               SCVTEPECREGFHRRARASDRSN
                               E:E[gag]        SCVTEPECREGFHRRARASDRSN
c3065370-306320 1748 GGTGCGCG  Intron 2   CAGAGatgagcgtcggtcccgcgagcaa
                      <1-----[1749 : 1922]-1>  ggtcacaggagtaggcgcgagga
                                               ccgcgcgccgctccccccccgcc


NP_000481.2      132 ATQLDGPAGALLLRLVQLAGAPEPFEPAQPDAY
                     ATQLDGPAGALLLRLVQLAGAPEPFEPAQPDAY
                     ATQLDGPAGALLLRLVQLAGAPEPFEPAQPDAY
c3065370-306320 1994 gaccggcgggtccccgccgggcgctgcgccggt
                     ccatagccgctttgttatcgccactaccacaca
                     cgggcggcgcgggggggggcggcgccgccgcccc


//
Gene 1
Gene 51 2092
  Exon 51 170 phase 0
  Exon 1547 1748 phase 0
  Exon 1923 2092 phase 1
//
>c3065370-3063202.[51:2092].sp.tr
MPDTMLPACFLGLLAFSSACYFQNCPRGGKRAMSDLELRQCLPCGPGGKGRCFGPSICCA
DELGCFVGTAEALRCQEENYLPSPCQSGQKACGSGGRCAAFGVCCNDESCVTEPECREGF
HRRARASDRSNATQLDGPAGALLLRLVQLAGAPEPFEPAQPDAY
//
>c3065370-3063202.[51:2092].sp
ATGCCTGACACCATGCTGCCCGCCTGCTTCCTCGGCCTACTGGCCTTCTCCTCCGCGTGC
TACTTCCAGAACTGCCCGAGGGGCGGCAAGAGGGCCATGTCCGACCTGGAGCTGAGACAG
TGCCTCCCCTGCGGCCCCGGGGGCAAAGGCCGCTGCTTCGGGCCCAGCATCTGCTGCGCG
GACGAGCTGGGCTGCTTCGTGGGCACGGCTGAGGCGCTGCGCTGCCAGGAGGAGAACTAC
CTGCCGTCGCCCTGCCAGTCCGGCCAGAAGGCGTGCGGGAGCGGGGGCCGCTGCGCCGCC
TTCGGCGTTTGCTGCAACGACGAGAGCTGCGTGACCGAGCCCGAGTGCCGCGAGGGCTTT
CACCGCCGCGCCCGCGCCAGCGACCGGAGCAACGCCACGCAGCTGGACGGGCCGGCCGGG
GCCTTGCTGCTGCGGCTGGTGCAGCTGGCCGGGGCGCCCGAGCCCTTCGAGCCCGCCCAG
CCCGACGCCTAC
```

3. <u>Allatostatins:</u>

Query protein: ALLS_APIME_Allatostatins
Target Sequence HarSal, contig 8088

```
genewise output
Score 180.95 bits over entire alignment
Scores as bits over a synchronous coding model

ALLS_APIME_Alla    5 TSVLTSSLAFLYFFGIVGRSALAMEETPASSMNLQHYNNMLNPMVFDDT
                     TS+    L +L ++ +VG S  AME+ P+SS+++   N +LN + +++
                     TSLTAMRLIMLCLLSVVGESTAAMEDMPSSSLHMPRLNPLLNHVEYEEP
HarSal_contig8083936 aacagaacaactctaggggtaggaggactttccacctacttacggtggc
                     cgtcctgttttgttgttgacccctaatccccctatcgtacttaataaaac
                     gtgtcggccgctggcgttaggggggtggtatgtggaataggcttgcggt


ALLS_APIME_Alla   54 MPEKRAYTYVSEYKRLPVYNFGIGKRWIDTNDNK
                       EKR+Y YVSEYKRLP+YNFGIGKRW+D N++K
                     S-EKRSYAYVSEYKRLPLYNFGIGKRWVDDNEDK
HarSal_contig8084083 t gaattgtgtgtaaccctatgagactgggagga
                     c aagcacatcaaagtctaatgtgaggtaaaaaa
                     t ggggctctcgcggaagcccccagagccccgta


ALLS_APIME_Alla   88                        RGRDYSFGLGKR-RQYSFGLGKRNDN
                                            R R +SFG+GKR R Y FG+GKRN +
                                            RTRPFSFGIGKRLRDYRFGIGKRNSH
HarSal_contig8084182 GTGAGTT  Intron 1   CAGaacctttgagacccgtatgagacaac
                     <0-----[84182:87099]-0>gcgctctgtgagtgaagtgtgagaga
                                            aagcctccccgtgaccgccacgcccc


ALLS_APIME_Alla  113 ADYPLRLNLDYLPVDN-PAFHSQE-NTDDFLEEKRGRQPYSFGLGKRAV
                           LNLDYLP DN  A+HS+E N DD++EEKR  QP+SFG+GKR
                     P-----LNLDYLPADNLEAYHSREDNADDLMEEKRSNQPFSFGIGKRGW
HarSal_contig8087178 c     cacgtccggacggtctcggagggcaggacaacctatgagacgt
                     c     tataatccaatacaacgaaacaattaaaggaactgtgtgaggg
                     c     ccgtctgccccgttctcgccgctcggggctcggcccccgaccg


ALLS_APIME_Alla  160 HYSGGQPLGSKRPN-DMLSQ-RYHFGLGKRMSEDEE
                       +G      +RPN D+++  RY + LGK + EDEE
                     KLAGAT---ARRPNADVVAAPRYLLSLGKGIGEDEE
HarSal_contig8087310 atggga   gaacagggggccttcatgagaggggg
                     atcgcc   cggcacattcccgattgtgagtgaaaa
                     ggcaga   ggaccccccttgttagcgcacacacgg


//
Gene 1
Gene 83936 87408
  Exon 83936 84181 phase 0
  Exon 87100 87408 phase 0
//
FT          CDS    join(83936..84181,87100..87408)
FT                   /note="Match to ALLS_APIME_Allatostatins"
//
>HarSal_contig8088.[83936:87408].sp.tr
TSLTAMRLIMLCLLSVVGESTAAMEDMPSSSLHMPRLNPLLNHVEYEEPSEKRSYAYVSE
YKRLPLYNFGIGKRWVDDNEDKRTRPFSFGIGKRLRDYRFGIGKRNSHPLNLDYLPADNL
EAYHSREDNADDLMEEKRSNQPFSFGIGKRGWKLAGATARRPNADVVAAPRYLLSLGKGI
GEDEE
//
>HarSal_contig8088.[83936:87408].sp
ACGAGTCTGACTGCCATGAGGCTCATCATGCTCTGTCTGTTGAGCGTGGTTGGTGAATCG
ACGGCGGCGATGGAGGATATGCCGTCTTCATCTCTGCATATGCCGCGATTAAATCCATTG
TTGAACCATGTTGAGTACGAGGAGCCTTCTGAGAAGAGGTCGTACGCTTACGTTTCCGAG
TACAAGAGGCTACCACTGTACAACTTCGGCATCGGAAAGCGATGGGTCGACGACAACGAG
GATAAAAGAACACGGCCCTTCTCTTTCGGCATCGGCAAGCGTCTGCGAGACTACAGGTTC
GGCATAGGCAAGCGCAACAGCCACCCCCTCAACCTGGATTACCTTCCGGCCGACAACCTC
GAGGCTTATCACTCTCGCGAGGACAACGCGGACGATCTCATGGAGGAGAAGCGCAGTAAC
CAGCCGTTCAGCTTCGGCATCGGGAAACGCGGCTGGAAGTTGGCCGGAGCGACAGCGAGG
AGACCCAACGCCGACGTCGTCGCTGCTCCGCGTTATTTACTGAGCTTGGGCAAAGGCATA
GGCGAAGACGAGGAG
//
```

Query protein: ALLS_APIME_Allatostatins
Target Sequence CamFlo, contig 5982

```
genewise output
Score 171.86 bits over entire alignment
Scores as bits over a synchronous coding model

ALLS_APIME_Alla    5 TSVLTSSLAFLYFFGIVGRSALAMEETPASSMNLQHYNNMLNPMVFDDT
                     +S++   L + Y++ +VGRS  A+EE PASS+++   N + + + +D+
                     SSLIAMRLIIFYLLSVVGRSTAAVEEAPASSLHIPRLNPLSSNLEYDEP
CamFlo_contig5917762 tacagaacaattctagggctaggggggcgtttcacctacttaatgtggc
                     cgttctgttttattgttggcccctaacccccctatcgtactcgataaaac
                     gtactgactcccggcctaaaaggaggagcgcgttaagtgagccggctgc


ALLS_APIME_Alla   54 MPEKRAYTYVSEYKRLPVYNFGIGKRWIDTNDNK
                       EKRAY Y+SEYKRLP+YNFGIGKRWID +++K
                     S-EKRAYAYISEYKRLPLYNFGIGKRWIDNSEDK
CamFlo_contig5917909 t gaagtgtatgtaaccctatgagactagaagga
                     c aagcacatcaaagtctaatgtgaggtaagaaa
                     t aaggctctcacggattcccccagagcctcgta


ALLS_APIME_Alla   88                       RGRDYSFGLGKR-RQYSFGLGKRNDN
                                           R R +SFG+GKR R Y FG+GKRN
                                           RTRPFSFGIGKRLRDYRFGIGKRNSG
CamFlo_contig5918008 GTGAGTT  Intron 1   CAGcacctttgagaccagtatgagacaag
                     <0-----[18008:18482]-0>gcgctctgtgagtgaagtgtgagagg
                                           agggcgctcaattgccgccaagttca


ALLS_APIME_Alla  113 ADYPLRLNLDYLPVDNPAFHSQE-NTDDFLEEKRGRQPYSFGLGKRAVH
                           L +D+  VDN  FHS+E N DDF+++KRG QP+SFG+GKR
                     Y---RPLGMDF-SVDNMDFHSREDNLDDFIDDKRGGQPFSFGIGKRGWK
CamFlo_contig5918561 t   cctgagt tggaagtctcggacggtaggacggcctatgagacgta
                     a   gctgtat ctaatatacgaaataattaaagggactgtgtgaggga
                     c   tcgtgtc gcccgctttcgtcgcctaccgcctgtctcccaaacgg


ALLS_APIME_Alla  161 YSGGQ-PLGSKRPNDMLSQRYHFGLGKRMSEDE
                        G+  +  +R ND++  +Y +GLGK +SE+E
                     LPMGEMAVSGRRLNDVVGPKYLLGLGKGLSENE
CamFlo_contig5918696 ccaggaggtgaacaggggcatccgtgagcagag
                     tctgatctcgggtaattgcaattgtgagtgaaa
                     gagcagcacagaacctccgatgctgcaaacgca


//
Gene 1
Gene 17762 18794
  Exon 17762 18007 phase 0
  Exon 18483 18794 phase 0
//
FT          CDS    join(17762..18007,18483..18794)
FT                   /note="Match to ALLS_APIME_Allatostatins"
//
>CamFlo_contig5982.[17762:18794].sp.tr
SSLIAMRLIIFYLLSVVGRSTAAVEEAPASSLHIPRLNPLSSNLEYDEPSEKRAYAYISE
YKRLPLYNFGIGKRWIDNSEDKRTRPFSFGIGKRLRDYRFGIGKRNSGYRPLGMDFSVDN
MDFHSREDNLDDFIDDKRGGQPFSFGIGKRGWKLPMGEMAVSGRRLNDVVGPKYLLGLGK
GLSENE
//
>CamFlo_contig5982.[17762:18794].sp
TCGAGTCTAATCGCTATGAGACTCATTATCTTCTACCTGTTGAGCGTCGTTGGACGATCA
ACAGCGGCGGTAGAGGAGGCACCGGCCTCGTCCTTGCATATTCCACGATTGAATCCGTTA
TCGAGCAACTTGGAGTACGATGAGCCCTCTGAAAAAAGGGCGTACGCTTACATTTCCGAA
TACAAGAGGCTACCTCTTTACAACTTCGGCATCGGAAAGCGATGGATCGACAATAGCGAG
GATAAACGAACGCGGCCGTTCTCGTTCGGTATCGGAAAACGTCTTAGGGACTACAGGTTC
GGCATAGGAAAGCGTAATAGCGGATACCGTCCCTTGGGTATGGATTTCTCGGTCGACAAC
ATGGACTTTCATTCTCGCGAGGATAACCTGGACGACTTTATAGACGACAAGCGCGGCGGT
CAGCCTTTCAGTTTCGGCATCGGAAAACGAGGCTGGAAGCTGCCAATGGGCGAAATGGCC
GTATCCGGAAGGAGACTAAACGACGTTGTCGGCCCGAAATATCTGCTCGGTTTGGGCAAA
GGACTAAGCGAGAACGAA
//
```

Query protein: ALLS_APIME_Allatostatins
Target Sequence Atta cephalotes, contig 11027

```
genewise output
Score 14.60 bits over entire alignment
Scores as bits over a synchronous coding model

ALLS_APIME_Alla  140 FLEEKRGR
                     FL EK+GR
                     FLTEKQGR
Atta_cephalotes 7487 tcagacgc
                     ttcaaagg
                     tgcaaaca


//
Gene 1
Gene 7487 7510
  Exon 7487 7510 phase 0
//
FT          CDS    7487..7510
FT                   /note="Match to ALLS_APIME_Allatostatins"
//
>Atta_cephalotes_contig11027.[7487:7510].sp.tr
FLTEKQGR
//
>Atta_cephalotes_contig11027.[7487:7510].sp
TTTCTGACCGAAAAACAAGGCCGA
//
```

Query protein: ALLS_DROME_Allatostatins (NP_524489.2)
Target Sequence NT_033777.2

```
NP_524489.2        1 MNSLHAHLLLLAVCCVGYIASSPVIGQDQRSGDSDADVLLAADEMADNG
                     MNSLHAHLLLLAVCCVGYIASSPVIGQDQRSGDSDADVLLAADEMADNG
                     MNSLHAHLLLLAVCCVGYIASSPVIGQDQRSGDSDADVLLAADEMADNG
c20590780-20586 3285 aatccgcccccggttggtagatcgagcgccaggaggggccggggaggag
                     tactacattttctggtgatcgccttgaaagggagacatttccaatcaag
                     gcctccccaggatcccccccccgatcgtgccacctctcggcccggccct


NP_524489.2       50 GDNIDKRVERYAFGLGRRAYMYTNGGPGMKRLPVYNFGLGKRSRPYSFG
                     GDNIDKRVERYAFGLGRRAYMYTNGGPGMKRLPVYNFGLGKRSRPYSFG
                     GDNIDKRVERYAFGLGRRAYMYTNGGPGMKRLPVYNFGLGKRSRPYSFG
c20590780-20586 3432 ggaagacggctgtgcgccgtataaggcgaacccgtatgcgaatcctttg
                     gaataagtagactgtgggcatacaggcgtagtctaatgtgagcgcactg
                     cccccgggggccctgaagctgcgccagcggcggctcctgcggttcccca


NP_524489.2       99 LGKRSDYDYDQDNEIDYRVPPANYLAAERA
                     LGKRSDYDYDQDNEIDYRVPPANYLAAERA
                     LGKRSDYDYDQDNEIDYRVPPANYLAAERA
c20590780-20586 3579 cgacagtgtgcgagagtagccgattgggcg
                     tgaggaaaaaaaaataagtcccaatccagc
                     gcacccccccgccgcccaggagccgacgtt


NP_524489.2      129                          RPGRQNKRTTRPQPFNFGLGRR
                                              RPGRQNKRTTRPQPFNFGLGRR
                               V:V[gtg]       RPGRQNKRTTRPQPFNFGLGRR
c20590780-20586 3669 GGTAAGTC  Intron 1   CAGTGccgccaacaacccctatgcgcc
                      <1-----[3670 : 3730]-1>  gcggaaagccgcactatgtggg
                                              atcagcgaggtgaccctcgcat


//
Gene 1
Gene 3285 3798
  Exon 3285 3669 phase 0
  Exon 3731 3798 phase 1
//
>c20590780-20586252.[3285:3798].sp.tr
MNSLHAHLLLLAVCCVGYIASSPVIGQDQRSGDSDADVLLAADEMADNGGDNIDKRVERY
AFGLGRRAYMYTNGGPGMKRLPVYNFGLGKRSRPYSFGLGKRSDYDYDQDNEIDYRVPPA
NYLAAERAVRPGRQNKRTTRPQPFNFGLGRR
//
>c20590780-20586252.[3285:3798].sp
ATGAACTCCCTTCACGCCCACCTCCTACTGCTGGCAGTTTGCTGCGTCGGCTACATCGCC
AGCTCCCCGGTAATTGGCCAGGATCAGCGCAGCGGAGACAGCGATGCCGATGTCCTGCTG
GCCGCCGACGAGATGGCCGACAACGGTGGCGACAACATCGACAAGCGGGTGGAGCGGTAC
GCCTTCGGTCTGGGACGACGGGCCTATATGTACACGAACGGCGGACCGGGCATGAAGCGC
CTGCCGGTCTATAACTTCGGTCTGGGCAAGAGGTCTCGTCCCTACTCCTTCGGACTGGGC
AAACGCAGCGACTACGACTACGACCAGGACAACGAGATCGACTACAGAGTGCCGCCAGCG
AACTACTTGGCAGCCGAGCGTGCTGTGCGACCTGGCCGACAGAACAAGCGAACGACGCGT
CCGCAACCCTTCAACTTTGGCCTGGGCCGACGT
//
```

Query protein: ALLS_APIME_Allatostatins (NP_001161181.1)
Target Sequence NC_007084.3

```
genewise output
Score 480.13 bits over entire alignment
Scores as bits over a synchronous coding model

NP_001161181.1     1 MRSRTSVLTSSLAFLYFFGIVGRSALAMEETPASSMNLQHYNNMLNPMV
                     MRSRTSVLTSSLAFLYFFGIVGRSALAMEETPASSMNLQHYNNMLNPMV
                     MRSRTSVLTSSLAFLYFFGIVGRSALAMEETPASSMNLQHYNNMLNPMV
c5801197-57879911580 aaaaaagcatatgtctttgaggatgtgaggacgttaaccctaaatacag
                     tgggcgttccgtcttattgttggcctctaaccccctataaaaattactt
                     ggcgattgagcgtcctccgcgagaggtgaaaacgtgttattctggttgg


NP_001161181.1    50 FDDTMPEKRAYTYVSEYKRLPVYNFGIGKRWIDTNDNK
                     FDDTMPEKRAYTYVSEYKRLPVYNFGIGKRWIDTNDNK
                     FDDTMPEKRAYTYVSEYKRLPVYNFGIGKRWIDTNDNK
c5801197-57879911727 tggaacgaagtatgtgtaaccgtatgagactagaagaa
                     taactcaagcacatcaaagtctaatgtgaggtacaaaa
                     cctcgtagagctcccgtggacccctgtagtgctctccg


NP_001161181.1    88                        RGRDYSFGLGKRRQYSFGLGKRNDNA
                                            RGRDYSFGLGKRRQYSFGLGKRNDNA
                                            RGRDYSFGLGKRRQYSFGLGKRNDNA
c5801197-57879911841 GTGAGAA  Intron 1   CAGagcgtttgcgacactatgcgacagag
                     <0-----[11841:12874]-0>gggaactgtgaggaagtgtgagaaac
                                            agcctgcgttaaaactcgggaaccct


NP_001161181.1   114 DYPLRLNLDYLPVDNPAFHSQENTDDFLEEKRGRQPYSFGLGKRAVHYS
                     DYPLRLNLDYLPVDNPAFHSQENTDDFLEEKRGRQPYSFGLGKRAVHYS
                     DYPLRLNLDYLPVDNPAFHSQENTDDFLEEKRGRQPYSFGLGKRAVHYS
c5801197-57879912953 gtccacacgtccggacgtctcgaaggtcggacgacctatgtgaaggcta
                     aactgtataatctaacctacaaacaattaaagggacagtgtgagctaag
                     ccgcagcgtttccctcgctcagcgcctcggagaggtcccaagggggtcc


NP_001161181.1   163 GGQPLGSKRPNDMLSQRYHFGLGKRMSEDEEESSQ
                     GGQPLGSKRPNDMLSQRYHFGLGKRMSEDEEESSQ
                     GGQPLGSKRPNDMLSQRYHFGLGKRMSEDEEESSQ
c5801197-57879913100 ggcctgtaacagacacatctgcgaaatgggggttc
                     ggactgcagcaattgagaatgtgagtcaaaaacca
                     taagatggagccgctgattcatggggcgcgggggg


//
Gene 1
Gene 11580 13204
  Exon 11580 11840 phase 0
  Exon 12875 13204 phase 0
//
>c5801197-5787991.[11580:13204].sp.tr
MRSRTSVLTSSLAFLYFFGIVGRSALAMEETPASSMNLQHYNNMLNPMVFDDTMPEKRAY
TYVSEYKRLPVYNFGIGKRWIDTNDNKRGRDYSFGLGKRRQYSFGLGKRNDNADYPLRLN
LDYLPVDNPAFHSQENTDDFLEEKRGRQPYSFGLGKRAVHYSGGQPLGSKRPNDMLSQRY
HFGLGKRMSEDEEESSQ
//
>c5801197-5787991.[11580:13204].sp
ATGAGGAGCAGGACAAGTGTTCTGACATCGAGCTTGGCTTTCCTCTATTTCTTCGGGATC
GTGGGAAGGTCAGCGTTGGCTATGGAAGAAACACCAGCCTCGTCTATGAATCTTCAACAT
TATAACAATATGTTGAATCCTATGGTGTTCGACGATACCATGCCTGAAAAGAGAGCGTAC
ACTTACGTCTCCGAGTATAAGAGGCTACCCGTCTACAACTTTGGGATTGGAAAGCGTTGG
ATCGATACCAATGACAACAAGAGAGGGCGCGACTATTCGTTCGGGCTTGGTAAACGAAGA
CAATACAGTTTCGGGCTGGGGAAACGAAACGACAACGCTGACTACCCGCTCAGACTGAAC
CTGGATTATCTTCCCGTCGACAATCCCGCGTTCCATTCCCAAGAGAACACGGACGACTTT
CTCGAGGAGAAACGGGGAAGGCAGCCTTACAGCTTCGGATTAGGGAAGAGGGCGGTGCAT
TACAGCGGTGGACAACCGTTAGGTTCGAAGAGACCGAACGACATGCTCAGTCAGAGATAT
CATTTCGGACTTGGGAAGAGGATGTCCGAGGACGAGGAGGAGTCGTCGCAG
//
```

4. Tachykinins:

Query protein: TACHY_APIME_Tachykinins
Target Sequence Atta cephalotes, contig 00331

```
genewise output
Score 280.05 bits over entire alignment
Scores as bits over a synchronous coding model

TACHY_APIME_Tac    1 MIIHSIFLLMVSITLVIAEESDN-VLFDKRAPTGHQEMQGKQNSASLNS
                     M++ S++ L V  +  +AEES N     KRAP G Q M+GK++     +
                     MLFSSVLFLAVWTSSSFAEESSNDAASAKRAPMGFQGMRGKKDLIPTVA
Atta_cephalotes10749 actatgctcggtaatttgggttagggtgacgcagtcgacgaagcacagg
                     tttgcttttctgcgcctcaaccaaccccagcctgtagtggaaattcctc
                     gtttgttcgacgttgacagaccttcgtcaagcgatatgcaaaccttgca


TACHY_APIME_Tac   49 ENFGIFKRALMGFQ                       GVRGKKNSIIND
                     E+  + KR L+ FQ                       G+RGKK+ +I D
                     EHNELSKRTLVNFQ                       GMRGKKDYLIPD
Atta_cephalotes10896 gcagctaaatgatcGTAAGCT  Intron 1   CAGgaagaagttacg
                     aaaatcagcttata<0-----[10938:11144]-0>gtggaaaattca
                     actatcaaaagttg                       tgggagttgatt


TACHY_APIME_Tac   75 VKNELFPEDINKRAPMGFQGMRGKKASFDDEYYKRAPMGFQGMRGKKSL
                      ++  F ED +KRAPMGFQGMRGKKA ++DEYYKRAPMGFQGMRGKKSL
                     FEDSYFLEDYDKRAPMGFQGMRGKKAILEDEYYKRAPMGFQGMRGKKSL
Atta_cephalotes11181 tggtttcggtgaagcagtcgaagaagatgggttacgcagtcgaagaatt
                     taacattaaaaagcctgtagtggaacttaaaaaagcctgtagtggaact
                     taccccttgcccaagagttgtgacggtaaacattatatgataagaggatg


TACHY_APIME_Tac  124 EE                       ILDEIKKKTTR--FQDSRSKDVYL
                     EE                       +L EI+K+     F  +R K  Y+
                     EE                       VLSEIEKRAASLGFYGTRGKKTYI
Atta_cephalotes11328 ggGTAAGCG  Intron 2   CAGgcagagaaggttgttgaagaaata
                     aa<0-----[11334:11673]-0>ttgataagccctgtagcggaacat
                     gg                       ggtatagatgggcttttaaaaatt


TACHY_APIME_Tac  148 IDYPEDYGKRVLSMDGYQNILDKKDELLGEWEKRAPMGFYGTRGKKIIL
                     ++YP+DY KR+L+M+ +Q++ +K  E   EWEKRA MGF G RGKK +
                     FEYPQDYEKRLLAME-FQDMHNKIKE---EWEKRASMGFQGMRGKKALY
Atta_cephalotes11746 tgtccgtgaactgag tcgacaaaag   gtgaagtagtcgaagaagtt
                     taacaaaaagttcta taataaataa   agaagcctgtagtggaacta
                     cgtaatcagataaga catgctgaaa   agaagttgatgggacgaagt


TACHY_APIME_Tac  197 DALEELDKRGVMDFQ                       IGLQRKKDTTF
                     D +EEL+KR +M FQ                         ++ KKD  F
                     DEIEELEKRTLMGFQ                       G-MRGKKDG-F
Atta_cephalotes11881 ggaggcgaaacagtcGTAAATT  Intron 3   TAGg aagaagg t
                     aataataagcttgta<0-----[11926:12096]-0>g tggaaag t
                     caaaatgaactgttg                       c gacgacc t


TACHY_APIME_Tac  223 DDYLDYAINP--FDYEKR-STDFQDVESGSESFKRARMGFHGMRGKRDA
                     ++Y+DY I+    D++KR S  FQ +    ++ KRA MGF GMRGKR
                     ENYIDYYIDDPDMDFDKRASMSFQGMRGKKDTDKRAPMGFQGMRGKR-S
Atta_cephalotes12124 gatagttaggcgagtgaagtaatcgaagaagagaagcagtcgacgaa a
                     aaataaataacatataagcctgtagtggaaacaagcctgtagtggag g
                     atcattcattccgttcggaagttaggacgacctgaaggttatggcgg t


TACHY_APIME_Tac  269 AGIYGSNSSTVGTIFGYQ                          MRNRG
                      G     S+  G +  YQ                          M NR
                     VGQRFEPSTNFGPLNEYQ          D:G[gga]        MGNRR
Atta_cephalotes12268 ggcatgcaaatgctagtcGGTAAGGT  Intron 4   CAGGAagaaa
                     tgagtacgcatgctaaaa <1-----[12323:13089]-1>  tgagg
                     aaaatgccgttaaacata                          gctaa


TACHY_APIME_Tac  293 NNFPVYQVEKRSPFRYLGARGKKNPRWEFRGKFVGVRGKK
                     + +   QVEKRSPFRY   RGKKNPRWE+RG FVGVRGKK
                     HILASCQVEKRSPFRYFEMRGKKNPRWELRGMFVGVRGKK
```

```
Atta_cephalotes13107 cacgttcggactctcttgacgaaacctgtcgatgggagaa
                     attccgataagcctgatatggaaacggatggtttgtggaa
                     ctacgcacaaagtcattggctgataaggagggtgggacaa


//
Gene 1
Gene 10749 13226
  Exon 10749 10937 phase 0
  Exon 11145 11333 phase 0
  Exon 11674 11925 phase 0
  Exon 12097 12322 phase 0
  Exon 13090 13226 phase 1
//
FT          CDS    join(10749..10937,11145..11333,11674..11925,12097..12322,
FT                   13090..13226)
FT                   /note="Match to TACHY_APIME_Tachykinins"
//
>Atta_cephalotes_contig00331.[10749:13226].sp.tr
MLFSSVLFLAVWTSSSFAEESSNDAASAKRAPMGFQGMRGKKDLIPTVAEHNELSKRTLV
NFQGMRGKKDYLIPDFEDSYFLEDYDKRAPMGFQGMRGKKAILEDEYYKRAPMGFQGMRG
KKSLEEVLSEIEKRAASLGFYGTRGKKTYIFEYPQDYEKRLLAMEFQDMHNKIKEEWEKR
ASMGFQGMRGKKALYDEIEELEKRTLMGFQGMRGKKDGFENYIDYYIDDPDMDFDKRASM
SFQGMRGKKDTDKRAPMGFQGMRGKRSVGQRFEPSTNFGPLNEYQGMGNRRHILASCQVE
KRSPFRYFEMRGKKNPRWELRGMFVGVRGKK
//
>Atta_cephalotes_contig00331.[10749:13226].sp
ATGCTTTTTAGTTCGGTTCTTTTCCTGGCAGTCTGGACTAGTTCGTCATTCGCAGAGGAA
TCCTCCAATGATGCCGCGTCTGCCAAACGAGCGCCCATGGGATTTCAAGGTATGCGCGGA
AAAAAAGACCTCATTCCTACGGTCGCAGAACACAATGAACTTTCCAAAAGAACATTAGTG
AATTTTCAGGGTATGAGGGGGAAAAAGGATTATTTGATACCTGATTTTGAAGACTCCTAC
TTTCTTGAGGACTACGACAAAAGAGCGCCAATGGGTTTTCAGGGTATGAGAGGCAAGAAG
GCTATATTAGAAGACGAATATTATAAACGTGCACCTATGGGATTTCAAGGAATGAGAGGG
AAGAAATCTTTGGAGGAGGTGCTGAGTGAAATTGAAAAGAGAGCTGCGTCGTTGGGCTTT
TATGGTACTAGAGGAAAAAAAACATATATTTTCGAGTATCCACAAGATTACGAAAAGAGA
CTTTTAGCAATGGAATTCCAAGATATGCACAATAAGATAAAAGAAGAATGGGAAAAAAGG
GCTTCTATGGGATTTCAGGGGATGAGAGGCAAGAAAGCATTGTATGACGAAATAGAAGAA
CTTGAGAAAAGAACCCTTATGGGTTTTCAGGGCATGAGAGGCAAGAAAGACGGCTTTGAA
AATTACATAGATTATTACATAGATGATCCCGACATGGATTTTGACAAGAGGGCATCAATG
AGTTTTCAAGGGATGAGAGGCAAGAAAGACACCGATAAGAGAGCACCGATGGGTTTTCAA
GGTATGCGGGGCAAGAGGAGTGTAGGACAAAGATTTGAGCCCAGCACGAATTTTGGACCA
TTAAACGAATATCAAGGAATGGGCAATAGAAGACACATTCTAGCCTCGTGCCAAGTCGAA
AAACGATCGCCTTTCCGATATTTTGAGATGCGCGGTAAGAAAAATCCACGATGGGAGTTA
CGGGGGATGTTTGTGGGGGTGAGAGGCAAAAAA
//
```

Query protein: TACHY_APIME_Tachykinins
Target Sequence   NC_007076

```
NP_001011576.1     1 MIIHSIFLLMVSITLVIAEESDNVLFDKRAPTGHQEMQGKQNSASLNSE
                     MIIHSIFLLMVSITLVIAEESDNVLFDKRAPTGHQEMQGKQNSASLNSE
                     MIIHSIFLLMVSITLVIAEESDNVLFDKRAPTGHQEMQGKQNSASLNSE
4278197-430832628240 aaactatttagtaacgagggtgagttgacgcagccgacgacatgttatg
                     tttactttttctctttcaacaatttaagcccgaaatagaaaccctaca
                     gcctgctgagaacgcattaatcttaccatctgataagagagttttgcta


NP_001011576.1    50 NFGIFKRALMGFQ                       GVRGKKNSIINDV
                     NFGIFKRALMGFQ                       GVRGKKNSIINDV
                     NFGIFKRALMGFQ                       GVRGKKNSIINDV
4278197-430832628387 atgatacgcagtcGTAGAGT  Intron 1   TAGggcgaaataaagg
                     atgttagcttgta<0-----[28426:28573]-0>gtggaaacttaat
                     ctatcatgcgttg                       tgtagacgactcc


NP_001011576.1    76 KNELFPEDINKRAPMGFQGMRGKKASFDDEYYKRAPMGFQGMRGKKSLE
                     KNELFPEDINKRAPMGFQGMRGKKASFDDEYYKRAPMGFQGMRGKKSLE
                     KNELFPEDINKRAPMGFQGMRGKKASFDDEYYKRAPMGFQGMRGKKSLE
4278197-430832628613 aagctcggaaacgcagtcgaagaagttgggttacgcagtcgaagaatcg
                     aaattcaataagcctgtagtggaacctaaaaaagcctgtagtggaacta
                     atatttgctcgtaggttacgagggcgcctatcattggataagacaatta


NP_001011576.1   125 E                       ILDEIKKKTTRFQDSRSKDVYLIDY
                     E                       ILDEIKKKTTR QDSRSKDVYLIDY
                     E                       ILDEIKKKTTRVQDSRSKDVYLIDY
4278197-430832628760 gGTAAGCA  Intron 2   CAGatggaaaaaaagcgtaaaggttagt
                     a<0-----[28763:29105]-0>ttaataaaccgtaacggaatattaa
                     g                       tacgtaggtgacgtaacacgcgttt


NP_001011576.1   151 PEDYGKRVLSMDGYQNILDKKDELLGEWEKRAPMGFYGTRGKKIILDAL
                     PEDYGKRVLSMDGYQNILDKKDELLGEWEKRAPMGFYGTRGKKIILDAL
                     PEDYGKRVLSMDGYQNILDKKDELLGEWEKRAPMGFYGTRGKKIILDAL
4278197-430832629181 cggtgaagttaggtcaacgaaggtcggtgaagcagttgaagaaaacggt
                     caaagagttctagaaattaaaaattgagaagcctgtagcggaatttact
                     gattagaagggtatattctggcaagaggaaatggattaggtaattttgg


NP_001011576.1   200 EELDKRGVMDFQI                       GLQRKKDTTFDDY
                     EELDKRGVMDFQI                       GLQRKKDTTFDDY
                     EELDKRGVMDFQI                       GLQRKKDTTFDDY
4278197-430832629328 ggcgacggagtcaGTGAGTA  Intron 3   CAGgtccaagaatggt
                     aataaggttatat<0-----[29367:29636]-0>gtagaaacctaaa
                     gattaactgttaa                       tgatgatggcctt


NP_001011576.1   226 LDYAINPFDYEKRSTDFQDVESGSESFKRARMGFHGMRGKRDAAGIYGS
                     LDYAINPFDYEKRSTDFQDVESGSESFKRARMGFHGMRGKRDAAGIYGS
                     LDYAINPFDYEKRSTDFQDVESGSESFKRARMGFHGMRGKRDAAGIYGS
4278197-430832629676 tgtgaactgtgaaaagtcgggagagttacgcagtcgaagaaggggatgt
                     taactactaaaaggcataatagggactagcgtgtagtggagaccgtagc
                     attgttgttcgagtgttacaactcactaattgtttggatagtgggtcaa


NP_001011576.1   275 NSSTVGTIFGYQ                          MRNRGNNFPVY
                     NSSTVGTIFGYQ                          MRNRG+NFPVY
                     NSSTVGTIFGYQ          D:D[gat]        MRNRGSNFPVY
4278197-430832629823 ataaggaatgtcGGTACTTA  Intron 4   CAGATaaacgaatcgt
                     acgctgcttgaa <1-----[29860:29934]-1>  tgagggatcta
                     tattgagtttta                          ggtaattttat


NP_001011576.1   299 QVEKRSPFRYLGARGKKNPRWEFRGKFVGVRGKKSSLQTVF
                     QVEKRSPFRYLGARGKKNPRWEFRGKFVGVRGKKSSLQTVF
                     QVEKRSPFRYLGARGKKNPRWEFRGKFVGVRGKKSSLQTVF
4278197-430832629970 cggactctatcggcgaaacctgtcgatgggagaatttcagt
                     ataagcctgatgcggaaacggatggattgtggaacctactt
                     ataaaaccacttcatagtaagatagacaagacaagggatcc


//
Gene 1
```

```
Gene 28240 30092
  Exon 28240 28425 phase 0
  Exon 28574 28762 phase 0
  Exon 29106 29366 phase 0
  Exon 29637 29859 phase 0
  Exon 29935 30092 phase 1
//
```
