# Supplementary material for: Discovery of Defense- and Neuropeptides in Social Ants by Genome-Mining
Source: PLoS One. 2012 Mar 20;7(3):e32559. doi: 10.1371/journal.pone.0032559 (PMC3308954; doi:10.1371/journal.pone.0032559)
Supplement: Figure S1 — Ant abaecin- and pilosulin-like defense peptides. Similarity alignment of novel ant (A) abaecin-like (ABA) and (B) pilosulin-like (PIL) defense peptides with known insect peptides (UniProtKB C7AHU0, P15450, F4WAL0, Q26464, Q68Y23, Q68Y22 and A9CM07). The alignments were prepared with ClustalW2 and Boxshade. The mature peptides are presented in the box and the signal peptide cleavage site (identified by similarity) is indicated by an arrow. (PDF) [file pone.0032559.s001.pdf]

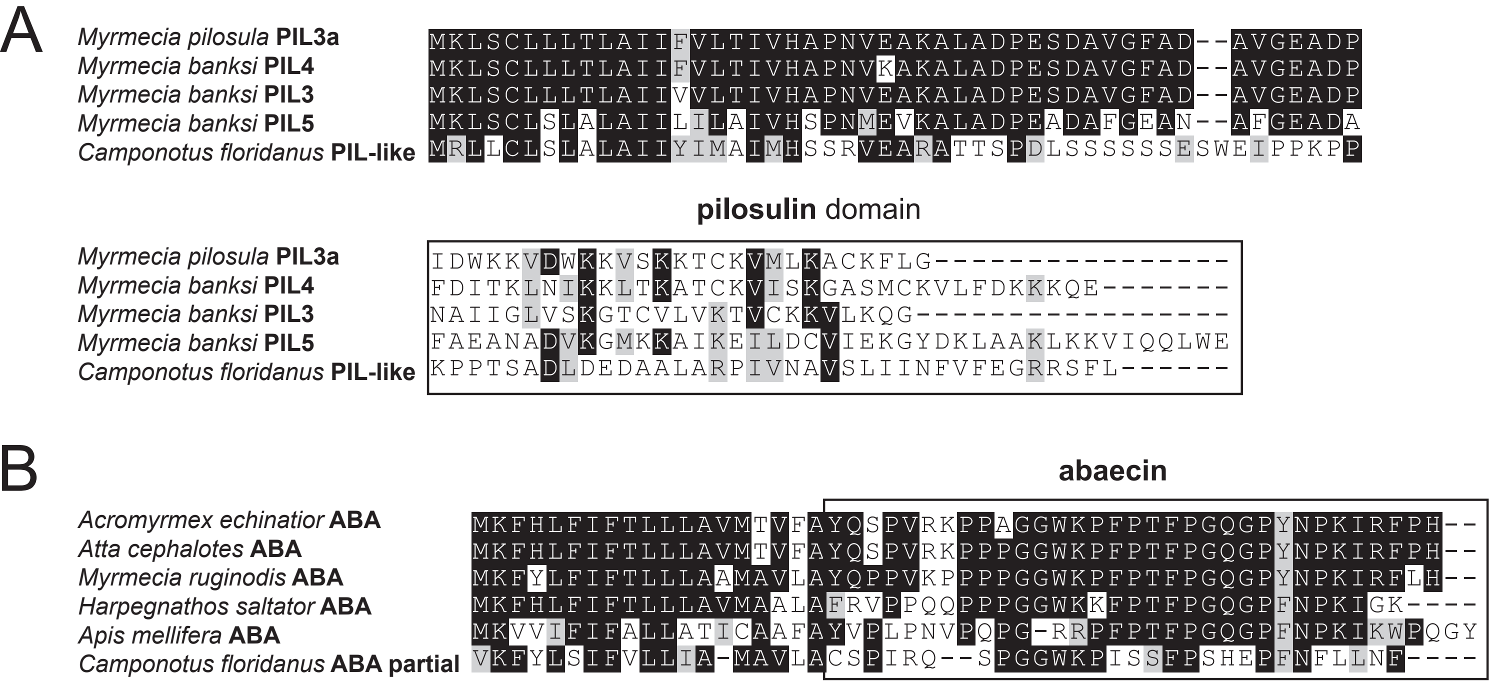

A
Myrmecia pilosula PIL3a MKLSCLLLTLAIIFVLTIVHAPNVEAKALADPESDAVGFAD--AVGEADP
Myrmecia banksi PIL4 MKLSCLLLTLAIIFVLTIVHAPNVKAKALADPESDAVGFAD--AVGEADP
Myrmecia banksi PIL3 MKLSCLLLTLAIIVVLTIVHAPNVEAKALADPESDAVGFAD--AVGEADP
Myrmecia banksi PIL5 MKLSCLSLALAIILILAIVHSPNMEVKALADPEADAFGEAN--AFGEADA
Camponotus floridanus PIL-like MRLLCLSLALAIIYIMAIMHSSRVEARATTSPDLSSSSSSESWEIPPKPP
pilosulin domain
Myrmecia pilosula PIL3a IDWKKVDWKKVSKKTCKVMLKACKFLG-----------------
Myrmecia banksi PIL4 FDITKLNIKKLTKATCKVISKGASMCKVLFDKKKQE--------
Myrmecia banksi PIL3 NAIIGLVSKGTCVLVKTVCKKVLKQG------------------
Myrmecia banksi PIL5 FAEANADVKGMKKAIKEILDCVIEKGYDKLAAKLKKVIQQLWE
Camponotus floridanus PIL-like KPPTSADLDEDAALARPIVNAVSLIINFVFEGRRSFL-------
B
abaecin
Acromyrmex echinatior ABA MKFHLFIFTLLLAVMTVFAYQSPVRKPPAGGWKPFPTFPGQGPYNPKIRFPH--
Atta cephalotes ABA MKFHLFIFTLLLAVMTVFAYQSPVRKPPPGGWKPFPTFPGQGPYNPKIRFPH--
Myrmecia ruginodis ABA MKFYLFIFTLLLAAMAVLAYQPPVKPPPPGGWKPFPTFPGQGPYNPKIRFLH--
Harpegnathos saltator ABA MKFHLFIFTLLLAVMAALAFRVPPQQPPPGGWKKFPTFPGQGPFNPKIGK----
Apis mellifera ABA MKVVIFIFALLATICAAFAYVPLPNVPQPG-RRPFPTFPGQGPFNPKIKWPQGY
Camponotus floridanus ABA partial VKFYLSIFVLLIA-MAVLACSPIRQ--SPGGWKPISSFPSHEPFNFLLNF----
